# A restatement of the natural science evidence base on the effects of endocrine disrupting chemicals on wildlife

**DOI:** 10.1098/rspb.2018.2416

**Published:** 2019-02-27

**Authors:** H. Charles J. Godfray, Andrea E. A. Stephens, Paul D. Jepson, Susan Jobling, Andrew C. Johnson, Peter Matthiessen, John P. Sumpter, Charles R. Tyler, Angela R. McLean

**Affiliations:** 1 Oxford Martin School and Department of Zoology, University of Oxford, 34 Broad St, Oxford OX1 3BD, UK; 2 Institute of Zoology, Zoological Society of London, Regent's Park, London NW1 4RY, UK; 3 Institute of Environment, Health and Societies, Brunel University London, Uxbridge UB8 3PH, UK; 4 Centre for Ecology and Hydrology, Wallingford, Oxfordshire OX10 8BB, UK; 5 Dolfan Barn, Beulah, Llanwrtyd Wells, Powys LD5 4UE, UK; 6 Biosciences, College of Life and Environmental Sciences, University of Exeter, Exeter EX4 4QD, UK

**Keywords:** endocrine disrupting chemicals, regulation, wastewater, pollution, ecotoxicology, endocrine active chemicals

## Abstract

Endocrine disrupting chemicals (EDCs) are substances that alter the function of the endocrine system and consequently cause adverse effects to humans or wildlife. The release of particular EDCs into the environment has been shown to negatively affect certain wildlife populations and has led to restrictions on the use of some EDCs. Current chemical regulations aim to balance the industrial, agricultural and/or pharmaceutical benefits of using these substances with their demonstrated or potential harm to human health or the environment. A summary is provided of the natural science evidence base informing the regulation of chemicals released into the environment that may have endocrine disrupting effects on wildlife. This summary is in a format (a ‘restatement’) intended to be policy-neutral and accessible to informed, but not expert, policy-makers and stakeholders.

## 1. Introduction

The endocrine system plays a critical role in almost all biological and physiological functions. Endocrine disrupting chemicals (EDCs) are substances (or mixtures of substances) that alter the function of the endocrine system and consequently are capable of causing adverse effects to humans or wildlife [1]. EDCs include compounds with important agricultural, industrial and pharmaceutical uses, which can become pollution problems through inadvertent human or wildlife exposure. Many different types of chemicals can be EDCs and, beyond their effects on the endocrine system, there is no single characteristic or property that they all share. Particular compounds may affect other biological processes in addition to their EDC effects. Some common natural substances may have endocrine effects (for example sugar and caffeine) but concern about EDCs in the environment chiefly focuses on synthetic chemicals that can sometimes be active at low or even very low concentrations. Though not unique to EDCs, the ability of some chemicals to be biologically active at very low concentrations raises particular regulatory issues. Timing of exposure is also critical, because EDCs may only have an effect at particular life-history stages. Many of the first EDCs to attract regulatory attention had long half-lives in the environment and became concentrated in certain species of wildlife, negatively affecting their population viability. More recently, and in addition, there has been concern about widely used substances that are active at relatively low concentrations and, though short-lived, are commonly found in the environment due, for example, to their continuous release in wastewater.

The aim of the project described here is to provide a ‘restatement’ of the natural science evidence base relevant to the design and implementation of EDC regulations to protect wildlife. We define wildlife as all non-domesticated animals, including amphibians, fish and invertebrates as well as birds, reptiles and mammals. Humans are also exposed to these chemicals when, for example, they use products containing EDCs or through contamination of food (for an introduction to EDCs and human toxicology see [1] or [2]). Toxicology studies for human health protection may anticipate issues for wildlife and *vice versa.*

Some of the most well-documented examples of wildlife population reductions caused by industrial and agricultural chemicals were due to the endocrine disrupting properties of those chemicals. The widespread use of the organochlorine insecticide DDT from the 1950s onwards was a major driver of declines in birds of prey because of reproductive failure due to eggshell thinning [3,4]. The use of polychlorinated biphenyls (PCBs) in electrical equipment and for other industrial purposes resulted in large quantities of these highly persistent chemicals entering the environment. They have become concentrated in the bodies of species at the top of ecological food webs, particularly in high-latitude regions of the Northern Hemisphere, and are linked to impaired reproduction [5,6]. Use of both types of compounds is now restricted worldwide. Not all the toxic effects of these chemicals are through endocrine disruption, but the association of EDCs with a number of classic cases of pollution affecting wildlife means that they attract particular attention from environmental protection agencies, and raise strong concerns for non-governmental organizations involved in environmental protection.

We have attempted to write this restatement in a succinct manner comprehensible to non-expert but informed readers while providing an entry into the technical literature. We have tried to be policy-neutral, though we realize that this can never be absolute. In a short summary of a very large subject it is impossible to survey all of the literature relevant to the environmental effects of the very many types of EDCs. While our review is inevitably selective, we have tried to emphasize what, in the judgement of the authors, are the generic issues of relevance to multiple EDCs.

## 2. Methods

This evidence summary was produced using a similar procedure to that used in previous restatements (e.g. [7,8]). The literature on EDCs and wildlife was reviewed and a first draft produced by a subset of the authors. At a workshop, all authors discussed and assessed the different evidence components in the light of the strength and quality of the available literature. Subsequently, using a restricted set of terms (see Appendix A, ¶ 3), each piece of evidence was assigned a code with our assessment of the nature of the evidence base.

A revised evidence summary was produced and further debated electronically to produce a consensus draft. The restatement was then sent to 28 stakeholders or stakeholder groups, including scientists involved in environmental pollution research, representatives of the pharmaceutical, water and chemical industries and non-governmental organizations concerned with environment and conservation, and UK government departments and statutory bodies responsible for environmental chemicals. We asked them to judge whether the literature had been fairly covered and that we had not inadvertently overlooked key evidence, and to review the evidence codes outlined above. We also asked the stakeholders to comment on whether the restatement achieved its aims of being policy-neutral and not a work of advocacy. The document was revised in the light of much helpful feedback. Though many groups were consulted, the project was conducted completely independently of any stakeholder and was funded by the Oxford Martin School (part of the University of Oxford).

## 3. Results

The summary of the natural science evidence base relevant to policy-making on EDCs and wildlife is given in Appendix A, with an annotated bibliography (which includes a glossary of technical terms) provided as electronic supplementary material.

## 4. Discussion

In this restatement, we have used the World Health Organization (WHO) definition (see Appendix A, ¶ 4) of an EDC. This is probably the most commonly employed, though not without issues. For example, as noted above, some substances cause adverse effects but at concentrations that would seldom, if ever, occur in the field. The WHO definition would include them in this category, but the many everyday chemicals involved are not typically considered EDCs.

Even if a chemical is shown to have endocrine disruptive activity on wildlife by the WHO definition, the chief issue for regulators is whether the substance causes harm at the population and ecological community levels. This is often challenging to determine as there are few or no baseline population data available for most species, and typically we have a poor understanding of how different factors affecting mortality and fecundity combine to influence population abundance and resilience.

If an adverse effect is observed in wildlife, determining its cause and identifying any association with a particular chemical compound can be extremely difficult. In almost all cases where an EDC (or mixture of EDCs) has caused adverse effects on wildlife, the connection with exposure to the EDC(s) has been established only after the wildlife population had declined. Improved prediction of which compounds may cause harm when released into the environment would be very helpful. One challenge is to understand how different EDCs combine to affect wildlife. At the moment the potential and actual effects of EDCs (and other chemicals) are generally evaluated on an individual basis, while wildlife populations are exposed to complex cocktails of compounds that can interact with each other [9].

Finally, we note a number of limitations of this study and discuss how it might be extended.

First, we are aware that in attempting to discuss EDCs as a category of chemicals we were unable to provide a detailed evidence summary of all the work relevant to every EDC, due to the numbers and varieties of substances involved. The rationale behind our approach was to discuss common issues relevant to many EDCs, as well as to learn lessons from particular types of chemicals that have been shown to be harmful to wildlife and have thus been banned or their use severely restricted. Restatements for specific EDCs in which the literature is more comprehensively surveyed could be produced if requested by policy-makers. Previous restatements on neonicotinoid insecticides [7,10] are examples of more targeted studies (involving pollutants that are not EDCs) where a greater coverage of the literature was possible.

The second limitation to our study is that we have focused only on the effects of EDCs on wildlife and not on humans. The reason for this is that the two issues are somewhat different. Society has a very low tolerance of harm done to individual humans, while for wildlife the impacts on populations are typically considered most important. Sources and pathways of exposure to humans and wildlife may also be different. Nevertheless, as we discuss in the restatement, the two topics are related, and bringing them together in the future in the context of the ‘One Health’ [11] agenda may be valuable.

Finally, the restatement focuses on the natural science evidence base relevant to the regulation of EDCs. Policy-makers seeking to shape regulatory regimes will also require evidence about the economic costs and benefits of different interventions, as well as their political and social acceptability. Performing economic cost-benefit analyses in this area is complicated because of the need to include not only the direct financial impact of regulation (or lack thereof) on industry, consumers and government, but also the direct and indirect economic consequences of the effects of EDCs on human health and the state of the environment. There are also other topics in the social sciences and humanities, including the history and political economy of regulating pollutants in the environment, where research may be valuable to policy-makers in understanding the desirability and acceptability of different modes of chemical regulation to society [12].

## Supplementary Material

Annotated Bibliography

## Data accessibility

This article has no additional data.

## Appendix A

**(A) Introduction and aims**

1. Endocrine disrupting chemicals (EDCs) are substances that alter the function of the endocrine (hormone) system of humans and animals, leading to adverse effects on individuals or populations. Some EDCs, singly or as mixtures, can cause negative effects at very low concentrations and include pollutants that have been shown to severely harm various species of wildlife.
2. The restatement aims to summarize the science evidence base contributing to the development of policy on the impact of EDCs on wildlife. Effects on human health are not covered. It does not attempt to survey comprehensively all evidence relating to every class of EDC, but highlights key generic issues of relevance to policymakers.
3. An assessment by the authors of the nature of the different evidence components is provided. We use the following descriptions, which explicitly are not a ranking, indicated by abbreviated codes.
  — **[B]** Background material, for example describing basic chemistry, legislation etc.
  — **[S]** Strongly supported by a substantial evidence base where further information is unlikely to change the current consensus.
  — **[L]** Less strongly supported by the existing evidence base and where further information might alter the current consensus.
  — **[E]** Expert opinion based on information from related substances or general principles from different fields of science.

**(B) What endocrine disrupting chemicals are**

This section defines EDCs and gives some examples of better-studied classes, though without providing a comprehensive catalogue.

4. We follow the World Health Organization (WHO), which defines an EDC as ‘an exogenous substance or mixture that alters function(s) of the endocrine system and consequently causes adverse health effects in an intact organism, or its progeny, or (sub) populations’ **[B]**.
  a. The endocrine system is made up of the glands and other tissues that secrete hormones: molecules that transmit information within the body **[B]**.
  b. Hormones can be biologically active at very low concentrations, often in the parts per trillion (ppt, 10^−12^) to parts per billion (ppb, 10^−9^) range **[B]**.
  c. The WHO definition applies to both human and wildlife. It implies that for a substance to be an EDC, it must have an adverse effect on the organism. The demonstration that a chemical alters endocrine function is not enough without harm being demonstrated (other definitions do not have this requirement). Whether harm to the individual affects population viability is a critical question in assessing the ecological effects of EDCs **[B]**.
  d. The WHO refers to substances that possess properties that might be expected to lead to endocrine disruption in an intact organism, its progeny or (sub)populations as ‘potential EDCs’ **[B]**. The European Food Safety Authority (EFSA) refers to substances that interact or interfere with the endocrine system, but do not lead to adverse effects, as ‘endocrine active substances’ **[B]**.
  e. Adverse effects are defined as a ‘change in the morphology, physiology, growth, development, reproduction or lifespan of an organism that results in an impairment of functional capacity, an impairment of the capacity to compensate for additional stress or an increase in susceptibility to other influences’ **[B]**.
5. There is uncertainty about the fraction of synthetic chemicals entering the environment that are EDCs. Over 140 000 compounds have been registered under the EU regulation Registration, Evaluation, Authorisation and Restriction of Chemicals (REACH), of which roughly 30 000 are in common use. Most of these have not been tested for endocrine disrupting effects in the laboratory, and fewer have been investigated *in vivo*. The number of chemicals so far found or suspected to have ED effects is 800–1000 **[L]**.
  a. High-throughput laboratory screens are available to test large numbers of compounds for evidence of endocrine activity (for example the US Environment Protection Agency's Tox21 programme has assessed over 10 000 compounds *in vitro* for endocrine disrupting and other adverse effects). While valuable as an initial screen, the assays can produce false negatives and false positives and cannot cover all the ways that EDCs may harm wildlife **[L]**.
  b. The degradation products and metabolites of EDCs may also be EDCs. While their effects will be observed during *in vivo* testing, these metabolic products are less frequently subject to testing *in vitro*. Furthermore, *in vitro* testing systems do not necessarily capture the processes of metabolism that EDCs undergo in an intact organism. Cases are known where secondary products are more potent EDCs, or are present at higher concentrations in the environment, than the parent molecule **[S]**.
6. EDCs in the environment may be (or be derived from) molecules specifically used because of their effects on the endocrine system in humans or wildlife (for example, certain steroid contraceptives, other pharmaceuticals and some pest-control products) or they may be used for completely different purposes with their EDC activity being coincidental (examples include compounds used as plasticizers or flame retardants, and in personal care products) **[B]**.
  a. While most problematic EDCs are synthetic chemicals, some are natural. For example, the thyroid disrupter perchlorate (¶ 11.e) occurs in natural mineral deposits, while phyto-oestrogens in plants (which may have oestrogenic or anti-oestrogenic effects) can enter the environment from pulp mill effluents (¶ 13.c) **[B]**.
  b. Some EDCs can also be classified ‘persistent organic pollutants’ or POPs—highly stable, typically halogenated organic compounds with high lipid solubility. Not all POPs are EDCs **[S]**.
7. The potential threat to wildlife from EDCs became widely accepted in the 1990s, leading to national or international prohibition of some substances, though in a number of cases harm was established and bans enacted before endocrine disruption was identified as the mode of action. Continuing problems result from the persistence of these substances in the environment or their continued use in some countries **[B]**.
  a. Tributyltin (TBT) was used widely in antifoulant boat paints. Observations of masculinization and sterility of gastropod molluscs in the 1970s, especially in marinas and harbours, led to its identification as a potent mollusc EDC (though initially its mode of action was incorrectly identified) **[S]**. It persists in anaerobic (low oxygen) marine sediments from which it can re-enter the water body and harm molluscs **[S]**. National then global bans (2008) have reduced amounts of TBT in the environment to a level that has allowed many marine mollusc populations to recover **[S]**.
  b. DDT (Dichlorodiphenyltrichloroethane) is an organochlorine insecticide that was used widely in agriculture before bans were introduced in different countries from the 1960s due to its persistence and impacts on both human health and wildlife. Ornithologists observed high incidences of egg shell breakage in nests of birds of prey that were spatio-temporally correlated with DDT use. Experiments (on other bird species) confirmed that the DDT metabolites (DDE and DDD) reduced the reproductive success of birds of prey through egg-shell thinning, probably caused by endocrine disruption in the shell-producing gland, though the precise mechanism is still not clear **[S]**. A global ban was instituted in 2001, though restricted application in disease vector control is still permitted **[B]**. This has reduced levels of DDT in the environment and has contributed to the recovery of a number of bird of prey populations in Europe and North America **[S]**; however, continuing high levels of DDT in Adélie penguins are probably due to its recent release from glacial meltwaters **[E]**.
    i. Many other organochlorine pesticides (for example dieldrin, endosulfan and dicofol) were also restricted globally or regionally and have subsequently been shown to be EDCs **[L, S]**. An accidental spill of dicofol into a Florida lake was followed by declines in alligator numbers that have been attributed to its endocrine-mediated effects **[L]**.
  c. Polychlorinated biphenyls (PCBs) were used widely in industry, particularly in the manufacture of electrical equipment. They impair reproductive and other endocrine functions. From the late 1960s they were found to be present at concentrations high enough to cause toxic effects in many species of wildlife **[S]**. Concerns for wildlife were raised when farmed mink feeding on Lake Michigan coho salmon suffered reproductive failure due to high levels of PCB in their food **[S]**. Very high PCB concentrations in Arctic predator and some cetacean and seal populations in European waters have been correlated with long-term population declines and low rates of reproduction **[S]**. There are numerous types (congeners) of PCBs which differ in their persistence and endocrine properties. Regional and, from 2001, global bans were introduced, subsequent decline of PCBs and other persistent organic pollutants (e.g. DDT ¶ 7b) have been linked to improvements in reproductive success and higher populations of fish-eating vertebrates such as grey seals, otters and fish eagles in northern Europe. However, as they are highly persistent in the environment and continue to pose a threat to some wildlife species.
    i. For example, no orca calves have been observed in 25 years in a population from north-west Scotland and west Ireland where levels of EDCs (in particular, PCBs) are above the toxic equivalency factor (¶ 19.g.ii) expected to have adverse effects, and higher than those seen elsewhere in the world **[S]**. A recent modelling study predicts that 40% of global orca populations face extinction in the next 100 years due to PCBs. Populations near sources of pollution and those which feed higher in the food chain are most at risk **[L]**.
8. Natural oestrogens and those used in human contraceptives (which may be the same compounds found in humans and other animals or synthetic molecules with similar actions) raise concern because of the amounts entering the environment via wastewater, the cumulative effect of multiple substances (see ¶ 19.g) and/or because they have effects on wildlife at very low concentrations.
  a. The effects on wildlife of both natural oestrogens (oestrone, E1; oestradiol, E2; and oestriol, E3), and synthetic oestrogens (17α-ethinyl oestradiol, EE or EE2) used in the contraceptive pill were first noticed in the 1980s when feminized male fish were seen in rivers near municipal wastewater outflows **[S]**. This observation prompted experiments that demonstrated the presence of oestrogens in the environment were the cause **[S]** (also see ¶ 24.a).
  b. Comparison of rivers upstream and downstream of wastewater treatment plants have frequently demonstrated increased feminization (intersex) downstream **[S]**. The degree to which this affects population densities is not clear, and complicated to determine in species stocked for angling **[E]**. Studies comparing fish population density in rivers with and without wastewater plants have not found differences, though determining how different environmental factors affect fish abundance is challenging **[L]**.
    i. There were no differences apparent in the effective population size of roach living in five river catchments with differing levels of wastewater effluent **[L]**.
  c. Livestock waste is a further source of oestrogens in the environment **[S]**. The impacts are similar to those caused by human-derived oestrogens from wastewater treatment plants **[S]**.
9. Many pharmaceuticals have endocrine effects. While these have the potential to harm wildlife, their use is relatively unrestricted because of their human health importance **[B]**. Some examples of high-volume pharmaceuticals include:
  a. Metformin is extensively prescribed, primarily for Type II diabetes; large amounts enter the environment via municipal wastewater. One study has suggested that it may act like a feminizing oestrogen in fish, but this is not proven **[L]**. Use is expected to increase with the growing incidence of Type II diabetes **[E]**.
  b. Use of anti-depressants is increasing. Fluoxetine (a selective serotonin reuptake inhibitor [SSRI] marketed as Prozac) is present in municipal wastewater and in the environment. Possible effects on wildlife have been investigated, but it is not yet clear whether typical concentrations are high enough to cause adverse effects **[L]**. SSRIs mediate a change in neurotransmitter balance with secondary effects on the endocrine system, and there is debate as to whether SSRIs should be considered EDCs **[L]**.
  c. Bicalutamide and cyproterone acetate are the most commonly prescribed anti-androgens for prostate cancer, and so of potential concern as EDCs in wildlife. Modelling of likely concentrations in UK rivers, and experimental studies on fish, indicated that harm was unlikely to occur at present rates of use **[L]**.
  d. The progestins are a class of drugs that have similar effects to the hormone progesterone **[B]**. They are used for contraception, in hormone replacement therapy and as cancer drugs **[B]**. Individual progestins at concentrations similar to those predicted to occur in UK rivers have been shown in laboratory experiments to have adverse effects on fish and frog reproduction **[S]**, and it is likely that the effects of different types of progestin will combine additively **[E]** (¶ 19.g).
10. Veterinary pharmaceuticals used in agriculture, in particular those used in relatively large quantities for economic reasons (for example, growth promotion) rather than health reasons, may become environmental EDCs (see also ¶ 12.a) **[S]**.
  a. For example, trenbolone, an anabolic steroid used in the USA and some other countries (banned in the EU) to increase muscle growth in beef cattle, is found in agricultural run-off at concentrations that may affect fish and frog reproductive physiology **[S]**.
11. A number of EDCs with the potential to affect wildlife continue to be used in industry because the extent of their harm is not certain or because of the lack of economically viable, non-EDC substitutes **[E]**.
  a. Bisphenol A (BPA) is found in many plastic products and enters the environment through wastewater and product disposal **[B]**. There is evidence showing that it can affect reproduction (due to its oestrogenic properties) **[S]** and that it can also affect neurodevelopment (perhaps through perturbations of thyroid hormones) **[L]**. BPA may have effects on some wildlife at concentrations regularly observed in the environment **[E]**. Human health concerns have led to some restrictions (such as use in baby-feed bottles) and to its recent classification as a substance of very high concern (SVHC) under EU REACH (¶ 26) legislation **[B]**.
  b. Phthalates are plasticizers used to make plastics more pliable and in personal care products. They are among the most abundant man-made chemicals in the aquatic environment, entering through municipal wastewater, sewage sludge application and poor industrial waste disposal **[S]**. Some phthalates are considered to be risks to human health because of the effects on reproductive and thyroid endocrine systems, and their use in children's toys is restricted in the EU and other jurisdictions **[B]**. In the laboratory, some phthalates have been shown to have endocrine effects in wildlife, but at above environmentally relevant doses **[L]**.
  c. Polyfluoroalkyl and perfluoroalkyl substances (PFASs) are used in a variety of industrial processes as well as in fire-fighting foam. Some can affect reproductive and growth endocrine systems, and their presence in humans led to a ban on certain (‘long-chain’) forms. While they are persistent and bioaccumulate (¶ 17) **[S]**, evidence about whether they cause harm is limited **[E]**. Other (‘short-chain’) PFASs continue to be used in industry **[B]**.
  d. Polybrominated diphenyl ethers (PBDEs) are used as flame retardants in furnishings and electronics, and can have adverse effects on thyroid hormone function **[S]**. They are persistent and bioaccumulate (¶ 17) and have been found in wildlife throughout the world, and some types (the penta-, octa- and deca- forms) are now banned under international conventions or regional legislations **[B]**.
  e. Perchlorate is an oxidizing agent used in solid rocket fuels and is also naturally present in some mineral deposits mined as fertilizers **[B]**. It has been shown to interfere with iodine uptake by the thyroid gland in amphibians, leading to retardation of metamorphosis and reduced growth rates. Thyroid disruption has been demonstrated in some North American wildlife near rocket launching facilities **[L]**.
  f. Nonylphenol is a breakdown product of alkylphenol ethoxylates which are used in manufacturing as surfactants **[B]**. Nonylphenol and other alkylphenols bind to the oestrogen receptor and have been shown to cause feminization in fish **[S]**. Nonylphenol ethoxylate has been classified as a ‘priority hazardous substance’ under the EU Water Framework Directive (¶ 26.b) and its use is restricted in the European Union. However, in other jurisdictions, particularly in industrial areas, concentrations in rivers can be high enough that negative effects on wildlife are very likely **[E]**.
12. There is uncertainty about whether some substances that are known to be harmful to wildlife should be classified as EDCs. This is significant as some jurisdictions have specific regulations governing EDC use.
  a. Diclofenac is a non-steroidal anti-inflammatory drug (NSAID) used in human and veterinary medicine **[B]**. It was used to alleviate suffering in cattle in India (where it is now banned). Severe declines (greater than 95%) in vulture populations occurred due to feeding on the carcasses of treated cattle **[S]**. Initially thought to be an EDC, diclofenac is now known to cause visceral gout and kidney failure through interfering with uric acid transport **[S]**.
  b. Atrazine is a herbicide used for agricultural weed control in many countries (it is no longer registered in Europe due to concerns about ground water contamination) and enters the environment via spray drift and agricultural run-off **[B]**. There is some evidence atrazine may lead to feminization of amphibians (and possibly other vertebrates) **[L]**. Atrazine may also reduce amphibian metamorphosis success, an indication of thyroid hormone disruption **[L]**. The evidence for the endocrine disruptive effects of atrazine under field conditions is highly contested and politicized, with examples of failure to replicate results and accusations of bias directed at both industry and non-industry researchers **[E]**.

**(C) How endocrine disrupting chemicals enter and persist in the environment**

This section describes how EDCs derived from human activity enter the environment and their subsequent fate. These pathways are largely shared with other non-EDC pollutants.

13. EDCs can enter the environment from point and diffuse sources. The former includes sites where treated and untreated wastewater (via domestic, hospital and industrial sources) is routinely discharged into rivers, waste landfill sites, and incidences of accidental pollutant release. The main diffuse sources are pesticide spray drift and agricultural runoff containing agrichemicals and compounds derived from animal manure. Use of wastewater sludge on agricultural land can be a source of EDCs derived from pharmaceuticals, personal care products and household chemicals such as PBDEs (¶ 11d) used as flame retardants **[S]**.
  a. Wastewater and landfill leachate contain many different EDCs that vary in time and space **[S]**.
  b. Commercial export of waste, in particular to countries with weak environmental protection, can result in new sources of EDCs entering the environment **[S]**.
  c. Pulp mill effluent contains a range of chemicals, and its composition depends on the processes used and tree species; many plant steroids are endocrine-active substances **[B]**. Male-biased sex ratios, increases in the expression of male secondary sexual characteristics, changes in fish mating behaviour and decreases in egg production have been found in various species of fish living downstream of pulp mills or experimentally exposed to pulp mill effluent **[S]**.
14. The level of dilution influences the effects of EDCs entering the environment via wastewater discharge. Domestic wastewater can form a high fraction of flow where population densities are high alongside rivers of modest dilution capacity (for example as in much of the UK). Dilution is greater in marine environments, though less so in harbours and shallows seas compared with major oceans **[S]**.
15. Once in the environment, some persistent EDCs (in common with other pollutants) are volatilized and transported over long distances in the atmosphere before redeposition, or are moved long distances by ocean currents **[S]**.
  a. Persistent EDCs (and some other substances) tend to accumulate at high latitudes because cold oceans hold more dissolved gases, because biodegradation rates are slower at low temperatures and because the probability of re-volatilization into the atmosphere is lower in cold regions **[S]**.
16. EDCs (like other compounds) vary considerably in the rate at which they are broken down in the environment **[S]**. The half-life of EDCs such as natural oestrogens (¶ 8) is a few days in the aquatic environment while TBT (¶ 7.a) and some forms of PCBs (¶ 7.c) and other organochlorines (¶ 7.b) can persist for decades in soils and sediments **[S]**.
  a. Concentrations of EDCs in the environment are a dynamic balance between release and breakdown. Persistent compounds break down slowly in the environment but those with rapid turnover (for example BPA [¶ 11.a] or the synthetic oestrogen EE2 [¶ 8]) may still occur at concentrations that may have effects on wildlife if they are continuously released at a sufficiently high rate (termed *pseudopersistence*) **[B]**.
  b. EDCs can be sequestered in parts of the environment where breakdown or dilution is reduced (for example, in sediments, glacial ice or the deep oceans) and then released at a later time from what become secondary sources of legacy pollutants.
  c. EDCs used in industry and construction (in particular PCBs [¶ 7.c] and fire retardants [¶ 11.d]) can enter the environment later at the time of disposal or demolition of the buildings into which they are incorporated (¶ 31) **[S]**.
17. EDCs vary in the degree to which they can persist in animal bodies (as do non-EDC contaminants). Lipophilic molecules (which associate with fat) tend not to be excreted and so can increase in concentration (*bioaccumulation* or *bioconcentration*). Predators can acquire EDCs from their prey and their concentrations often increase higher in the food web (*biomagnification*). The two processes of bioaccumulation and biomagnification explain the high concentrations of some EDCs in long-lived apex predators when compared with levels in the environment **[S]**.
  a. Concentrations of persistent EDCs (e.g. PCBs ¶ 7.c, DDT ¶ 7.b) tend to increase with age in many vertebrate species and so can be particularly high in long-lived individuals **[S]**.
  b. Due to biomagnification, the amount of PCBs (¶ 7.c) in 200–300 g of salmon flesh in Lake Ontario has been estimated to be equivalent to that in ‘several million litres of lake water’ **[S]**.
  c. In mammals, lipophilic EDCs can be transferred to offspring during gestation and lactation. Marine mammals have a very high milk fat percentage leading to substantial lipophilic EDC transfer, particularly in those species that have a relatively long lactation period (for example, cetaceans and polar bears) **[S]**.

**(D) How we know if an endocrine disrupting chemical is a problem in wildlife**

This section describes how evidence is obtained about the potential endocrine disrupting properties of different chemicals in wildlife. The section begins with studies in the laboratory and moves to information collected in the field.

18. Some substances used in industry and medicine are identified as EDCs when tested for their potential toxicity to human health. As endocrine pathways tend to be highly conserved across different types of organisms such studies are informative in identifying potential threats to wildlife (termed *read across*). Permissible human exposure levels are conservative, and bans and restrictions to protect human health will indirectly benefit wildlife **[B]**.
  a. Animals distantly related to humans and other vertebrates, and which have very different physiologies, may show unexpected effects not seen in vertebrate toxicity screens. An example is the strong effect of TBT (¶ 7.a) on reproduction in molluscs **[S]**.
  b. Read across refers to intrinsic risk and can be less informative where wildlife and human exposure are very different. For example, many EDCs occur at relatively high concentrations in aquatic environments where absorption across the epidermis or gills may cause harm not anticipated by laboratory tests with EDCs administered orally to terrestrial animals **[B]**.
19. Laboratory experiments can be carried out to assess the potential endocrine-mediated harm of varying concentrations of a chemical on different animal species (*in vivo*) or in cell culture assays (*in vitro* testing). The experimenter may measure change in hormone levels directly or effects on a *biomarker* (see ¶ 22.a) or an *endpoint* (for example an effect on behaviour, fecundity, growth, disease resistance or survival). The results will be influenced by duration and mode of exposure (for example whether in the animal's diet or environment), and by the sex and development stage of the animals used. Chronic effects of long-term exposure are more difficult to study compared with acute effects, and are typically estimated using standardized short-term or longer-term assays. Standardized multigenerational assays have been developed for a very limited range of species including species of small fish, rodents and some invertebrates **[B]**.
  a. Only a limited set of species can be maintained in the laboratory (legally and logistically), and work on many species of most concern (for example, endangered species or large vertebrates) is typically impossible. Even for those species that can be maintained in the laboratory it will not be possible to measure all potentially relevant endpoints. Extrapolation of results from laboratory to wildlife species must therefore be made with caution. Logistic and welfare considerations limit the number of animals that can be used in an experiment, reducing the statistical power to detect small effects **[B]**.
  b. Experiments using a range of exposure concentrations are used to calculate ‘no observable adverse effect levels’ (NOAEL) or ‘lowest observable adverse effect levels’ (LOAEL), which are then used to define ‘safe’ thresholds. Non-linearities in the dose–response relation and the difficulties of statistically estimating weak effects may lead to these levels being either over- or under-estimated **[B]**.
  c. Effects observed in the laboratory are often termed ‘environmentally relevant’ if they involve concentrations that have been recorded in the field. In using such a term (or a similar expression), it is desirable to distinguish between peak concentrations (for example near a wastewater effluent site or at a particular time of year) and more typical concentrations in the broader environment and over all seasons **[E]**.
  d. Non-monotonic dose responses (NMDRs) occur where the harmful effect of an EDC increases (or decreases) at both low and high concentrations. There are some laboratory reports of NMDRs from *in vitro* experiments and *in vivo* biomarkers **[L]**. However, further studies are needed to confirm the reproducibility of these observations, which would have implications for testing strategies and risk assessment **[E]**.
  e. The adverse effects caused by chronic low-level exposure (‘low-dose effects’) may not necessarily be predictable from the effects of higher test doses over shorter exposure times. Current chronic ecotoxicity tests generally include lower concentrations than those used in the past **[E]**.
  f. There is strong evidence for maternal transfer of some EDCs to offspring via eggs in fish, amphibians, reptiles and birds, or via milk or across the placenta in mammals **[S]**. There is weaker evidence for other transgenerational effects **[L]**.
  g. Wildlife species are exposed to complex mixtures of chemicals. There is evidence that EDCs with similar endocrine action combine additively to affect laboratory model animals **[S]**, and those with opposing effects (for example, masculinizing and feminizing compounds) may counteract each other **[E]**. Overall, knowledge of how EDCs interact with each other and with other pollutants is limited **[E]**.
    i. For example, five steroid pharmaceuticals, each at levels below the NOEAL, led to a reduction in the numbers of eggs produced by fish when present together (consistent with a model of independent action).
    ii. Indices such as oestradiol equivalence for oestrogenic compounds or the toxic equivalency factor for PCBs and related compounds are used to assess the additive effect of defined classes of EDC.
20. Statistical, physiological and population dynamic models can be used to extrapolate laboratory data to estimate individual and population harm **[B]**.
  a. Understanding how molecular and physiological effects of EDCs observed in the laboratory relate to individual harm in the field can be difficult. For example, a dose that under relatively benign laboratory conditions causes minor harm may have a more major effect in the wild where animals are subject to other biological and non-biological stressors **[E]**. Conversely, laboratory tests may expose animals to constant concentrations of a substance that they may encounter intermittently in the field **[E]**.
  b. Wildlife populations may be exposed to highly variable levels of EDCs, possibly restricted to certain life-history stages, factors that complicate extrapolation of laboratory data to the field **[E]**.
  c. Translating the harm done to individuals to effects on population size and viability is hard as it requires knowledge of the species' ecology. For example, if the size of a population is limited by food then the deaths or reproductive failure of some individuals may not cause significant population decline as the survivors have more food. In contrast, if a population is near a threshold size for viability (possibly because of difficulties in finding mates) relatively few failures to breed could cause extinction **[E]**.
21. Direct measurements of some persistent EDCs can be made in wildlife, and the potential harm they cause inferred from laboratory experiments (typically on different species). **[B]**.
  a. The presence of the compound *per se* does not necessarily indicate an adverse effect **[L]**. Some adverse effects may only become apparent long after the compound has dissipated **[S]** and early life exposure may change sensitivities to other compounds in later life **[L]**.
  b. High and potentially harmful concentrations of highly persistent EDCs such as PCBs (¶ 7.c) occur in the tissue of some predatory birds and sea mammals due to bioaccumulation and biomagnification (¶ 17) **[S]**.
  c. Determining the effect of individual substances in mixtures of EDCs and other substances is difficult (¶ 19.g) **[E]**.
22. Observations in wildlife of hormone levels, biomarkers or pathologies associated with endocrine disruption can signal the presence of one or more endocrine-active chemicals in the environment. The signal can suggest the type of EDC involved but may not provide an indication of the specific compound **[L]**.
  a. In this context, the term ‘biomarker’ refers to something that can be measured in an organism that covaries with processes influenced by EDCs. While a biomarker change is not an adverse effect, biomarkers are valuable because they can provide an indication of endocrine effects before the effects are strong enough to adversely affect individual health or population viability. However, biomarkers may be affected by other factors in addition to EDCs and it can be difficult to determine the relationship between these changes and individual or population harm **[E]**.
    i. The most widely used biomarker in wildlife is the egg-laying vertebrate egg protein precursor vitellogenin. The presence of the protein (or transcription of the gene responsible) in males indicates exposure to oestrogenic compounds **[S]**.
  b. Interference with the hormones involved in sexual development and reproduction can result in a variety of pathologies including feminization of males, masculinization of females, intersexes (individuals showing both male and female characters), changes in mating behaviour, sex ratio biases, poor sperm viability and reproductive failure **[S]**. Compounds include TBT (¶ 7.a), DDT (¶ 7.b), PCBs (¶ 7.c) and oestrogens (¶ 8).
  c. Thyroid hormones are important in regulating the basal metabolic rate and heart rate, and for growth and development, particularly of the long bones and brain. They are also important in the control of metamorphosis timing in amphibians **[B]**. Correlations between thyroid hormone levels and different EDCs, particularly some types of PCBs (¶ 7.c) and PBDEs (¶ 11.d), have been reported in a variety of different wildlife, and there is evidence of a correlation between bone density and PCB exposure in mammals **[L]**.
  d. Instances of impaired immunity in wildlife have been associated with endocrine active substances including perfluorooctanoic acid (a PFAS) (¶ 11.c), TBT (¶ 7.a), PCBs (¶ 7.c) and trenbolone (¶ 10.a), but the extent and type of effects these chemicals have on immunity in wildlife are poorly understood **[L]**.
23. Concern about specific EDCs (e.g. DDT ¶ 7.b, PCBs ¶ 7.c) has arisen because of observations of population declines in wildlife followed by physiological and toxicological studies that have demonstrated endocrine pathologies or high concentrations of that compound. Because the association is correlational rather than experimental, establishing a causal link can be challenging **[B]**.
  a. EDCs may reduce population viability through negative effects on reproduction **[S]**. This may not be quickly recognized in long-lived species as there is little immediate effect on population size **[B]**.
24. In principle, field experiments can be used to test the effects of EDCs on wildlife populations, but these are expensive and logistically difficult to carry out with sufficient replication **[B]**.
  a. Researchers added the artificial oestrogen 17α-ethinyl oestradiol (EE2), used in contraceptive pills (¶ 8) to a lake in Canada. The resulting concentrations of EE2 varied during the year, and at their highest were approximately an order of magnitude greater than the concentrations found in typical effluents (though concentrations of all oestrogenic compounds may reach these levels). Compared with two control lakes and previous population data, one species of fish (fathead minnow) declined drastically in numbers, though the responses of the other species were more variable **[S]**.

**(E) Major European and international chemical legislation concerning endocrine disrupting chemicals**

This section briefly indicates some of the major international legislation relevant to EDCs.

25. The Stockholm Convention on Persistent Organic Pollutants (POPs) is a United Nations treaty regulating the production, use and release of POPs. While many POPs are EDCs, they are covered under the Stockholm Convention due to their persistent and bioaccumulative properties rather than because of endocrine disruption **[B]**.
  a. Chemicals are listed in three categories: Annex A, production and use to be eliminated; Annex B, use restricted; Annex C, production and unintentional release to be reduced with the goal of elimination **[B]**.
26. European Union legislation affecting EDCs differs in the degree to which it takes a hazard-based (emphasizing intrinsic endocrine disrupting properties) or risk-based (emphasizing exposure in addition) approach. Plant Protection Products (such as pesticides) and Biocidal Products legislation emphasize hazard while REACH legislation (which treats EDCs as ‘substances of very high concern’) emphasizes risk **[B]**. Under REACH, many substances, including EDCs, may be subject to ‘risk mitigation measures’, which reduce human exposure and environmental release. While EDCs are assumed to have no safe threshold, plant protection products that are EDCs may be used where human exposure is negligible (‘cut-off criteria’) **[B]**.
  a. In addition to EU laws, countries in the Union are subject to a number of global and regional conventions that apply to particular types of substance.
  b. Other European legislation relevant to EDCs includes the Water Framework Directive, which deals with pollution of ground and surface water by substances including, but not limited to, EDCs; the Environmental Quality Standards Directive (which includes a ‘watchlist’ of chemicals of potential concern) and the Cosmetics Regulation that deals with personal care products containing EDCs **[B]**. The Marine Strategy Framework Directive deals with pollution of the marine environment; this includes the monitoring of both contaminant concentrations and their biological effects.
  c. Human pharmaceuticals are regulated separately and are exempt from REACH legislation. An environmental assessment of risks associated with their production and consumption phase is required for products registered post-2004 (EU Directive 2004/27/EC) but authorization cannot be denied on environmental grounds **[B]**.
  d. The same type of compound can be treated differently by multiple pieces of legislation, for example fungicidal azoles that are used in crop protection and pharmaceuticals **[B]**.
27. Trade in electronic waste and other hazardous materials is governed by the UNEP Basel Convention on the Control of Transboundary Movements of Hazardous Wastes and their Disposal. In addition, the Strategic Approach to International Chemicals Management (SAICM) of the UNEP and the WHO is a policy framework designed to promote chemical safety globally **[B]**.

**(F) What can be done about endocrine disrupting chemicals**

This section explores the options open to policy-makers to reduce the effects of EDCs on wildlife.

28. The production and use of some EDCs that pose threats to wildlife have been banned or their use severely restricted (though often the primary motivation for a ban is risk to human health) **[B]**.
  a. Banning (or restrictions on use) leads to a reduction in environmental concentrations, though slowly in the case of more persistent molecules (with PCBs [¶ 7.c] being particularly problematic) **[S]**.
  b. There is evidence of the recovery of wildlife populations in the years (sometimes decades) following chemical bans, e.g. TBT (¶ 7.a), DDT (¶ 7.b) and PCBs (¶ 7.c).
29. Incentivizing replacements for EDCs by chemicals that do not cause endocrine disruption, or are less potent EDCs, can reduce threats to wildlife **[E]**.
  a. There are cases where proposed alternatives have subsequently been shown also to be EDCs. For example, PBDEs (¶ 11.d) were developed as a replacement for PCBs (¶ 7.c) but were found to be persistent and bioaccumulating EDCs and subsequently some types were banned. Other bromine-containing flame retardants have been developed as replacements, and have also been detected in wildlife, though without harm being demonstrated **[S]**. Similarly, there is concern that other bisphenol compounds used to replace bisphenol A (¶ 11.a) are also EDCs, although effects are only observed at concentrations higher than those currently observed in the environment **[L]**.
30. Wastewater treatment can substantially reduce the amounts of pharmaceutical and other EDCs (as well as other chemicals) entering the environment from this source. Interventions include (but are not limited to) percolation through granular activated carbon, treatment with ozone or wetland construction. Investment in wastewater treatment is determined both by decisions in the private sector and the regulatory environment put in place by government **[B]**.
  a. It has been estimated that upgrading all wastewater plants to granular activated carbon treatment with the specific aim of removing EDCs would cost €30 billion in England and Wales **[E]**. There are other energy-intensive tertiary treatments and augmented biological treatments that effectively remove EDCs **[L]**. However, cost, performance and consistency vary, and long-term evaluation is needed **[B]**.
  b. The more strategic placement of wastewater outlets and management of water levels to reduce periods of low flow could, in theory, reduce the effects of EDCs by ensuring rapid dilution, but the costs and local acceptability of new infrastructure frequently make such changes infeasible **[E]**.
31. Careful handling of waste and of material from industrial and domestic demolition can reduce the volume of EDCs entering the environment. For example, UK regulations require that persistent organic pollutants are either destroyed by incineration or chemical destruction, or are permanently stored underground **[B]**.
  a. Much material containing EDCs, in particular PCBs (¶ 7.c), PFASs (¶ 11.c), PBDEs (¶ 11.d) and other flame retardants, ends up in landfill. Choice of landfill sites and their management can reduce leaching and aerosol transport of EDCs into the environment **[S]**.
  b. Recycling facilities (e.g. for electronics and plastic waste) can be designed to reduce release of EDCs **[S]**. The presence of EDCs in many products is unknown as labelling their presence has not been required **[B]**.
  c. Large quantities of waste products enter the environment due to inadequate waste collection and poor waste processing, particularly in developing countries **[B]**. The EDCs derived from plastics and electronic waste are of particular concern **[E]**.
32. Decontamination of persistent EDCs from heavily polluted sites is possible, although complex and expensive. It is most often carried out where there is a risk to humans. There is a range of decontamination methods suitable for different substances and substrates, including incineration, bioremediation, chemical methods **[B]**.
  a. The US EPA Superfund Program has supported the remediation of almost 400 sites, many of which were contaminated with EDCs. Clean-up often involves removal of soil or sediment (by dredging in rivers or harbours) then containment of polluted material. Monitoring of remediated sites has shown improvements in indices of ecological health **[S]**.
33. Specific measures can be taken to reduce the load of EDCs derived from human pharmaceuticals (¶ 9) in sewage treatment plants **[B]**.
  a. Better assessment of possible endocrine-mediated effects on non-human animals, monitoring of sales and prescription data, and a better understanding of their passage through the body and half-life in the environment, can help predict problems to wildlife **[E]**.
  b. Pharmaceutical EDCs can enter the environment through incorrect disposal which can be reduced by drug take-back schemes (mandated in the European Union) **[E]**. It has been estimated that 5–10% of prescribed drugs are not used, and of this 12–27% are disposed of in domestic drains **[L]**.
  c. There is research into ‘green’ products that have equal therapeutic effectiveness but reduced persistence in the environment or cause less harm to wildlife **[B]**. Environmental assessments of many products are available and may be used in prescribing **[E]**.
  d. For hospital and healthcare facilities where pharmaceutical use is high, separating urine from other waste and then treating by continuous electrodialysis followed by ozone decontamination may be justified **[E]**.
34. There is no feasible way to remove most EDCs completely from the environment, or from the bodies of wildlife. Affected populations may thus benefit from special measures to reduce other stressors (for example, habitat destruction, disease, hunting or persecution) to improve population viability **[E]**.
35. Monitoring EDC levels in the environment and wildlife, and the population density and reproductive success of affected species, is important in assessing the effectiveness of policy interventions, prioritizing compounds for assessments of persistence, bioaccumulative ability and toxicity (PBT), and providing early warnings of emerging EDC problems. All wildlife populations naturally fluctuate, so extended time series are required to detect trends. Monitoring can be logistically challenging for some of the most at risk environments and species (for example, large Arctic carnivores or cetaceans) **[B]**.
  a. The UK water industry is carrying out a national programme monitoring 70 different chemicals in effluent and water bodies including some EDCs **[B]**.
  b. Long-term monitoring programmes have shown persistent organic pollutants in air, water, human tissues (blood and breast milk) and wildlife (raptors and otters) have declined, indicating bans have been effective. However, monitoring programmes do not cover all EDCs of concern (e.g. PFASs ¶ 11.c) **[S]**.
  c. Historical wildlife population data collected for other purposes, and archived environmental and animal samples, have been valuable in determining baselines and reference values to help interpret future data **[S]**.

**(G) Future opportunities and challenges**

This section explores factors affecting EDC policy that may change in the future.

36. EDC release into the environment will be affected by demographic and economic trends. Growing populations will lead to greater releases even if per capita use or consumption remains constant. Increasing wealth in developing countries will lead to higher releases associated with greater consumption **[S]**.
37. Recent (and probably future) advances in analytical chemistry will allow the detection of more chemicals at lower concentrations than is possible at the moment, and at a cost that will allow an expansion of chemical monitoring **[E]**.
  a. Increases in the amount of data on low concentrations of known or suspected EDCs in wildlife and the environment will increase demands for research on the effects of very low-level exposure on individuals and populations **[E]**.
38. Modern molecular biology techniques offer the prospect of high-throughput screening of multiple biomarkers. Interpreting the very large datasets that result, and developing predictors of harm at the individual and especially population level, is a substantial challenge **[E]**.
  a. There are no *in vivo* tests available for some types of endocrine disruption or in some species **[B]**.
39. Substantial uncertainty exists, and will continue to exist, about possible negative environmental effects of different EDCs, and the degree to which these can be predicted in advance of their introduction. The challenge to policymakers in designing regulatory regimes is to balance these risks against the economic and other benefits of employing the substance **[E]**.
  a. Modern environmental economics using concepts such as ecosystem services and natural capital may be helpful in formal cost–benefit analyses, though any regulatory framework will inevitably involve political value judgements **[E]**.
40. Climate change is likely to have both positive and negative influences on the relationship between EDCs and wildlife. Models do not completely agree on how environmental concentrations will change, particularly in the Arctic **[E]**.
  a. Higher average temperatures will increase the rate of volatilization (evaporation) and degradation of chemical pollutants including EDCs **[L]**.
  b. Reduced annual precipitation may occur in some regions, leading to lower flows and less dilution of wastewater, which will increase concentrations of EDCs **[L]**.
  c. An increase in extreme rainfall events is expected. This may affect the frequency of point-pollution episodes from farmland and from sewer overflows into river water **[L]**.
  d. Climate change may cause stress for some wildlife and lessen their ability to withstand further stresses in the form of EDCs **[S]**.
  e. Climate change may affect the feeding ecology and thus the exposure of wildlife species; for example, earlier break-up of sea-ice has led to polar bears feeding on more heavily contaminated seal species **[S]**.
  f. EDCs deposited in Arctic and glacial ice may be released upon melting **[S]**.
41. Better healthcare, ageing populations and the rise in obesity and related co-morbidities may increase the discharge of pharmaceutical EDCs (¶ 9) into the environment (though in an ageing population of constant size, e.g. East Asia, the release of EDCs associated with contraceptives may decline).
  a. Countering this, the introduction and spread of ‘green’ pharmaceuticals and personalized medicine, nanotechnology and other technologies that enable better drug targeting, and so a reduction in dose, which will reduce EDC discharges **[E]**.
42. Less developed and developing countries tend to have weaker environmental protection mechanisms, and there is typically less information about local EDC concentrations. Volatilization and long-range transport of EDCs from countries with weak regulations can have global consequences. How economic development and environmental protection proceed will determine whether their net effect as sources of EDCs increases or decreases **[E]**.
