# Supplementary material for: A restatement of the natural science evidence base on the effects of endocrine disrupting chemicals on wildlife
Source: Proc Biol Sci. 2019 Feb 27;286(1897):20182416. doi: 10.1098/rspb.2018.2416 (PMC6408895; doi:10.1098/rspb.2018.2416)
Supplement: Annotated Bibliography [file rspb20182416supp1.docx]

# Annotated Bibliography to accompany:

# A restatement of the natural science evidence base regarding the impacts of endocrine disrupting chemicals on wildlife.

Proceedings of the Royal Society B: Paper bibliographic details to add (and in header!)

---------------------------------------------------------------------------------------------------------------------------

H. Charles J. Godfray, Andrea E. A. Stephens, Paul D. Jepson, Susan Jobling, Andrew C. Johnson, Peter Matthiessen, John P. Sumpter, Charles R. Tyler & Angela R. McLean

---------------------------------------------------------------------------------------------------------------------------

Paragraph numbering corresponds to that in the main document.

## Glossary

| **Chemicals** | |
| --- | --- |
| BPA | bisphenol A, a plasticiser, C_15_H_16_O_2_. |
| DDT | dichlorodiphenyltrichloroethane, an organochloride insecticide, C_14_H_9_Cl_15_. |
| EDC | endocrine disrupting chemical |
| E1 | oestrone – one of the three major endogenous oestrogens |
| E2 | oestradiol – the primary female sex hormone |
| EE2 / EE | ethinyl oestradiol – a synthetic derivative of oestradiol |
| E3 | oestriol – one of the three major endogenous oestrogens, only produced in identifiable quantities in mammals during pregnancy |
| OC | organochloride – an organic (i.e. carbon-based) compound containing at least one covalently bonded atom of chlorine. |
| PBDE | polybrominated diphenyl ethers, C_12_H_10-x_Br_x_O. |
| PCB | polychlorinated biphenyl, chemical formula C_12_H_10−x_Cl_x_ |
| PFAS | per (or poly) fluorinated alkylated substances |
| POP | persistent organic pollutant. Chemicals that bioaccumulate and biomagnify, such as the organochloride pesticides. |
| PBT | persistent, bioaccumulative, toxic |
| TBT | tributyltin, contains a (C_4_H_9_)_3_Sn group. |
| vPvBvT | very persistent, very bioaccumulative, very toxic |
| vtg | vitellogenin |
| **Organisations / Legislation** | |
| CDC | Center for Disease Control (US) <https://www.cdc.gov/> |
| DEFRA | Department for Environment, Food & Rural Affairs (UK) <https://www.gov.uk/government/organisations/department-for-environment-food-rural-affairs> |
| ECHA | European Chemicals Agency <https://echa.europa.eu/> |
| EFSA | European Food Safety Authority <http://www.efsa.europa.eu/> |
| FAO | Food and Agriculture Organization of the United Nations <http://www.fao.org/home/en> |
| NHS | National Health Service (UK) <http://www.nhs.uk/pages/home.aspx> |
| OECD/OCDE | Organisation for Economic Co-operation and Development <http://www.oecd.org/> |
| REACH | Registration, Evaluation, Authorisation and Restriction of Chemicals <http://ec.europa.eu/environment/chemicals/reach/reach_en.htm> |
| UNEP | United Nations Environment Programme <http://www.unep.org/> |
| US EPA | US Environmental Protection Agency <https://www.epa.gov/> |
| US FDA | US Food and Drug Administration <https://www.fda.gov/> |
| WHO | World Health Organization <http://www.who.int/en/> |
| **Glossary of technical terms** | |
|  |  |
| additive | The effects of two compounds can be predicted from the effects of each individual compound alone. |
| androgen | A steroid hormone (natural or synthetic) that regulates the development and maintenance of male characteristics (i.e. masculinising hormones). An anti-androgen blocks the effects of androgens. |
| adverse effect | Defined by the WHO and FAO as a “change in the morphology, physiology, growth, development, reproduction or lifespan of an organism that results in an impairment of functional capacity, an impairment of the capacity to compensate for additional stress or an increase in susceptibility to other influences”. |
| bioaccumulation | The process in which a chemical substance is absorbed by all routes of exposure (dietary as well as dermal and respiratory paths) and thus includes food chain transfer. As the chemical is not expelled, the amount increases in the animal’s body over time, as a result the internal concentration of the chemical is greater than in the external environment. |
| bioconcentration | The process by which a chemical is absorbed by an organism only through its respiratory and dermal surfaces. As the chemical is not expelled, the amount increases in the animal’s body over time, as a result the internal concentration of the chemical is greater than in the external environment. |
| biomagnification | When the concentration of the chemical in an organism exceeds that of its diet – i.e. levels of the compound increase up the food chain, such that highest concentrations occur in top predators. |
| endocrine active | Defined by the EFSA as substances that interact or interfere with the endocrine system, but do not lead to adverse effects. |
| endocrine disrupting chemical | Defined by the WHO as “an exogenous substance or mixture that alters function(s) of the endocrine system and consequently causes adverse health effects in an intact organism, or its progeny, or (sub) populations”. |
| epigenetic | A heritable change in the function of genes that does not involve changes to the underlying DNA sequences. Epigenetic changes can switch genes on or off and determine which proteins are transcribed. |
| exposure | The measurement of both the amount of, and the frequency with which, a substance comes into contact with a person or the environment. |
| hazard | Anything that may cause harm, such as chemicals, electricity, adverse weather etc. |
| oestradiol | The most important of the three major natural oestrogens. Also called estradiol or E2. |
| oestriol | One of the three major natural oestrogens, also called estriol or E3. |
| oestrogens | The primary female sex hormones. These are steroid hormones that develop, regulate and maintain the female reproductive system (i.e. feminising hormones). There are three main oestrogens produced naturally, oestrone, oestradiol and oestriol. Oestrogens are also essential for male fertility. Synthetic oestrogens include steroidal oestrogens such as ethinyl oestradiol and non-steroidal ones such as diethylstilbestrol. An anti-oestrogenic compound blocks the effects of the oestrogens. |
| oestrone | One of the three major natural oestrogens, also called estrone or E1. |
| organic | In chemistry, organic refers to compounds containing carbon. All known life is based on organic compounds. An organic compound will also contain hydrogen and may contain a number of other atoms. |
| persistent organic pollutant | Organic chemical substances that possess a particular combination of physical and chemical properties such that, once released into the environment, they remain intact for exceptionally long periods of time (many years); become widely distributed throughout the environment as a result of natural processes involving soil, water and, most notably, air; accumulate in the fatty tissue of living organisms including humans, and are found at higher concentrations at higher levels in the food chain; and are toxic to both humans and wildlife. Source: Stockholm Convention (2008a). |
| progesterone | The primary progestogen synthesised by humans, it is involved in the menstrual cycle, pregnancy and embryogenesis. Often abbreviated as ‘P4’. |
| progestins | Synthetic progestogens with similar effects to those of the natural hormone progesterone. They have important effects on the female reproductive system and are used in hormonal birth control and menopausal hormonal therapy. |
| progestogens | Alternatively called progestagens or gestagens, a class of steroid hormone (natural or synthetic) that activate the progesterone receptor. |
| pseudopersistant | Compounds that breakdown rapidly in the environment (i.e. non-persistent) but are constantly present at concentrations that may have effects on wildlife because they are continuously released at a sufficiently high rate (typically in wastewater). |
| risk | The probability that something (person, animal, environment) will be harmed (e.g. experience an adverse health effect) if exposed to a hazard, together with an indication of how serious the harm could be. |
| vitellogenin | The protein from which the egg yolk is derived. Found in all egg-laying vertebrates. |
| wildlife | All non-domesticated animals, including amphibians, fish and invertebrates as well as birds, reptiles and mammals. |

## (A) Introduction and Aims

1. Definition given here is consistent with that of the WHO (Bergman *et al.,* 2012).
2. No references cited.
3. Categories developed by authors based on schemes used in previous Restatements (Godfray *et al.*, 2013; Godfray *et al.*, 2014; Godfray *et al.*, 2015; Dadson *et al.*, 2017; McLean *et al.*, 2017).

## (B) What are EDCs?

1. The WHO definition (Bergman *et al.*, 2012) is the most commonly used definition (Futran Fuhrman *et al.*, 2015). Vandenberg *et al.* (2016) discuss techniques to determine if a chemical meets the above definition. Solecki *et al.* (2016) discusses some of the complexities arising from the WHO definition and provides a consensus statement from a range of scientists as to how it should be interpreted. The WHO has produced two highly detailed reviews on the effects of EDCs on both humans and wildlife (Damstra *et al.*, 2002; Bergman *et al.*, 2012), as has the European Environment Agency (Weybridge Report, 1996; Weybridge+15, 2012). Matthiessen *et al.* (2018) have also recently reviewed the evidence for impacts of EDCs on wildlife populations.
   1. For an overview of the function of the different endocrine glands, see Bergman *et al.* (2012), or, for a medical focus, see Gurnell *et al.* (2010) and Weetman (2010).
   2. Vandenberg *et al.* (2012), Table 2, reports levels of different hormones in the human body. EDCs can also act at very low concentrations, e.g. TBT (Matthiessen & Gibbs, 1998).
   3. See Vandenberg (2014), Futran Fuhrman *et al.* (2015) and Zoeller *et al.* (2014) for discussion of adverse effects of EDCs. The US Environment Protection Agency (EPA) defines endocrine disrupting chemicals as “an exogenous agent that interferes with the production, release, transport, metabolism, binding, action or elimination of natural hormones in the body responsible for the maintenance of homeostasis, and the regulation of developmental processes” (Kavlock *et al.*, 1996). The EPA’s definition includes certain heavy metals not normally considered EDCs (Georgescu *et al.*, 2011; Scognamiglio *et al.*, 2016). The Endocrine Society defines EDCs as “exogenous chemical(s), or mixtures of chemicals, that interfere with any aspect of hormone action” (Gore *et al.*, 2015). Zoeller *et al.* (2014), Table 1, provides alternative definitions for endocrine disruptors; the main difference being whether “adverse effects” must be shown for the chemical to be considered an EDC. Table 2 of Zoeller *et al.* (2014) gives a range of definitions of “adverse effects”.
   4. Definition of endocrine active substances from European Food Safety Authority (2016). Definition of potential endocrine disruptor from Bergman *et al.* (2012) and Weybridge Report (1996). An example of an endocrine active substance that is not a disruptor might be sugar or caffeine at higher than usual doses (Zoeller *et al.*, 2014). All endocrine active substances have the potential to be disruptors (European Food Safety Authority, 2016).
   5. International Programme on Chemical Safety (2009).
2. Hartung (2009) and Judson *et al.* (2009) report on the numbers of chemicals in use and the numbers that have undergone safety testing. The World Health Organization (Bergman *et al.*, 2012) estimates 800 chemicals are known or suspected EDCs while the US Food and Drug Administration (FDA) estimates 1000 are (Vandenberg, 2014). Futran Fuhrman *et al.* (2015) lists databases of potential or actual EDCs. For a summary of EDCs and suspected EDCs see United Nations Environment Programme and The International Panel on Chemical Pollution (2016). The OECD ranks chemicals (including many EDCs) by global production volume (OECD/OCDE, 2009). The US endocrine disruptor screening program is a tiered testing programme for EDCs, where Tier 1 identifies endocrine active substances and Tier 2 tests those for adversity and determines the dose response (US EPA, 2017c).
   1. The Tox21 screening test (US EPA, 2017a) involves 30 different cell-based *in vitro* assays of approximately 10,000 chemical tested at 15 concentrations (Huang *et al.*, 2016; Richard *et al.*, 2016). Hartung (2009) discusses the problem of false positives and negatives.

- 1. Boxall *et al.* (2004) and Sumpter and Johnson (2005) review the presence and toxicity of degradation products. Manibusan and Touart (2017) reviews *in vitro* and *in vivo* tests. *In vitro* tests provide data on mechanism of action. For example, MBP, produced by metabolic activation of BPA, is more oestrogenic than BPA (Moreman *et al.*, 2018).

1. Actual or potential EDCs include, but are not limited to, the following: Plasticisers such as phthalates (Harris *et al.*, 1997; Oehlmann *et al.*, 2009), bisphenols (Oehlmann *et al.*, 2009; Bhandari *et al.*, 2015b), parabens (Routledge *et al.*, 1998; Boberg *et al.*, 2010); various industrial compounds: tri-butyl tin (TBT) (Sousa *et al.*, 2014), PCBs (Koppe & Keys, 2002), PBDEs (Law *et al.*, 2014; Jinhui *et al.*, 2017), PFASs (Post *et al.*, 2012), alkyl-phenols (Routledge & Sumpter, 1997; Sumpter, 2009), perchlorate (Trumpolt *et al.*, 2005), dioxins and furans (Custer *et al.*, 2005), effluent from pulp and paper mills (van den Heuvel, 2010); pharmaceuticals (Runnalls *et al.*, 2010); personal care products such as triclocarban (Chung *et al.*, 2011), triclosan (Veldhoen *et al.*, 2006); plant protection products (i.e. pesticides) such as TBT and triphenyl tin (Hu *et al.*, 2009; Sousa *et al.*, 2014), atrazine (Rohr & McCoy, 2010), organochloride (OC) pesticides (Newton, 2013; Rosner & Markowitz, 2013), glyphosate (Lanctôt *et al.*, 2013; Lanctôt *et al.*, 2014), vinclozolin (Gazo *et al.*, 2013), azoles (Matthiessen & Weltje, 2015). OC pesticides include DDT, aldrin, chlordane, chlordecone, dieldrin, endrin, heptachlor, hexachlorobenzene, lindane, mirex, pentachlorophenol and its salts and esters, technical endosulfan and its related isomers, toxaphene (Stockholm Convention, 2008a).
   1. For details on perchlorate, see ¶AB11.e and for pulp mills effluent see ¶AB13.c. The impacts of phyto-oestrogens on human health are reviewed by Zaheer and Humayoun Akhtar (2017).
   2. Definition of persistent organic pollutants from Stockholm Convention (2008b). Almost all (98%) of POPs are halogenated – i.e. some of the hydrogen atoms have been replaced by fluorine, chlorine, bromine or iodine (Scheringer *et al.*, 2012). Chlorine is the most common replacement.

1. Vandenberg *et al.* (2009) briefly review the history of the field. The Wingspread Conference in 1991 was pivotal in determining that disparate pathologies observed in a variety of organisms were endocrine-mediated (WingSpread Consensus Statement, 1995; Colborn *et al.*, 1996).
   1. For an overview of organotin chemistry and use, see Hoch (2001), Antizar-Ladislao (2008) and Sousa *et al.* (2014), and for legislation, the International Maritime Organisation (2002). (Matthiessen, 2013), Sousa *et al.* (2013), Alzieu (2000) and Santillo *et al.* (2002) provide a comprehensive overview of the history of the discovery of the effects of TBT. The mechanism of action was eventually determined to involve interactions with the nuclear retinoid-X receptor (RXR), the peroxisome proliferator-activated receptor γ (γPPAR) and their heterodimers (Sousa *et al.*, 2010; Matthiessen, 2013; Pascoal *et al.*, 2013; Sousa *et al.*, 2014; Lagadic *et al.*, 2017). The evidence associating TBT with gastropod declines is reviewed in Matthiessen and Gibbs (1998) and Sousa *et al.* (2013). Gastropod declines and/or masculinisation (imposex) has been reported from harbours and marinas globally (Matthiessen, 2013): e.g. in England (Gibbs & Bryan, 1986), France (Oehlmann *et al.*, 1996), North Sea (Hallers-Tjabbes *et al.*, 1994), Japan (Horiguchi *et al.*, 2000; Azuma *et al.*, 2014), New Zealand (Smith & Mcveagh, 1991), New South Wales, Australia (Roach & Wilson, 2009), South Africa (Marshall & Rajkumar, 2003), Morocco (Lemghich & Benajiba, 2007), British Columbia, Canada (Horiguchi *et al.*, 2004), Greenland (Strand *et al.*, 2006).

Harbour sediments as secondary sources of TBT is discussed in Antizar-Ladislao (2008).

Marine gastropod and bivalve populations have increased following the TBT ban (Smith, 1996; Colson & Hughes, 2004; Galante-Oliveira *et al.*, 2009; Morton, 2009; Birch *et al.*, 2013; Langston *et al.*, 2015).

- 1. An overview can be found in Damstra *et al.* (2002) Chapter 4. For histories for the discovery of the effects of DDT in the UK and USA, see Newton (2013) and Rosner and Markowitz (2013) respectively.

The presence of broken eggs in nests of UK raptors was first observed in the late 1940’s (Ratcliffe, 1958, 1960). Attribution of this phenomena to DDT is reported in Ratcliffe (1967) with details in Ratcliffe (1970). Ratcliffe (1970) reported that declines in eggshell thickness also occurred in a range of other species; also see Olsen *et al.* (1993). Early experiments confirming DDT as the cause of egg shell thinning are reviewed by Cooke (1973).

Populations of peregrine falcons, the world’s most widespread raptor, declined wherever DDT-induced egg-shell thinning was >17% (Newton, 2013). A reduction in productivity and/or nesting success of many other species has been spatially and/or temporally associated with DDT (often in conjunction with PCBs) including (but not limited to) bald eagles (Grier, 1982; Bowerman *et al.*, 2000; Bowerman *et al.*, 2003), sea eagles (Jensen, 1972; Roos *et al.*, 2012), osprey (Henny *et al.*, 2010), brown pelicans (Anderson *et al.*, 1975), double-crested cormorants (Weseloh *et al.*, 1983; Weseloh *et al.*, 1995), merlins (Newton, 1973; Newton & Haas, 1988), various Australian raptors (Olsen *et al.*, 1992; Olsen *et al.*, 1993). DDT and other organochloride pesticides have been found in various bird species at concentrations similar to those linked with population declines in Africa (Frank *et al.*, 1977; Yohannes *et al.*, 2014), India (Senthilkumar *et al.*, 2001) and China (Chen *et al.*, 2009).

DDT and its metabolites (DDD, dichlorodiphenyldichloroethane and DDE, dichlorodiphenyldichloroethylene) can be found in wildlife; their endocrine disrupting actions have been extensively reviewed (Lundholm, 1987; Kelce *et al.*, 1995; Lundholm, 1997; Berg *et al.*, 2004; Holm *et al.*, 2006; Felton *et al.*, 2015). One metabolite (*o,p’*-DDT) causes malformations in the shell-producing gland while a different metabolite (*p-p’-*DDE) causes egg shell thinning by inhibiting prostaglandins, decreasing calcium uptake.

DDT bans in individual countries have been in place since the 1970’s (Bouwman *et al.*, 2013) and it was globally banned under the Stockholm Convention in 2004. Its use in vector control programmes is reviewed by van den Berg (2009).

Banning DDT lead to the population recovery of adversely affected birds of prey, for example, osprey (Henny *et al.*, 2010), bald eagles (Grier, 1982), peregrine falcon and sparrowhawks (Newton, 2013) and some populations of Californian condor (Felton *et al.*, 2015). Roos *et al.* (2012) reported on improvements in reproductive success that occurred concurrent with declines of DDT and PCBs in European otters, grey seals and sea eagles in Sweden.

Glaciers as a source of DDT in Adélie penguin eggs and subcutaneous fat is discussed by Geisz *et al.* (2008).

- - 1. For an overview on the work on alligators at Lake Apopka, see Guillette *et al.* (2000) and Woodward *et al.* (2011). A wide-range of adverse effects have been observed in Lake Apopka alligators (Guillette *et al.*, 2000; Toft *et al.*, 2003; Gunderson *et al.*, 2004; Milnes *et al.*, 2005; Milnes *et al.*, 2008; Milnes & Guillette, 2008).

- 1. Koppe and Keys (2002) discusses the history of discovery of effects and the regulatory history of polychlorinated biphenyls (PCBs); they were banned under the Stockholm Convention in 2002. Harrad *et al.* (1994) outlined the use and fate of PCBs in the UK.

PCBs have been found in wildlife since the late 1960’s (Risebrough *et al.*, 1968; Jensen, 1972). Examples of taxa in which PCBs have been found are listed in ¶AB21.

Different types (congeners) of PCBs may have either oestrogenic or anti-oestrogenic properties (Zhang *et al.*, 2014). Boas *et al.* (2006) reviewed the impact of PCBs on thyroid function; for mechanisms also see Das *et al.* (2006). PCBs also affect the stress hormone (corticosterone) system (e.g. Glennemeier and Denver (2001), Nordstad *et al.* (2012), Sonne (2010), Tartu *et al.* (2015b)).

PCBs are the major POP detected in wildlife in industrial regions (Huber *et al.*, 2015; Tartu *et al.*, 2015b) and are still having severe effects because of their toxicity, lipophilic nature and long environmental half-lives (Jepson *et al.*, 2016; Jepson & Law, 2016).

The replacement of PCBs as flame retardants with PBDEs is discussed by Vandenberg *et al.* (2015), Alaee *et al.* (2003) and Boas *et al.* (2006).

Reproductive failure in farmed mink fed contaminated salmon from the Great Lakes was observed in the late 1960’s; experiments confirmed PCBs to be the causal agent (Aulerich *et al.*, 1971; Aulerich & Ringer, 1977; Brunström *et al.*, 2001). Some mink populations are in decline (Wren, 1991; Basu *et al.*, 2007), show adverse health effects linked to PCBs (Harding *et al.*, 1999; Beckett *et al.*, 2005) and/or have body burdens high enough to have reproductive effects (Bursian *et al.*, 2006; Bursian *et al.*, 2013). Potential population level effects have also been observed in the related European mink (Lopezmartin *et al.*, 1994).

- - 1. Cetaceans may be particularly susceptible to the effects of POPs (McKinney *et al.*, 2011a; Sonne *et al.*, 2018). Jepson *et al.* (2016) reported on the high concentrations of PCBs in orca (killer whales, *Orcinus orca)*, striped dolphins (*Stenella coeruleoalba*) and harbour porpoises (*Phocoena phocoena*) in European waters. They suggest that these concentrations may be responsible for the continued decline of these populations; alternative causes of decline (including other pollutants) were ruled out. Other cetacean populations for which EDCs are of notable concern include beluga whales from the St Lawrence estuary (Québec, Canada) (Martineau *et al.*, 1987; Béland *et al.*, 1993) and orca on the coasts of British Columbia, Canada and Washington, USA (Ross *et al.*, 2000). Toxicity threshold is calculated from the toxic equivalency factors that allow for mixture of dioxins, furans and/or PCBs to be expressed as a single number, for details of their calculation, see Van den Berg *et al.* (1998).

Desforges *et al.* (2018) created an individual-based model for orca populations globally and collated data on PCB concentrations of the different populations. They calculated the effect of PCBs on reproduction and immune function for each of the 19 population and determined that the populations were predicted to decline for eight of these populations were predicted to decline (λ < 1). Stability was predicted for two populations; some model simulations suggest that these may be at risk of decline. Population increase was predicted for a further nine populations (λ > 1).

1. Johnson *et al.* (2013) described levels of oestrogens in European rivers; their potency is discussed by Sumpter and Johnson (2005). Khan and Nicell (2014) model how changes to contraceptive use might alter the level of oestrogens entering the environment.
   1. The American English names for these compounds are also commonly used – estrone, estradiol, estriol and ethinylestradiol. Sumpter and Johnson (2008), Tyler and Filby (2011), Jobling and Owen (2013) and Jobling (2014) provide historic accounts. The first surveys of oestrogenic effects of sewage effluent were conducted by Purdom *et al.* (1994), further UK surveys include Harries *et al.* (1997), Jobling *et al.* (1998); Jobling *et al.* (2002a); Jobling *et al.* (2002b); Jobling *et al.* (2006) and van Aerle *et al.* (2001).
   2. The phenomenon of feminisation in wild fish is reviewed by Bahamonde *et al.* (2013). Jobling and colleagues have conducted surveys that correlated wastewater treatment plants, in particular the levels of oestrogens released, with levels of intersex in roach throughout the United Kingdom (Jobling *et al.*, 1998; Jobling *et al.*, 2002a; Jobling *et al.*, 2002b; Jobling *et al.*, 2006). Jobling *et al.* (2002b) and Harris *et al.* (2011) demonstrated that higher levels of intersex are associated with a reduction in fertility and reproductive success in the roach. Fish populations have been found to be self-sustaining (Hamilton *et al.*, 2014; Johnson & Chen, 2017). Johnson and Chen (2017) did not find consistent correlations between predicted oestrogen concentrations and the abundance of four species of UK river fish (roach, perch, dace and bleak) in four river basins (38 sites) over six – 17 years.
      1. There were no differences in effective population size of roach in rivers with and without wastewater (Hamilton *et al.*, 2014). The effective population size is the number of reproducing individuals in a population.
   3. Oestrogens from livestock in the UK are discussed by Johnson *et al.* (2006); Matthiessen *et al.* (2006). Alvarez *et al.* (2013), Schoenborn *et al.* (2015) and Tremblay *et al.* (2018) outline the amounts entering the environment in the US, Switzerland and New Zealand respectively.

1. Runnalls *et al.* (2010) reports use of some endocrine-active pharmaceuticals (steroids) in the UK and discusses potential environmental impact. der Beek *et al.* (2016) reviews global presence of pharmaceuticals in the environment.
   1. Metformin is commonly prescribed for diabetes (World Health Organization, 2016; NHS choices). It can be found at high concentrations in the environment due to the large number of users (Anderson *et al.*, 2004; Scheurer *et al.*, 2012; Al-Odaini *et al.*, 2013; Oosterhuis *et al.*, 2013), high daily dose (Scheurer *et al.*, 2012; Oosterhuis *et al.*, 2013; British National Formulary, 2016) and because it is mainly excreted unchanged rather than metabolised (Scheurer *et al.*, 2012).

Studies on its environmental presence include Blair *et al.* (2013), Trautwein *et al.* (2014), also see Al-Odaini *et al.* (2013), Ghoshdastidar *et al.* (2015), Kosma *et al.* (2015) and ter Laak *et al.* (2014).

Mechanisms of action of metformin are described by Overturf *et al.* (2015) and Huang *et al.* (2016). As metformin does not structurally resemble oestrogens, it should not cause oestrogenic activity (Crago *et al.*, 2016); however, Niemuth and Klaper (2015) found it to have feminising effects on fathead minnow. For discussion of the need for validation of this result, see Sumpter *et al.* (2016) and reply (Klaper & Niemuth, 2016).

- 1. Use of fluoxetine (commonly known as Prozac) and other antidepressants is increasing (Mars *et al.*, 2017); Gardner *et al.* (2012) surveyed levels in wastewater effluent from throughout the UK. As it acts on the neuroendocrine system, adverse effects in wildlife are likely to be behavioural (Sumpter *et al.*, 2014). Mennigen *et al.* (2011) reviews experiments on small fish that have been conducted both at and above concentrations observed in the environment; while adverse effects have been observed at environmental levels (e.g. Margiotta-Casaluci *et al.* (2014), the evidence is insufficient to determine whether current levels of fluoxetine in rivers is of concern (Sumpter *et al.*, 2014). High levels of fluoxetine have been reported to alter *Daphnia magna* reproductive investment (Campos *et al.*, 2016), reduce feeding (and thus size) of amphibians (Conners *et al.*, 2009; Säfholm *et al.*, 2014) and alter diurnal rhythms of birds (Bean *et al.*, 2014).
  2. The anti-androgens, bicalutamide and cyproterone acetate, are the most commonly prescribed anti-androgens in the UK (Runnalls *et al.*, 2010). Modelling of concentrations in UK rivers by Green *et al.* (2015). Green *et al.* (2015) showed that there was no effect of mixtures of bicalutamide and cyproterone acetate on small fish (minnow & medaka) at the maximum concentrations predicted to occur in the UK in untreated effluent.
  3. For use, prescription rates and occurrence in the environment of progestins see Runnalls *et al.* (2010), Kumar *et al.* (2015) and Fent (2015). Kumar *et al.* (2015) and Orlando and Ellestad (2014) review effects of progestins on fish; different progestins vary greatly in potency. For studies on fish see Kumar *et al.* (2015), Paulos *et al.* (2010), Zeilinger *et al.* (2009), Runnalls *et al.* (2013), Fent (2015), and for amphibians, see Kumar *et al.* (2015) and Säfholm *et al.* (2014).

1. See reviews by Biswas *et al.* (2013) and Renner (2002). Low doses of antibiotics are also used as growth promoters in some countries (banned in the EU) and enter the environment (Page & Gautier, 2012) but as they do not act via the endocrine system, they are not EDCs.
   1. For uses of trenbolone in agriculture, see Reinhardt and Wagner (2014). It is banned in the EU (European Commission, 2017b) but is used illegally in bodybuilding e.g. Friedman *et al.* (2016). It enters freshwaters through livestock urine and manure (Saaristo *et al.*, 2013). Experimental studies in the laboratory (both at current environmental and higher than environmental levels) have demonstrated adverse effects on fish mating behaviour (e.g. Bertram *et al.* (2015), Heintz *et al.* (2015), Tomkins *et al.* (2016)) and sexual development both in frogs (Li *et al.*, 2015; Haselman *et al.*, 2016) and fish (Baumann *et al.*, 2014; Forsgren *et al.*, 2014; Leet *et al.*, 2015; Massart *et al.*, 2015).
2. See individual chemicals below for discussion about uncertainty surrounding harm and Vandenberg *et al.* (2015), Section 6 for a discussion about substitute chemicals.
   1. The uses of bisphenol A are described by Plastics Europe (2016) and Vandenberg *et al.* (2009) describes some of the biochemical properties of BPA. The movement of BPA into the environment is reported by Im and Loffler (2016) and Flint *et al.* (2012) while studies on the levels of BPA in freshwater include Fromme *et al.* (2002), Campbell *et al.* (2006), Flint *et al.* (2012), Scott *et al.* (2014) and Bhandari *et al.* (2015b).

The mechanisms of BPA are described in Vandenberg *et al.* (2012) and potency is discussed by Rubin (2011) (Section 6); Richter *et al.* (2007) and Segner *et al.* (2003) compare potencies in different tests. Moreman *et al.* (2018) demonstrates that the degradation product of BPA, MBP, is more oestrogenic than the parent compound.

Studies reviewing the effects of BPA on wildlife include Bhandari *et al.* (2015b), Oehlmann *et al.* (2009), Flint *et al.* (2012) and Mills and Chichester (2005). Intergenerational effects were observed at higher than environmental levels by Bhandari *et al.* (2015a); earlier studies are reviewed by Flint *et al.* (2012). Human health effects of BPA are controversial (Vandenberg *et al.*, 2009; Gies & Soto, 2013; Ranciere *et al.*, 2015). The details of BPA regulations can be found in Plastics Europe (2016), EFSA (2015) and Flint *et al.* (2012).

- 1. For uses of phthalates, see CDC (2016). The quantity of phthalates in the aquatic environment is reviewed by Tyler *et al.* (1998), Bhatia *et al.* (2015) and Gao and Wen (2016). Details on legislation can be found in European Council for Plasticisers and Intermediates (2014) and European Chemicals Agency (2015). Bioaccumulation and bioconcentration of phthalates tends to be low although it varies between compounds (Oehlmann *et al.*, 2009) and with species (Rhind *et al.*, 2005; Lenoir *et al.*, 2014; Adeogun *et al.*, 2015). Oehlmann *et al.* (2009) reviewed impact of phthalates on wildlife. More recent experiments conducted at levels higher than observed in the environment have found both feminisation and masculinisation of fish (Aoki *et al.*, 2011; Xu *et al.*, 2014; Bhatia *et al.*, 2015) and adverse effects on amphibian thyroid function (Shen *et al.*, 2011; Mathieu-Denoncourt *et al.*, 2015). Hauser and Calafat (2005) gives evidence for effects in humans.
  2. For details of polyfluoroalkyl and perfluoroalkyl substances (PFASs) chemistry, see Buck *et al.* (2011). There are a range of industrial uses of PFASs (Prevedouros *et al.*, 2006; Lindstrom *et al.*, 2011; Land *et al.*, 2015); airport use of PFAS-containing aqueous fire-fighting foams has severely contaminated waterways near airports (Awad *et al.*, 2011; Ahrens *et al.*, 2015).

One PFAS, perfluorooctanoic acid (PFOA, a surfactant) has been found in human serum (Hansen *et al.*, 2001; Lindstrom *et al.*, 2011; Land *et al.*, 2015) and later in wildlife where it bioaccumulates (Giesy & Kannan, 2001 ; Houde *et al.*, 2006; Lau *et al.*, 2007; Fair *et al.*, 2010); Houde *et al.* (2011); (Tartu *et al.*, 2018) PFASs are highly persistent due to the presence of the C-F bond.

For reviews of toxic and endocrine effects of PFASs in humans and other animals, see Lau *et al.* (2007), Jensen and Leffers (2008) and Post *et al.* (2012). Experimental findings of effects on the thyroid hormone system have been inconsistent (Chang *et al.*, 2008; Cheng *et al.*, 2011). While PFASs may alter sex hormone function (Du *et al.*, 2009; Shi *et al.*, 2009; Mommaerts *et al.*, 2011), many of these experiments were conducted at levels higher than those observed in the environment. Exposure to PFASs may be correlated with adverse reproductive effects in European eels (Couderc *et al.*, 2016), black-legged kittiwakes (Tartu *et al.*, 2014a) and tree swallows (Custer *et al.*, 2012). Part of the reason for the lack of evidence for harm is because there are technical challenges to working with PFASs (authors’ opinion). They are highly hydrophobic (i.e. repel water), as such, achieving and maintaining a given exposure level is difficult.

- 1. PBDE chemistry and use reviewed in Alaee *et al.* (2003) and Jinhui *et al.* (2017) respectively. It should be noted that levels of PBDEs reported from different studies may not be directly comparable as analytical methods and types assessed will differ in different laboratories (Frouin *et al.*, 2011).

Anti-androgenic and/or oestrogenic effects of PBDEs have been found in experiments conducted at levels similar to or higher than those observed in the environment (Fernie *et al.*, 2008; Eng *et al.*, 2012; Van Schmidt *et al.*, 2012; Neuman-Lee *et al.*, 2015; Yu *et al.*, 2015).

There are structural similarities between PBDEs and the thyroid hormone, thyroxine (T4) (Hale *et al.*, 2008); effects on thyroid function are reviewed by Yu *et al.* (2015). Studies have found correlations between levels of PBDEs and thyroid hormone levels in a range of wildlife species and populations (Hall *et al.*, 2003; Das *et al.*, 2006; Hall & Thomas, 2007; Villanger *et al.*, 2011a; Morrissey *et al.*, 2014). In experiments conducted at higher than environmental levels, PBDEs inhibited amphibian metamorphosis (Balch *et al.*, 2006) and altered expression of genes related to thyroid hormone production (Yost *et al.*, 2016). Human health effects are described by Law *et al.* (2014).

PBDES are widely present in the environment (Allchin *et al.*, 1999; Hale *et al.*, 2008; Bartrons *et al.*, 2012; Lee & Kim, 2015) and in wildlife, reviewed by Law *et al.* (2014) and Lee and Kim (2015). Allchin *et al.* (1999) documented release into UK rivers.

PBDEs bioaccumulate/bioconcentrate (Quinete *et al.*, 2011; Gaylor *et al.*, 2012; Mo *et al.*, 2012; Lee & Kim, 2015) and are maternally transferred (Wu *et al.*, 2009; Alava *et al.*, 2012; Law *et al.*, 2014).

For details of PBDE legislation see Jinhui *et al.* (2017) and Section (E).

- 1. For a review of the chemistry, sources and uses of perchlorate see Trumpolt *et al.* (2005). There are multiple forms of perchlorate, most commercial use involves one of ammonium perchlorate (NH_4_ClO_4_), perchloric acid (HClO_4_), potassium perchlorate (KClO_4_) or sodium perchlorate (NaClO_4_). Urbansky *et al.* (2001) reviews occurrence in mineral deposits used as fertiliser. Perchlorate is highly soluble in water and is a major groundwater contaminant in the USA (Hatzinger, 2005) causing human health concerns (Blount *et al.*, 2006). Levels of perchlorate in freshwater and drinking waters in the USA are reviewed by Dean *et al.* (2004) and Brandhuber *et al.* (2009).

The impact of perchlorate on thyroid hormone production is well understood (Wolff, 1998; Clewell *et al.*, 2004). Retardation of amphibian metamorphosis, thyroid gland abnormalities and reduced growth rates in amphibians have been observed in experiments at levels similar those observed at contaminated sites (Goleman *et al.*, 2002; Tietge *et al.*, 2005; Bulaeva *et al.*, 2015). Adverse effects on thyroid function have also been experimentally found in birds (McNabb *et al.*, 2004; Rainwater *et al.*, 2008), fish (Park *et al.*, 2006; Mukhi & Patino, 2007; Furin *et al.*, 2015), turtles (Eisenreich *et al.*, 2012). Thyroid disruption of fish and frogs has been correlated with environmental levels of perchlorate (Theodorakis *et al.*, 2006).

- 1. Chemistry, use and legislation of the alkylphenols, see Soares *et al.* (2008). Sumpter (2009) discusses the range of nonylphenol isomers. Nonylphenol binds to the oestrogen receptor (White *et al.*, 1994). For feminisation of fish in UK rivers, see Sheahan *et al.* (2002b); Sheahan *et al.* (2002a), for lab studies see Watanabe *et al.* (2017) and Pickford *et al.* (2003). Soares *et al.* (2008) and Berge *et al.* (2012) review presence in the environment globally. After the EU ban, its presence in imported textiles was still a major source (Månsson *et al.*, 2008); the importation of textiles containing alkylphenols has now been banned (Government Chemist and Environment Agency, 2015). The US EPA states that nonylphenol should not exceed 6.6 µg L^-1^ in freshwater and 1.7 µg L^-1^ in saltwater (David *et al.*, 2009).

1. If a chemical is identified as an EDC, the policy options open are different to if it is classed as a different type of pollutant (Thornton, 2007). Hill’s criteria (Fedak *et al.*, 2015) is one method that may be used to determine whether a chemical might be considered an EDC, but see Bergman *et al.* (2015).
   1. Diclofenac is a popular non-steroidal anti-inflammatory drug used to treat mild to moderate pain (McGettigan & Henry, 2013). The decline of *Gyps* vultures in India, Pakistan and Nepal and link with diclofenac has been well documented, reviewed by Pain *et al.* (2008), Cuthbert *et al.* (2014). Some other species have also suffered less-severe population declines as a result of diclofenac use (Cuthbert *et al.*, 2006; Galligan *et al.*, 2014; Sharma *et al.*, 2014). Mechanisms of action are primarily non-EDC and are described by Swan *et al.* (2006b) and Naidoo and Swan (2009).

Diclofenac has been banned for veterinary use (Pain *et al.*, 2008; Cuthbert *et al.*, 2014), while an alternative (meloxicam) is available (Swan *et al.*, 2006a; Naidoo *et al.*, 2010; Cuthbert *et al.*, 2014), illegal use occurs ([Cuthbert et al. 2016](#_ENREF_52)).

- 1. Solomon *et al.* (1996) detailed use of atrazine in corn production, its movement and persistence in the environment. For status in Europe and history of atrazine regulation see Lewis *et al.* (2015), European Commission (2003) and Ackerman (2007). Sass and Colangelo (2006) and Ackerman (2007) compare the US and EU approach to pesticide registration policy (in particular, how scientific evidence is used in decision-making) and how that has led to the different status for atrazine.

The mechanism behind (potentially) feminising effects is poorly understood (Papoulias *et al.*, 2014). Studies have found negative effects at (high) environmentally relevant levels using both the frog species *Xenopus laevis* (Hayes *et al.*, 2002b; Tavera-Mendoza *et al.*, 2002; Hayes *et al.*, 2006; Hayes *et al.*, 2010) and *Rana pipiens* (Hayes *et al.*, 2002a, 2003; Langlois *et al.*, 2010). Other studies have found no change to the measured endpoints in *Xenopus laevis* (Jooste *et al.*, 2005b; Oka *et al.*, 2008; Kloas *et al.*, 2009). The experimental methods used are frequently contested, for example, the study of Jooste *et al.* (2005b) was heavily criticised by Hayes (2005) and response (Jooste *et al.*, 2005a). Systematic reviews have also reached opposing conclusions (Rohr & McCoy, 2010; Van Der Kraak *et al.*, 2014). Langlois *et al.* (2010) also found some effects on metamorphosis.

Hayes (2004) criticised studies that have found no ED effects of atrazine on methodological grounds, while Van Der Kraak *et al.* (2014) has done the same for studies that found effects. A discussion of the association between funding source and findings is presented by Hayes (2004) and Reeves (2015). The debate over atrazine and its effects on amphibians has become highly politicised; see Dalton (2010), Howard (2013), Aviv (2014) and Reeves (2015).

## (C) How EDCs enter and persist in the environment

1. Point source pollution is defined as “pollution arising from specific identifiable points, such as the end of pipes discharging waste water” (Defra, 2012). The major point source is wastewater effluent (Keller *et al.*, 2014). Other sources include effluent from hospitals (Langford & Thomas, 2009; Verlicchi *et al.*, 2012) and industry (Allchin *et al.*, 1999; Pothitou & Voutsa, 2008). Industrial and military sites are often point sources of pollutants due to accidental or inappropriate waste disposal (Ormerod *et al.*, 2000; Henning *et al.*, 2003; Neigh *et al.*, 2006; Johnson *et al.*, 2009b; Fu *et al.*, 2011). Teuten *et al.* (2009) reviewed movement of plasticisers and other EDCs from waste disposal sites into surface waters. Diffuse pollution is defined as “pollution not arising from a specific, identifiable point”, such as from agricultural land and brownfield sites (Defra, 2012). Oestrogens produced by livestock runoff farmland and enter water courses exposing aquatic species (Johnson *et al.*, 2006; Matthiessen *et al.*, 2006). Terrestrial species may be affected by feeding on contaminated aquatic prey (Dods *et al.*, 2005; Markman *et al.*, 2008; Markman *et al.*, 2011) or by feeding on pastures fertilised with sewage sludge (e.g. (Rhind *et al.*, 2005; Rhind, 2009). Smith (2009) and Verlicchi and Zambello (2015) review the presence and implications of contaminants including EDCs in sewage sludge used as fertiliser. Approximately 80% of the UK’s sewage sludge is reused as a soil enhancer and fertiliser (this is the environmentally preferred option), up from 44% in 1992 (Defra, 2012). Sludge can contain household chemicals such as flame retardant PBDEs (Davis *et al.*, 2012).
   1. Reviews and examples of EDCs in wastewater include Campbell *et al.* (2006), Kasprzyk-Hordern *et al.* (2008), Miege *et al.* (2009), Arukwe *et al.* (2012), Stuart *et al.* (2012), Scott *et al.* (2014), Bhandari *et al.* (2015b), Sorensen *et al.* (2015).
   2. Import of hazardous material including waste of electrical and electronic equipment (e-waste) and release into the environment in Nigeria and China is described by Sindiku *et al.* (2015) and Wong *et al.* (2007) respectively. Reports of the presence of EDCs in wildlife at e-waste sites in China include Wu *et al.* (2009) and Fu *et al.* (2011). Routes of exposure and human health impacts of electronic waste are described by Frazzoli *et al.* (2010).
   3. The presence of androgenic and oestrogenic compounds in wood is reviewed by van den Heuvel (2010), dioxins were often present in pulp mill effluents (Kovacs *et al.*, 1995). Observational and experimental studies on the masculinisation of fish living downstream of pulp mill effluent include Larsson and Forlin (2002), Kovacs *et al.* (1995), Jones and Reynolds (1997) and are reviewed by van den Heuvel (2010). Human health effects of phyto-oestrogens are reviewed by Rietjens *et al.* (2017).

1. Jobling and Tyler (2003), Sumpter and Johnson (2005), Walsh *et al.* (2016) and Williams *et al.* (2009) review dilution in UK rivers. Johnson *et al.* (2013) and Keller *et al.* (2014) review dilution factors for Europe and internationally respectively. Comparisons between the UK, Europe and North America can be found in Tyler and Jobling (2008). Keller *et al.* (2014) discuss spatial and temporal variability of dilution factors within countries – reasons for differences are both due to seasonal flow variations and population patterns. Hall and Thomas (2007) compare PCB load in seals from different sites across the UK. Trautwein *et al.* (2014) showed how metformin and its transformation product, guanylurea, dilute as they travel downstream and enter the ocean. Dilution in the ocean was not sufficient to prevent adverse effects of TBT (Hallers-Tjabbes *et al.*, 1994).

1. Gusev *et al.* (2012) reviews intercontinental transport of persistent organic pollutants. Using knowledge of long-range atmospheric transport and properties of the compounds of interest, models have been constructed to determine the rate and mechanism by which chemicals might move through the environment (Prevedouros *et al.*, 2004; Fenner *et al.*, 2005; Guglielmo *et al.*, 2009). Studies describing movement of particular EDCs include work on DDT (Wania & Mackay, 1996), PCBs (Beyer & Biziuk, 2009) and PFASs (Prevedouros *et al.*, 2006; Kwok *et al.*, 2013).
   1. Reviewed by Nadal *et al.* (2015). Atmospheric transport is believed to be the dominant pathway of persistent organic pollutants to Antarctica (Noel *et al.*, 2009); much of the PCBs released in the UK is believed to have been transported to the Arctic (Harrad *et al.*, 1994).
2. Oestrogens and some pharmaceuticals may be broken down by photodegradation and/or microbial degradation (Lin & Reinhard, 2005; Zuo *et al.*, 2006; Cajthaml *et al.*, 2009; Yu *et al.*, 2013; Zuo *et al.*, 2013). For details of degradation in English rivers, see Jürgens *et al.* (2002).

Half-lives of persistent organic pollutants depend on both the compound (and for PCBs, type) and substrate in which the chemical occurs (de Mora *et al.*, 1995; Sarradin *et al.*, 1995; Sinkkonen & Paasivirta, 2000; Ritter *et al.*, 2011). Estimates of half-lives may be derived from laboratory experiments (Watanabe *et al.*, 1995; Dowson *et al.*, 1996).

- 1. Sumpter and Johnson (2008) discuss “pseudopersistence”. Examples of pseudopersistent EDCs in wastewater include BPA and high-use pharmaceuticals such as EE2, metformin or the antiandrogens (Daughton & Ternes, 1999; Zhang & Li, 2011; Hampel *et al.*, 2015).
  2. Pollutants may be contained within substrates such as harbour sediments, soils and glaciers. When these substrates are disturbed (or melt) the pollutant is released into the environment. These contaminated substrates are referred to as secondary sources. They will become relatively more important for persistent organic pollutants as primary sources (e.g. PCB-containing electrical capacitors) are eliminated (Nizzetto *et al.*, 2010; Stuart-Smith & Jepson, 2017); for example, sediments in harbours are a source of TBT (Antizar-Ladislao, 2008) and soil is likely to become a relatively more important source of PCBs in the UK (Lu *et al.*, 2015). Glaciers as a secondary source of POPs is reported by Weinhold (2009), Geisz *et al.* (2008), Cheng *et al.* (2014) and Kwok *et al.* (2013). PCBs have been reported from the deep ocean (Mariana and Kermadec trenches) by Jamieson *et al.* (2017).
  3. For example, PCBs are present in many buildings constructed between 1950-1970, in particular in caulk or sealant (Harrad *et al.*, 2009; Frederiksen *et al.*, 2012; Herrick *et al.*, 2016). PBDE flame retardants are present in a wide range of consumer products (Jinhui *et al.*, 2017).

1. Bioconcentration is the process by which a chemical is absorbed by an organism only through its respiratory and dermal surfaces, while bioaccumulation is the process in which a chemical substance is absorbed by all routes of exposure (dietary as well as dermal and respiratory paths) and thus includes food chain transfer. Chemicals that bioaccumulate or bioconcentrate are not expelled, as such, the amount increases in the animal’s body over time and therefore the internal concentration of the chemical will become greater than in the external environment. Biomagnification is when the concentration of the chemical in an organism exceeds that of its diet – i.e. levels of the compound increase up the food chain. These terms are defined in the ¶ Glossary and are reviewed and discussed by Arnot and Gobas (2006) and Yarsan and Yipe (2013). For example, the PFOA (a PFAS) concentration in the liver of beluga whales was 889 times that of cod (Houde *et al.*, 2006). Pollutants that biomagnify include PBDEs (Quinete *et al.*, 2011; Mo *et al.*, 2012; Lee & Kim, 2015), PCBs (Dietz *et al.*, 2000; Mackintosh *et al.*, 2004; Quinete *et al.*, 2011), DDT and other OC pesticides (Dietz *et al.*, 2000; Skarphedinsdottir *et al.*, 2010; Gui *et al.*, 2014), PFASs (Houde *et al.*, 2006; Haukas *et al.*, 2007; Kelly *et al.*, 2009).
   1. There was a positive correlation between age and PCB load in Svalbard ringed seals (Wolkers *et al.*, 1998) and Norwegian male coastal otters (Christensen *et al.*, 2010), male beluga whales (Wade *et al.*, 1997), male mink (Persson *et al.*, 2013) and in male and post-reproductive female northern resident orca (Ross *et al.*, 2000). Positive correlations have been found in some studies of polar bears (Smithwick *et al.*, 2005) but fasting may lead to negative correlations as fats containing EDCs are metabolised (Henriksen *et al.*, 2001) – when an animal is fasting (during times of stress or post-weaning) and living off their fat reserves, persistent organic pollutants will be mobilised along with lipids and concentrations of persistent organic pollutants in the serum will increase (Louis *et al.*, 2014; Jenssen *et al.*, 2015). Other reasons for an absence of a relationship can include maternal transfer (Wade *et al.*, 1997; Ross *et al.*, 2000; Bytingsvik *et al.*, 2012a), changes in elimination ability with age (Smithwick *et al.*, 2005) or “growth dilution” (Wang *et al.*, 2013), which is where the concentration of the chemical reduces as the animal increases in size (Arnot & Gobas, 2006); the absolute amount of chemical in the animal’s body may not change.

- 1. Leatherland (1997).
  2. Maternal transfer of PCBs in polar bears is discussed by Bytingsvik *et al.* (2012a) and Beckmen *et al.* (2003). Other compounds found in very young mammals includes PBDEs (Alava *et al.*, 2012), PFASs (Bytingsvik *et al.*, 2012b), phthalates have also been found in human breast milk (Adeogun *et al.*, 2015). By transferring POPs to their offspring, females lower their body burden of POPs. As a result, levels of POPs can be higher in males than females (e.g. Verreault *et al.* (2006), Bustnes *et al.* (2007), Wu *et al.* (2009), Christensen *et al.* (2010), Villanger *et al.* (2011b), Gui *et al.* (2014)).

## (D) How we know if an EDC is a problem in wildlife

1. There is an extensive literature on the human health effects of EDCs, see Bergman *et al.* (2012) and Damstra *et al.* (2002) for an overview. Most fish, amphibians, reptiles, birds and mammals respond the same way to most hormones, but invertebrates may not (Sumpter & Johnson, 2005; Vandenberg *et al.*, 2012). Sumpter and Johnson (2005) and Lyons (2003) discuss cross-species extrapolation of endocrine disrupting effects – termed the “read across” hypothesis. The read-across hypothesis is reviewed by Rand-Weaver *et al.* (2013), and Ankley and Gray (2013) explore the significance of it for toxicological testing. Brown *et al.* (2014) review the effects of human drugs on fish, for example, metformin alters glucose metabolism in fish (Hertz *et al.*, 1989; Polakof *et al.*, 2011; Capiotti *et al.*, 2014). Jobling and Tyler (2003) report on the sensitivity of fish to EDCs in comparison to other vertebrates.
   1. There may be differences in sensitivity both between species (e.g. Kidd *et al.* (2014), van Aerle *et al.* (2001)) and within species (e.g. Spearow (1999)). Hartung (2009) discusses some issues with the use of *read across* principles for establishing human health impacts. Sousa *et al.* (2014) and Sousa *et al.* (2013) reviewed the effects of TBT on a variety of taxa (bacteria, phytoplankton, plants, crustaceans, molluscs, fish and mammals).
   2. For example, humans are exposed to BPA through ingestion of food stored in BPA-containing plastics, and via thermal print paper and household plastic items (Rubin, 2011; Healy *et al.*, 2015), while exposure to fish and other aquatic wildlife is via wastewater and landfill leachate (Oehlmann *et al.*, 2009; Flint *et al.*, 2012).
2. Experiments can be technically challenging because of the very low concentrations of chemicals in wildlife. Examples of studies measuring changes in thyroid and sex hormone levels may be found in the sections describing individual compounds. The Organisation for Economic Co-operation and Development (OECD) provides experimental guidelines for *in vitro* and *in vivo* tests (Manibusan & Touart, 2017; OECD/OCDE, 2017) for various species of invertebrates (earthworms, insects and molluscs), fish, amphibians, birds and rodents. These guidelines are reviewed by Coady *et al.* (2017) and Manibusan and Touart (2017). A number of outcomes or endpoints may be assessed in these tests, reviewed by Ankley and Gray (2013). Endpoints may be referred to as *mechanistic* or *apical.* Mechanistic endpoints indicate how a chemical works, for example, an increase in vitellogenin production indicates that a feminising substance is oestrogenic rather than anti-androgenic. Changes in hormone levels are mechanistic endpoints. Apical endpoints are those associated with survival and reproduction (Marty *et al.*, 2017), these can be incorporated into models to assess effects on populations (Ankley & Gray, 2013). Dang *et al.* (2011) compares mechanistic (biomarker) and apical endpoints. The most commonly measured biomarker is vitellogenin, production in males indicates a chemical is oestrogenic (Sumpter & Jobling, 1995; Tyler *et al.*, 1996). Other *in vitro* assays commonly used include: Chemical Activated LUciferase gene eXpression (CALUX) used to detect specific chemicals, particularly dioxins (Windal *et al.*, 2005), yeast-based screens for detecting oestrogenic and androgenic substances (Sohoni & Sumpter, 1998; Bovee *et al.*, 2007), and the E-screen which is used to detect oestrogenic substances (Soto *et al.*, 1995).

Studies have explored the effects of development stage (Foran *et al.*, 2002; Liney *et al.*, 2005; Ciocan *et al.*, 2010; Crago *et al.*, 2016); sex (Villanger *et al.*, 2011b; Tartu *et al.*, 2014a); route or pathway of exposure (Pickford *et al.*, 2003) and duration of exposure (Vandenberg *et al.*, 2014) on responses to EDCs.

- 1. Rats and mice are often used to assess the human health impacts e.g. Chang *et al.* (2008), Jasarevic *et al.* (2011). Five species of small fish are frequently used to determine the effects of water-borne pollutants (fathead minnows *Pimephales promelas,* Japanese medaka *Oryzias latipes*, zebrafish *Danio rerio*, three-spined stickleback *Gasterosteus aculeatus* and rainbow trout *Oncorhynchus mykiss*), and the use of these species is often specified in OECD test protocols (OECD/OCDE, 2011; Manibusan & Touart, 2017). There may be differences in sensitivity both between species (e.g. Kidd *et al.* (2014), van Aerle *et al.* (2001)) and within species, e.g. Spearow (1999). Jaspers (2015) discusses some of the issues surrounding selecting an appropriate model species for experiments of ED effects, with particular reference to birds. For discussion of sample sizes needed, see Coady *et al.* (2017). Work on the development of a multi-generational avian test was abandoned in part due to the large number of animals that would be required to achieve statistical power (OECD/OCDE, 2018a). Multi-generational effects are reviewed by Parrott *et al.* (2017).

- 1. Lewis *et al.* (2002) describes how the NOAEL and related measures are determined. NOEL (no observable effect level) may also be used.

- 1. Chen *et al.* (2010) and Johnson (2010) investigated how river oestrogen concentrations changed both downstream of point-sources and between seasons in Taiwan and the UK respectively. Assessments of concentrations of oestrogens in rivers have been conducted for the UK (Williams *et al.*, 2009; Williams *et al.*, 2012) and Europe (Johnson *et al.*, 2013). Weltje and Sumpter (2017) provides a comment on literature reports of “environmentally relevant concentrations”.
  2. For overviews of non-monotonic effects see Lagarde *et al.* (2015), Zoeller and Vandenberg (2015), Futran Fuhrman *et al.* (2015) and Parrott *et al.* (2017). Vandenberg *et al.* (2012) provides tables (Table 6 & 7) listing cases of non-monotonic doses responses (NMDR); a wide variety of EDCs are implicated (but see Rhomberg and Goodman (2012) and reply by Vandenberg (2015)). Vandenberg (2015) and Vandenberg *et al.* (2012) describe potential mechanisms of non-monotonic effects, it is likely they occur due to multiple simultaneous modes of action producing counter-acting effects.

Parrott *et al.* (2017) discusses what NMDR might mean for risk assessment. NMDRs are often considered in conjunction with low-dose effects because often the change in directionality occurs at low doses (e.g. Futran Fuhrman *et al.* (2015)). However, it should be considered a separate issue as the change in directionality of the response can, in theory, occur at any concentration (Beausoleil *et al.*, 2013; Vandenberg, 2014).

- 1. Standardised ecotoxicity tests have been developed by the OECD (OECD/OCDE, 2017). For definitions of low dose effects, see Kortenkamp (2007) and Vandenberg (2014), and Zoeller *et al.* (2014) discusses issues surrounding these definitions. For a detailed discussion about how low dose effects can occur, the difficulty in extrapolating to or from the effects at higher doses and a table listing studies in which low dose effects have been found, see Vandenberg *et al.* (2012) and Vandenberg (2014). Kortenkamp (2008) reviews experimental studies of mixtures of EDCs at low-doses. Kaiser (2000) reports on the acceptance by the US EPA that low dose effects occur and need to be incorporated into testing, also see Beausoleil *et al.* (2013).
  2. For evidence of maternal transfer of EDCs in the eggs of birds or reptiles and in very young mammals where transfer occurs mostly via milk see Beckmen *et al.* (2003) and Stockin *et al.* (2010). EDCs found in eggs of fish, amphibians, birds and reptiles include PCBs (French Jr *et al.*, 2001; Henning *et al.*, 2003; Alava *et al.*, 2011a; Basile *et al.*, 2011; Erikstad *et al.*, 2013); DDT and other OC pesticides (Woodward *et al.*, 2011; Stoker *et al.*, 2013; Colabuono *et al.*, 2015); phthalates (Huber *et al.*, 2015); PBDEs (Wu *et al.*, 2009; Law *et al.*, 2014); PFASs (Yoo *et al.*, 2008; Leat *et al.*, 2013; Routti *et al.*, 2015), butyl tins (Hu *et al.*, 2009). For details of maternal transfer via milk, see ¶AB17.c.

Schwindt (2015) reviewed evidence for transgenerational effects following either maternal or paternal exposure to EDCs in aquatic wildlife (fish, amphibians and invertebrates). Koppe and Keys (2002) discusses how transgenerational effects may occur in relation to PCBs. The best documented example of transgenerational effects is that of diethylstilbestrol (DES), a non-steroidal oestrogen used as a human pharmaceutical. Observational studies in humans and experiments in rats have found similar adverse effects in children/rat pups exposed *in utero* and in their offspring (Fenichel *et al.*, 2015).

Laboratory studies that have noted greater effects of EE2 on the offspring of exposed fish (or later generations) than in the exposed fish themselves include Nash *et al.* (2004) and Bhandari *et al.* (2015a). However, mechanisms for these effects are unclear. Hamilton *et al.* (2015) did not find inter-generational effects in the roach.

- 1. Wastewater contains a mix of pharmaceuticals (Daughton & Ternes, 1999; Overturf *et al.*, 2015) and various other compounds (Kasprzyk-Hordern *et al.*, 2008; Stuart *et al.*, 2012; Sorensen *et al.*, 2015), notably phthalates (Bhatia *et al.*, 2015) and BPA and other phenolic compounds (Berge *et al.*, 2012; Zheng *et al.*, 2015). Animals living in the high Arctic generally contain a wide range of persistent organic pollutants (Bustnes *et al.*, 2007; Villanger *et al.*, 2011b; Villanger *et al.*, 2011a; Erikstad *et al.*, 2013; Routti *et al.*, 2014; Andersen *et al.*, 2015; Routti *et al.*, 2015). Kapraun *et al.* (2017) describes a technique for identifying common mixtures of chemicals for studies of human exposure. Relatively new non-target screening methods can determine which mixtures are commonly found in wildlife e.g. Norman Network (2012-2018).

Effects of mixtures of EDCs that have the same mode of action (e.g. mixtures of oestrogenic compounds) are reviewed by Kortenkamp (2007) and Kortenkamp (2008). Studies providing examples of additive effects include Brian *et al.* (2005), Miller *et al.* (2012), Rajapakse *et al.* (2002) and Thorpe *et al.* (2003). Where departures from additivity have been observed e.g. Crofton *et al.* (2005), the effects have been small (Kortenkamp, 2007).

The combined impact of EDCs that function differently (e.g. androgens with oestrogens) is poorly understood, however, their action cannot be assumed to be additive (Kortenkamp, 2007; Futran Fuhrman *et al.*, 2015). We also have little understandings how EDCs may interact with other non-EDC pollutants.

- - 1. Thrupp *et al.* (2018).
    2. Toxic equivalency factors (TEF) outlined by Van den Berg *et al.* (1998) and oestradiol equivalency factors by Williams *et al.* (2009). To calculate the EEQ, the potency of each compound is evaluated as a fraction of oestradiol; the final EEQ concentration is the sum of the quantities of different oestrogens corrected for their different potencies. The TEF uses a similar approach with 2,3,7,8-tetrachlorodibenzo-*p*-dioxin (TCDD) used as a standard.

1. Models that attempt to assess the impacts of EDCs on populations include Hanson *et al.* (2005), Hall *et al.* (2006), (Goutte *et al.*, 2014).
   1. For example, a minor change in timing of metamorphosis in amphibians due to thyroid disruption causes changes in adult body size (earlier metamorphosis leads to smaller adults). Timing of metamorphosis can also be affected by other stressors (Wilbur & Collins, 1973; Rohr *et al.*, 2004), and these effects are likely to be additive (Darling & Côte, 2008). Size then affects prey availability, predation risk and mating success (Rohr *et al.*, 2004; Rohr & McCoy, 2010).
   2. For example, UK river fish may spawn once in spring or multiple times from April to October (Nunn *et al.*, 2007). As concentrations of oestrogens in UK river water are typically highest in late summer (Johnson, 2010), exposure of the early life-history stages of river fish will vary depending on spawning date.
   3. Population-level processes are those processes whose magnitude depends on population size (density-dependent factors; a typical example would be starvation due to competition for food). The reduction in a species population size caused by EDCs (a density-independent factor) may be less if they result in reduced density-dependent mortality through factors acting subsequently. A reduction in birth rate might increase survivorship due to less competition for food. Such effects are likely to be common in wildlife population dynamics though very difficult to predict in the absence of detailed study. “Allee effects” are density-dependent factors that increase in severity at low densities (a typical example would be reduced fecundity due to failure to find a mate at low population densities). For further discussion, see Mace *et al.* (2008). An example of population modelling incorporating Allee effects is Molnar *et al.* (2014)’s model of the polar bears of Viscount Melville Sound, Canada.
2. Thousands of studies have measured the presence of persistent organic pollutants in wildlife. Evidence of endocrine disrupting chemicals have been found in wildlife far from pollution sources, for example, in the high Arctic (Routti *et al.*, 2014; Andersen *et al.*, 2015; Tartu *et al.*, 2018), Antarctica (George & Frear, 1966; Bustnes *et al.*, 2007; Routti *et al.*, 2015), in high alpine lakes (Bartrons *et al.*, 2012), in the deep ocean (Takahashi *et al.*, 2000), on isolated islands (Alava *et al.*, 2011b; Bachman *et al.*, 2014; Wang *et al.*, 2015).

POPs that are EDCs have been found in a biologically diverse range of taxa at all trophic levels. However, in many populations, concentrations will be low enough that they do not present harm to the organism. The results from experiments outlined in ¶AB7.c on mink have been used to determine toxic concentrations for other species such as otter (Brunström *et al.*, 2001) and various marine mammals (Folland *et al.*, 2016). The toxic equivalent threshold calculated for harbour seals was used to determine whether PCB levels in orca could be having adverse effects (Ross *et al.*, 2000). Some representative examples of POPs that are EDCs in wildlife include:

Invertebrates: apple snails (Fu *et al.*, 2011); crustaceans and bivalves (Lee & Kim, 2015) .

Freshwater fish: lake sturgeon (Jacobs *et al.*, 2014), white sturgeon (Feist *et al.*, 2005), fish in the Salton Sea, California (Moreau *et al.*, 2007), large-scale suckers (Jenkins *et al.*, 2014); mummichogs (Matta *et al.*, 2001), Elbe river fish (Cerveny *et al.*, 2016), largemouth bass (Colli-Dula *et al.*, 2016), European eels (Couderc *et al.*, 2016).

Marine fish: deep-sea lantern fish (Takahashi *et al.*, 2000), cod (Powley *et al.*, 2008), commercial fish species (Taylor & Johnson, 2016).

Amphibians and Reptiles: diamond backed terrapins (Basile *et al.*, 2011), cricket frogs (Reeder *et al.*, 1998); loggerhead turtles (Alava *et al.*, 2011a); alligators (Woodward *et al.*, 2011).

Birds: dippers (Ormerod *et al.*, 2000), American robins (Henning *et al.*, 2003), high Arctic eiders and shags (Huber *et al.*, 2015), Cooper’s hawks (Elliott *et al.*, 2015), chinstrap penguins (Jara-Carrasco *et al.*, 2015); albatrosses (Tartu *et al.*, 2015a; Wang *et al.*, 2015), common eider ducks (Tartu *et al.*, 2015a), south polar skua (Bustnes *et al.*, 2006), herring gulls (Fox *et al.*, 2008; Huber *et al.*, 2015; Letcher *et al.*, 2015), tree swallows (Custer *et al.*, 2014), lesser black-backed gulls (Bustnes *et al.*, 2008).

Land mammals: deer mice (Johnson *et al.*, 2009b), humans (Sousa *et al.*, 2013), otters (Basu & Head, 2010; Christensen *et al.*, 2010; Roos *et al.*, 2012), Arctic foxes (Aas *et al.*, 2014; Andersen *et al.*, 2015), mink (Hornshaw *et al.*, 1983), grizzly bears (Christensen *et al.*, 2005), badgers, raccoon dogs, wild boar and deer (Tomza-Marciniak *et al.*, 2014).

Sea mammals: polar bears (Tartu *et al.*, 2018); blue whale (Trumble *et al.*, 2013); harbour seals (Hall & Thomas, 2007); Californian sea lions (Le Boeuf *et al.*, 2002), various cetacean species (Law *et al.*, 2005; Bachman *et al.*, 2014; Ryan *et al.*, 2014), Hector’s and Maui’s dolphins (Stockin *et al.*, 2010); harbour porpoises (Beineke *et al.*, 2005); sea otters (Murata *et al.*, 2008), bottlenose dolphins (Lahvis *et al.*, 1995; Schwacke *et al.*, 2012; Adams *et al.*, 2014); ringed seals (Routti *et al.*, 2015; Gebbink *et al.*, 2016).

- 1. Darbre (2015) states that the presence of EDCs in the body is not an adverse effect and does not mean that adverse effects have occurred. However, if the EDCs were not present, there would be no concern. Diethylstilbestrol (DES) in humans is an example where adverse effects were observed long after the chemical had dissipated, exposure *in utero* caused vaginal cancer later in life (Fenichel *et al.*, 2015). Green *et al.* (2018) found that early life exposure of zebrafish to EE2 increased sensitivity to different oestrogens (EE2, BPA and phyto-oestrogens) later in life.

- 1. Sonne (2010) gives reasons why Arctic species are so affected by PCBs. Examples of predatory birds and high Arctic sea mammals containing high concentrations of persistent organic pollutants include:

Predatory birds: herring gulls (Huber *et al.*, 2015; Letcher *et al.*, 2015), glaucous gulls (Sagerup *et al.*, 2009; Erikstad *et al.*, 2013; Tartu *et al.*, 2015a); black legged kittiwake (Tartu *et al.*, 2014a), peregrine falcon (Holmstrom *et al.*, 2010; Elliott *et al.*, 2015); marsh harrier (Pain *et al.*, 1999); bald eagles (Grier, 1982; Bowerman *et al.*, 2003; Pittman *et al.*, 2015); northern fulmar (Verreault *et al.*, 2013); common kestrel (Eriksson *et al.*, 2016), tawny owl (Eriksson *et al.*, 2016), osprey (Eriksson *et al.*, 2016), barn owls (Jaspers *et al.*, 2013), great skua (Leat *et al.*, 2013), ivory gull (Lucia *et al.*, 2015).

High Arctic sea mammals: polar bears (Villanger *et al.*, 2011b), various seal species (Hall & Thomas, 2007; Routti *et al.*, 2008; Gabrielsen *et al.*, 2011; Routti *et al.*, 2015; Gebbink *et al.*, 2016), polar bears (Derocher *et al.*, 2003; Smithwick *et al.*, 2006; McKinney *et al.*, 2011b; Dietz *et al.*, 2013), beluga whales (McKinney *et al.*, 2006; Raach *et al.*, 2011), orca (Ross, 2006; Noel *et al.*, 2009; Jepson *et al.*, 2016). Orca are the most highly PCB-contaminated mammal both in Europe and globally (Wolkers *et al.*, 2007).

- 1. Examples of studies that have found high degrees of correlation between the different persistent organic pollutants, many of which are EDCs, include Alava *et al.* (2012), Bustnes *et al.* (2007), Das *et al.* (2006), Elliott *et al.* (2015), Pain *et al.* (1999) . For example, Erikstad *et al.* (2013) found that the correlation between the different types of PCBs and organochloride pesticides measured in glaucous gulls on Bear Island, Svalbard, Norway ranged from 66-98%.

1. Individual level effects are changes that alter the health of an individual animal. Lyons (2003) and Vandenberg (2015) highlight some of the difficulties with commonly measured endpoints. Hirabayashi and Inoue (2011) explain that an observed change to hormone levels may not always be considered an adverse effect due to homeostatis. There are a range of biomarkers available for various endocrine systems, see reviews by Scholz and Mayer (2008), Hutchinson *et al.* (2005). Other stressors (natural and anthropogenic) can also cause pathologies that appear similar to the effects of EDCs (e.g. Sonne *et al.* (2005)). Epidemiological criteria can be used to link stressors including EDCs with observed pathologies (Fox, 1991); for example, McMaster *et al.* (1996) used these criteria to link pulp mill effluent with fish reproductive abnormalities.
   1. The World Health Organization (1993) defines a biomarker as “any measurement that reflects an interaction between a biological system and an environmental agent, which may be chemical, physical or biological”. There are a range of biomarkers available for various endocrine systems, see reviews by Scholz and Mayer (2008), Hutchinson *et al.* (2005). Using meta-analysis, Bosker *et al.* (2010) determined that there was a significant relationship between biomarkers (sex hormones, VTG and relative gonad size) and reproductive output (egg production) in small fish but there was high uncertainty when predicting population-level effects from these biomarkers. They suggest that multiple biomarkers should be used due to the incidence of false negatives, however this may increase the rate of false positives.
      1. Vitellogenin (*vtg*) is an indicator of oestrogen exposure in egg-laying vertebrates (Robinson, 2008) but not invertebrates (Short *et al.*, 2014). Vitellogenin is often used as an indication of feminisation of fish (i.e. intersex) however the relationship between the two has not been well-characterised (Bahamonde *et al.*, 2013).
   2. Mills and Chichester (2005) review reproductive effects on small fish and Sonne (2010) reviews the reproductive impacts of EDCs on polar bears, sledge dogs and Arctic foxes. Compounds with adverse effects on reproduction include (but are not limited to) DDT, TBT, PCBs, trenbolone, steroid oestrogens, BPA, phthalates, nonyl-phenol.

EDCs that can override effects of temperature on sex determination in turtles and/or crocodilians causing altered sex ratios include dicofol, DDT, E2, BPA, chlordane, dioxins and PCBs (Crews *et al.*, 1995; Guillette *et al.*, 2000; Vos *et al.*, 2000; Milnes & Guillette, 2008; Bhandari *et al.*, 2015b; Jandegian *et al.*, 2015).

Behavioural effects (reviewed by Basu and Head (2010)) are likely to be underreported (Lyons, 2006).

- 1. Thyroid hormones are essential for normal development and for the control of many aspects of adult physiology in vertebrates (Weetman, 2010; Gore *et al.*, 2015). The major thyroid hormones are thyroxine (T4) and triiodothyronine (T3); both increases and decreases in levels of thyroid hormones are problematic. Compounds with adverse effects on thyroid hormone production include phthalates, PCBs, PBDE, perchlorate.

Rohr and McCoy (2010) in the section entitled “*Background on metamorphosis*” discuss how thyroid hormone disruptors can affect the timing of amphibian metamorphosis and the consequences of this. Changes in timing of metamorphosis can be responsible for changes to adult body size (Mathieu-Denoncourt *et al.*, 2015).

Bone density is influenced by both thyroid (Gorka *et al.*, 2013) and sex hormones (Cauley, 2015). Effects of EDCs on bone density are reviewed by Agas *et al.* (2013).

- 1. While EDCs can have effects on immune function, the effects are mostly weak and the mechanisms are poorly understood (Weybridge+15, 2012), reviewed by Milla *et al.* (2011). Derocher *et al.* (2003) has suggested that impaired immune system in Svalbard polar bears due to POPs might be limiting polar bear population growth. Links between the sex hormone system and immune system in rodents are reported by Grossman (1985). Adverse effects on the immune system have been observed with TBT in harbour seals (Frouin *et al.*, 2008) and reviewed by Fent (1996), PCBs in bottlenose dolphins (Schwacke *et al.*, 2012) and seals (Hammond *et al.*, 2005), trenbolone in rainbow trout (Massart *et al.*, 2015), PFOA in humans (Post *et al.*, 2012). EDCs can also affect the stress hormones (glucocorticoids) which regulate the immune system e.g. Tartu *et al.* (2014b).

1. Some of the difficulties in linking population declines with pollutants are highlighted by Safford and Jones’ (1997) investigation into the causes of Mauritius kestrel and cuckoo-strike declines, which they determine were caused by DDT. Roos *et al.* (2012) is an example of a correlative study linking improvements in reproductive success in European otters, grey seals and sea eagles in Sweden with declining concentrationss of persistent organic pollutants. Other environmental changes may obscure the association between population changes and pollutant concentrations. For example, Henny *et al.* (2010) discusses conservation measures and other factors that occurred contemporaneously with DDT decline that may have independently caused an increase in osprey numbers. Sumpter and Johnson (2008) discuss improvements in overall water quality (decreases in gross pollution) that could be obscuring any relationship between oestrogen concentrations and fish population size.
2. Authors’ opinion.

- 1. Kidd *et al.* (2007) added ethinyl oestradiol (EE2) to a lake in the Canadian Experimental Lakes Area in Ontario and monitored changes in the densities of different species compared to both reference lakes and the treated lake prior to the EE2 additions. Fathead minnow (*Pimephales promelas*) nearly became extinct (Kidd *et al.*, 2007) but the population recovered after the treatment ended (Blanchfield *et al.*, 2015). There were differences in the population responses of other fish and invertebrates (Palace *et al.*, 2006; Kidd *et al.*, 2014).

## (E) Major legislation concerning EDCs

1. An overview and history of the Convention can be found at Stockholm Convention (2008b) and Stockholm Convention (2008c), respectively.
   1. Most of the POPs are listed under Annex A. PBDEs have a specific exemption for use in recycled products. DDT and PFOS are listed under Annex B that states: “Parties must take measures to restrict the production and use of the chemicals listed under Annex B in light of any applicable acceptable purposes and/or specific exemptions listed in the Annex”. There are a number of accepted purposes for use of PFOS including for photoimaging, use in firefighting foam, in medical devices and in baits for leafcutter ants. The sole accepted purpose of DDT is “disease vector control” – i.e. control of malarial mosquitoes (Stockholm Convention, 2008d). The World Health Organization (2011) recommends that DDT should only be used for indoor spraying and use must be carefully monitored.
2. The selection of risk *vs.* hazard-based approaches to EDC regulation is discussed by Matthiessen *et al.* (2017). Details on the REACH legislation is provided by the European Chemicals Agency (2017) and how it relates to EDCs by the European Commission (2016c). Criteria under which chemicals are considered EDCs for regulatory purposes by the EU are outlined in European Commission (2016b), European Commission (2017a) and Bourguignon *et al.* (2016). For comment and response see Bourguignon *et al.* (2016), Watson (2016) and Kortenkamp *et al.* (2016). For details of the hazard-based approach in the Plant Protection Products Regulation and Biocidal Products Regulation see Slama *et al.* (2016). Restrictions on use of EDCs may be applied under REACH legislation in the form of “risk management measures” (Bruinen de Bruin *et al.*, 2007). Silbergeld *et al.* (2015) compare REACH legislation with legislation in other countries, particularly the USA and China. Also see European Chemicals Agency (2018). EDCs registered in the EU as plant protection products and associated “cut-off criteria” are reviewed by Marx-Stoelting *et al.* (2014) and consequences discussed at Chandler *et al.* (2008) and Theodoris (2008). “Cut-off criteria” would allow the use of EDCs in plant protection products where the risk of human exposure is negligible (Marx-Stoelting *et al.*, 2014).
   1. EU chemical and environmental legislation is supplemented by global and regional conventions to which the EU, Member States and other European countries are party, such as the Helsinki Convention on the Baltic Sea (1992), OSPAR Convention on the NE Atlantic (1992), Stockholm Convention on Persistent Organic Pollutants (2001) and Minamata Convention on Mercury (2013) (although it is not clear whether mercury is an EDC).
   2. Details of the EU Water Directives Framework described at European Commission (2016a). The oestrogens and diclofenac have been included on the First Watchlist of the Environmental Quality Standards Directive for the “specific purpose of better informing suitable risk reduction measures”. For further details and discussion on the Watchlist, see Negrão de Carvalho *et al.* (2015) and Owen and Jobling (2012). Potential of cosmetics to act as endocrine disruptors is reviewed by Nicolopoulou-Stamati *et al.* (2015). For information about the Marine Strategy Framework Directive of the EU aimed at achieving or maintaining *“Good Environmental Status”* in European seas, see Berg *et al.* (2015) and Lyons *et al.* (2017). *“Good Environmental Status”* is determined by 11 Descriptors; Descriptor 8 states “Concentrations of contaminants are at levels not giving rise to pollution effects”, for discussion of this Descriptor, see Lyons *et al.* (2017).

- 1. Kampa *et al.* (2010) and Keil (2010) review regulation relating to pharmaceuticals.
  2. Azoles are reviewed by Matthiessen and Weltje (2015). They can be EDCs but adverse effects only occur at levels higher than observed in the environment, e.g. Brown *et al.* (2015).

1. Details of the Basel Convention described in Basel Convention (2011) and SAICM at SAICM (2017). Breivik *et al.* (2011) discusses export of waste (some of which is illegal) from developed to developing countries.

## (F) What can be done about EDCs

1. Many POPs have been banned under the Stockholm Convention and/or EU Legislation. Severe restrictions may be placed on the use of particular substances under the Stockholm Convention or under REACH legislation in the form of risk management measures (Bruinen de Bruin *et al.*, 2007). The Stockholm Convention allows for malarial spraying for vector control purposes, i.e. control of mosquitoes that vector malaria. The WHO recommends indoor residual spraying of DDT in epidemic areas and in areas with high and constant malaria transmission; the benefits to human health of preventing malaria are thought to outweigh the adverse effects of DDT (Mandavilli, 2006; World Health Organization, 2011). Wildlife effects may not have been adequately studied (Bouwman *et al.*, 2011).
   1. While persistent organic pollutants are widely present in the environment even after banning, overall levels of banned chemicals have declined in the environment e.g. Koschorreck *et al.* (2015) and in wildlife, e.g. Dietz *et al.* (2013). There will be time lags between bans and declines (Holmstrom *et al.*, 2010).
   2. Examples of population recovery after bans are described above (TBT ¶AB7.a; DDT ¶AB7.b; PCBs ¶AB7.c)
2. Vandenberg *et al.* (2015) discusses “regrettable replacements”. Examples of replacements with non-EDC compounds include the replacement of DDT by other pesticides, primarily organophosphates and carbamates (Oberemok *et al.*, 2015) and replacements of TBT with copper-based biocides (Ciriminna *et al.*, 2015). However these compounds may have non-EDC adverse effects (Evans, 1999).
   1. PBDEs were used to replace flame-retardant PCBs (Alaee *et al.*, 2003; Boas *et al.*, 2006) and are released when PBDE-containing items are discarded or recycled (Wu *et al.*, 2009; Gaylor *et al.*, 2012). PBDEs have been replaced with various novel brominated flame retardants (Alaee *et al.*, 2003; Ali *et al.*, 2011; Dodson *et al.*, 2012) that may also have ED effects (Patisaul *et al.*, 2013) and have been found in Antarctic wildlife (Wolschke *et al.*, 2015).

Various bisphenol compounds (bisphenols AF, B, F and S) are popular replacements for BPA (Kinch *et al.*, 2015) and have similar or greater endocrine-disrupting (oestrogenic) effects (Yang *et al.*, 2011; Eladak *et al.*, 2015; Rochester & Bolden, 2015; Chen *et al.*, 2016; Usman & Ahmad, 2016), including in laboratory fish (Ji *et al.*, 2013; Naderi *et al.*, 2014; Qiu *et al.*, 2016; Le Fol *et al.*, 2017; Moreman *et al.*, 2017). Their potency compared to BPA is reviewed by Chen *et al.* (2016), Table 3. Moreman *et al.* (2017) states that the rank order of oestrogenicity in a range of zebrafish tissues was BPAF > BPA = BPF > BPS. Seltenrich (2015) discusses some of the issues surrounding finding replacement products for BPA and other plasticisers. Glass baby bottles are the fastest growing segment of the baby bottle market due to concern over EDCs in plasticisers, discussed in (Business Wire, 2016).

1. An overview of wastewater treatment in the UK can be found at Defra (2012). The size (number of people served), type and flow of the sewage treatment works has a major bearing on the amount of oestrogenic chemicals in the environment (Sumpter & Johnson, 2005). Different drugs are removed to different extents by sewage treatment plants (Wennmalm & Gunnarsson, 2009) and different types of sewage treatment have differing abilities to remove oestrogenic chemicals (Baynes *et al.*, 2012). Gardner *et al.* (2013) and (Comber *et al.*, 2018) review the effectiveness of UK wastewater treatment plants on their ability to remove pharmaceuticals (and other chemicals) from wastewater. Oller *et al.* (2011) and Ruhl *et al.* (2014) review the ability of advanced oxidation processes (i.e. ozonation and other technologies) and activated carbon to remove various compounds respectively. A disadvantage of granulated activated carbon is that it moves the compound from the wastewater into the carbon (reviewed by Zanella *et al.* (2014)). Ternes *et al.* (2004) discusses the chemistry of different compounds (not all EDCs) and how that affects how they move through different types of sewage treatment works, also see Miege *et al.* (2009). The ability of constructed wetlands to remove pharmaceuticals is reviewed by Li *et al.* (2014) and Morvannou *et al.* (2015). Johnson *et al.* (2007) models how different wastewater treatments could alter the concentrations of oestrogens in river water. A reduction in oestrogenicity should reduce levels of intersex. In Kitchener, ON, Canada, the wastewater treatment plant was upgraded from carbonaceous activated sludge to nitrifying activated sludge (Hicks *et al.*, 2017). This was associated with a decrease in intersex in male rainbow darter. Prior to the upgrade 70-100% of male fish were intersex, this decreased to <10% subsequently and the severity of intersex was also reduced.
   1. Costs calculated by Owen and Jobling (2012). Other options discussed by Baynes *et al.* (2012) and (Margot *et al.*, 2013).
   2. Authors’ opinion.
2. UNEP Chemicals and Waste Branch (2016) states that less than 20% of the total PCBs produced have been eliminated. The largest environmental source is leakage from electrical equipment (transformers and capacitors) ((Creaser *et al.*, 2007; UNEP Chemicals and Waste Branch, 2016)), they are also present in caulk or sealant of many buildings built 1950-1970, reviewed by (Harrad *et al.*, 2009; Frederiksen *et al.*, 2012; Herrick *et al.*, 2016). See discussion by Stuart-Smith and Jepson (2017) and Law and Jepson (2017).

PBDEs enter the environment when household products containing them are discarded (Rahman *et al.*, 2001; Balch *et al.*, 2006; Gaylor *et al.*, 2012; Neuman-Lee *et al.*, 2015).

UK regulations require that persistent organic pollutants are either destroyed by incineration or chemical destruction or are permanently stored underground (UK Government, 2015). For a review of techniques for persistent organic pollutant destruction, see Amend and Lederman (1992) and Magr (2003). New dechlorination (destruction of the molecule through removal of the chlorine atoms) technologies are being developed e.g. Celtic Recycling (2009).

- 1. Challenges of dealing with persistent organic pollutants at waste management sites is reviewed by Weber *et al.* (2011) and Die *et al.* (2015). Examples of EDCs presence in landfills and recycling facilities can be found in Allchin *et al.* (1999); Wu *et al.* (2009); Fu *et al.* (2011)
  2. See above. The presence of PBDEs affects the recycling of this e-waste (Li *et al.*, 2013).

- 1. Guerrero *et al.* (2013) reviews issues around solid waste management in developing countries. Significant amounts of plastics are found in the environment globally (Cozar *et al.*, 2014). BPA and phthalates are often found in plastics while electronic waste (e-waste) contains PCBs, PBDEs, dioxins and plasticisers. The issue of e-waste is reviewed by Perkins *et al.* (2014), Needhidasan *et al.* (2014) and Grant *et al.* (2013).

1. Contaminated sites can be cleaned up, methods for decontamination depend on both the compound and substrate. Techniques and case studies are reviewed by Gomes *et al.* (2013). Methods of removing pesticides from soils are reviewed by Morillo and Villaverde (2017); Sudharshan *et al.* (2012) focuses on DDT. Bioremediation involves using bacteria and fungi to break the compounds down, reviewed by Shannon and Unterman (1993), Aislabie *et al.* (2010) and Passatore *et al.* (2014). Using high temperatures to remove PCBs from soil is discussed by Qi *et al.* (2014). Other emerging technologies discussed by Wang *et al.* (2017) and Rybnikova *et al.* (2016).
   1. The United States Environment Protection Agency Superfund program is described at US EPA (2017b). New Bedford Harbor is an example of a remediated site (Nelson & Bergen, 2012).
2. Strategies for preventing pharmaceuticals from entering the environment are reviewed by Blair (2016) and Straub (2016). Authorisation of veterinary medicines in the EU considers risks and benefits, including the risk of environmental harm (Chapman *et al.*, 2017). Guidelines regarding the use and disposal of veterinary medicines are described by Price and Tait (2012) for vets, and for farmers by the Veterinary Medicines Directorate (2014).
   1. Assessments of EDC effects in wildlife can be predicted from the “read across” hypothesis (Rand-Weaver *et al.*, 2013; Brown *et al.*, 2014). A database that connects drugs to their target proteins in unrelated species can be used to predict which species may be affected (Verbruggen *et al.*, 2018).

Examples of correlates between pharmaceutical sales/prescription data and levels in freshwater systems include ter Laak *et al.* (2014) and van Nuijs *et al.* (2015). Daughton (2014) discusses some limitations of this approach. Excretion rates of pharmaceuticals vary from 80-100% for metformin to 2-5% for paracetamol (Corcoran *et al.*, 2010; Scheurer *et al.*, 2012; Al-Odaini *et al.*, 2013). Bu *et al.* (2016) and Walters *et al.* (2010) review degradation rates in the freshwater and soils respectively.

- 1. Pharmaceuticals enter the environment via disposal of drugs, reviewed by Daughton and Ruhoy (2011). Drug takeback schemes discussed by Kampa *et al.* (2010).
     1. Castensson and Ekedahl (2010) review disposal of unused medication – 12-27% are figures for the UK,

- 1. Clark *et al.* (2010) and Kümmerer (2010) review some of the issues and chemistry involved in developing “greener” pharmaceuticals. The Swedish Environmental Classification of Pharmaceuticals classifies drugs on their environmental hazard properties and the risk to the aquatic environment (Stockholm County Council, 2014-2015).
  2. For details on urine separating toilets, see Blair (2016) and Lamichhane and Babcock (2012). A life cycle assessment that used the University of Florida campus as a case study (Landry & Boyer, 2016), suggested that urine separating toilets would have a 90% lower environmental impact due to saving 6.6 million gallons of water. However, decreasing pharmaceutical load using ozonation was more ecotoxic and cost 30% more.

1. As the CDC reports that no methods exist to remove PCBs from badly affected humans (CDC Agency for Toxic Substances and Disease Registry, 2014), we can conclude that some EDCs cannot be removed from wildlife under any circumstances. However, other EDCs such as BPA are readily eliminated from the body (Genuis *et al.*, 2012). For removal from the environment see ¶AB32.

1. Sumpter and Johnson (2005) discuss that for most fish species we do not have a good indication of what the rates of intersex in the absence of environmental pollutants would be i.e. what baseline rates are.
   1. For details of the UK Water Industry Research Limited’s monitoring programme, see UK WIR (2017) and Gardner *et al.* (2012), some of the findings are detailed in Gardner *et al.* (2012), Gardner *et al.* (2013) and (Comber *et al.*, 2018).
   2. Global monitoring of POPs in air water and human blood and breast milk under Stockholm Convention described by Hung *et al.* (2016), Magulova and Priceputu (2016) and Stockholm Convention (2008e). The HBM4EU project monitors a range of chemicals including some EDCs in humans across Europe (HBM4EU, 2017). EU raptor populations are monitored by European Cooperation in Science and Technology (2018) and described by Espin *et al.* (2016), while the Predatory Bird Monitoring Scheme in the UK (Centre for Hydrology & Ecology, 2018) is reviewed by Walker *et al.* (2008) and the otter project of Cardiff University (2018) is a long term surveillance scheme using otters found dead to measure contaminants. Levels of POPs can be correlated with other measures of health e.g. Kean *et al.* (2013).
   3. Examples of studies using museum specimens to determine effects of pollutants includes Reeder *et al.* (2005) who determined that intersex in Illinois cricket frogs only occurred following introduction of PCBs; Lind *et al.* (2003) and Sonne *et al.* (2004) showed that bone mineral density of Baltic grey seals and polar bears respectively was higher prior to the introduction of organochlorines; and Ratcliffe (1970) used museum collections to demonstrate that egg shells of various UK birds had thinned since about 1950 when DDT was introduced.

## (G) Future opportunities and challenges

1. Global population growth statistics by United Nations Population Fund (2018). For example, plastic production and use is expected to increase globally (Andrady & Neal, 2009; Thompson *et al.*, 2009). In India consumption of virgin plastic increased from 0.8kg per capita in 1990/91 to 3.2 kg in 2000/2001 in line with increases in GDP, and is predicted to increase six-fold between 2000 and 2030 (Mutha *et al.*, 2006). By 2050 an estimated 12,000 Mt of plastic waste is predicted to occur in landfills or the natural environment assuming current waste management practices (Geyer *et al.*, 2017). Modelling has suggested that population increases and demographic shifts will have a greater impact on steroid oestrogen concentrations in the Thames Basin than climate change, which is predicted to decrease water flow (Keller *et al.*, 2015).
2. Buchberger (2011) and Concha-Graña *et al.* (2013) review techniques to measure environmental levels of pharmaceuticals and organochloride pesticides respectively. der Beek *et al.* (2016) observed that the smaller spectrum of pharmaceuticals detected in samples from developing countries was most likely due to the lack of adequately equipped environmental laboratories. An example of a non-target screening programme is Norman Network (2012-2018), also see Dulio *et al.* (2018).
   1. Authors’ view, for discussion on issues with undertaking laboratory research on ED effects see ¶AB19.
3. One example is Toxcast (¶AB5.a). Messerlian *et al.* (2017) review how “omics” technologies can be used in EDC research. Difficulties in applying *in vitro* tests to individual effects is discussed by Futran Fuhrman *et al.* (2015). Adverse outcome pathways (AOPs) are described by OECD/OCDE (2018b) with application to EDCs by Ankley *et al.* (2010), Kramer *et al.* (2011) and Manibusan and Touart (2017).
   1. Gaps in endocrine testing methods discussed by Coady *et al.* (2017) and Manibusan and Touart (2017).
4. Gee (2013) discusses the balancing of risks for policy making. As an example, Sass and Colangelo (2006) and Ackerman (2007) both use atrazine to compare the US and EU approach to pesticide registration policy. Changing contraceptive use to non-hormonal methods (condoms) would decrease levels of EE2 in the environment, however the increased failure rate also needs consideration (Khan & Nicell, 2014), as the highest rates of oestrogen excretion are from pregnant women.
   1. Some attempts have been made to calculate the financial costs of EDC exposure on human health. Calculated estimates of the human health costs caused by EDCs in the European Union ranged from €714 million to €251 billion with a median of €1.63 billion or 1.28% of GDP (Trasande *et al.*, 2016). In the USA costs are estimated to be 2% of GDP (Attina *et al.*, 2016). Jaacks and Prasad (2017) comment that if costs to aquaculture and agriculture were included, human health costs would be higher.

Markandya *et al.* (2008) review the implications of the loss of the ecosystem service of cattle carcass removal services by vultures due to diclofenac. Without vultures, the cattle corpses were eaten by dogs, leading to an increase in the dog population and thus human cases of rabies, which are estimated to have cost US$34 billion 1993-2006.

There is a discussion of the economic benefits of TBT as an antifoulant boat paint in Evans (1999).

1. Reviews on the interaction between EDCs (particularly the POPs) and climate change include Alava *et al.* (2017), Nadal *et al.* (2015), Lamon *et al.* (2009), Noyes *et al.* (2009) and Schiedek *et al.* (2007). Jenssen (2006) review potential effects on Arctic fauna and Alava *et al.* (2017) reviews effects of climate change and pollutants on marine systems. The redistribution of some chemicals (e.g. PCBs) is projected to be greater than others such as PBDEs (Paul *et al.* (2012), differences in model findings discussed by Hansen *et al.* (2015). Keller *et al.* (2015) and Lu *et al.* (2015) review how climate change will affect the UK distribution of oestrogens and PCBs respectively.
   1. Noyes *et al.* (2009).
   2. Johnson *et al.* (2009a). Walsh *et al.* (2016) reviews present and future water resources in the Thames Basin.

- 1. Westra *et al.* (2014). For discussion surrounding changes in rainfall in the UK, see Dadson *et al.* (2017).

- 1. Schiedek *et al.* (2007) discusses stress generated by climate change and by EDCs and how they might interact. Studies investigating the interaction between EDCs and temperature include Little and Seebacher (2015), Brown *et al.* (2015), Patra *et al.* (2007) and Rohr *et al.* (2011).
  2. Shifts of polar bears diets in Hudson Bay (McKinney *et al.*, 2009) and East Greenland (McKinney *et al.*, 2013).
  3. Ma *et al.* (2011) modelled the remobilisation of POPs due melting sea ice in the Arctic. The melting of the Oberaar glacier in the Swiss Alps has caused POP levels in Lake Oberaar to increase from the late-1990’s (Weinhold, 2009; Bogdal *et al.*, 2010).

1. Changing burden of disease globally profiled in the Global Burden of Disease Study 2013 Collaborators (2015). Bech *et al.* (2011) review how aging population will alter health care expenditure. Increases in obesity and related co-morbidities (particularly diabetes) are covered by World Health Organization (2014) and World Health Organization (2016). The use of anti-depressants is expected to increase (Kantor *et al.*, 2015). Globally, contraceptive use is expected to increase (2015-2030) but declines are expected in some regions (United Nations Department of Economic and Social Affairs, 2015). Post-menopausal hormone replacement therapy has declined due to concerns surrounding side-effects (Sprague *et al.*, 2012).
   1. Personalised medicine is reviewed in Schork (2015) and by NHS England (2017). The use of nanotechnology in medicine is reviewed by Etheridge *et al.* (2013) and D'Mello *et al.* (2017). For details of “green pharmaceuticals” see ¶AB33.c.
2. For an overview of the EDC situation in Africa, see Bornman *et al.* (2017). Reviews of organochloride pesticide use in developing countries/regions include South Asia (Ali *et al.*, 2014), China (Li *et al.*, 2016), the Philippines (Mackintosh *et al.*, 2015) and Nigeria (Sindiku *et al.*, 2015). Use of EDCs may differ between regions, examples include phthalates (Berge *et al.*, 2013), alkylphenols (Berge *et al.*, 2012) and PBDEs (Li *et al.*, 2016). DDT is used for control of malaria mosquitoes in some countries (van den Berg, 2009). Pesticides banned under the Stockholm Convention are illegally used in some developing countries (Jan *et al.*, 2009; Ali *et al.*, 2014).

# References

Aas, C.B., Fuglei, E., Herzke, D., Yoccoz, N.G. & Routti, H. (2014) Effect of Body Condition on Tissue Distribution of Perfluoroalkyl Substances (PFASs) in Arctic Fox (*Vulpes lagopus*). *Environmental Science & Technology*, **48**, 11654-11661.

Ackerman, F. (2007) The economics of atrazine. *International Journal of Occupational and Environmental Health*, **13**, 437-45.

Adams, J., Speakman, T., Zolman, E., Mitchum, G., Wirth, E., Bossart, G.D. & Fair, P.A. (2014) The relationship between land use and emerging and legacy contaminants in an Apex predator, the bottlenose dolphin (*Tursiops truncatus*), from two adjacent estuarine watersheds. *Environmental Research*, **135**, 346-353.

Adeogun, A.O., Ibor, O.R., Omiwole, R.A., Hassan, T., Adegbola, R.A., Adewuyi, G.O. & Arukwe, A. (2015) Occurrence, Species, and Organ Differences in Bioaccumulation Patterns of Phthalate Esters in Municipal Domestic Water Supply Lakes in Ibadan, Nigeria. *Journal of Toxicology and Environmental Health A*, **78**, 761-77.

Agas, D., Sabbieti, M.G. & Marchetti, L. (2013) Endocrine disruptors and bone metabolism. *Archives of Toxicology*, **87**, 735-751.

Ahrens, L., Norstrom, K., Viktor, T., Cousins, A.P. & Josefsson, S. (2015) Stockholm Arlanda Airport as a source of per- and polyfluoroalkyl substances to water, sediment and fish. *Chemosphere*, **129**, 33-38.

Aislabie, J.M., Richards, N.K. & Boul, H.L. (2010) Microbial degradation of DDT and its residues—A review. *New Zealand Journal of Agricultural Research*, **40**, 269-282.

Al-Odaini, N.A., Zakaria, M.P., Yaziz, M.I., Surif, S. & Abdulghani, M. (2013) The occurrence of human pharmaceuticals in wastewater effluents and surface water of Langat River and its tributaries, Malaysia. *International Journal of Environmental Analytical Chemistry*, **93**, 245-264.

Alaee, M., Arias, P., Sjödin, A. & Bergman, Å. (2003) An overview of commercially used brominated flame retardants, their applications, their use patterns in different countries/regions and possible modes of release. *Environment International*, **29**, 683-689.

Alava, J.J., Cheung, W.W.L., Ross, P.S. & Sumaila, U.R. (2017) Climate change-contaminant interactions in marine food webs: Toward a conceptual framework. *Global Change Biology*, **23**, 3984-4001.

Alava, J.J., Keller, J.M., Wyneken, J., Crowder, L., Scott, G. & Kucklick, J.R. (2011a) Geographical variation of persistent organic pollutants in eggs of threatened loggerhead sea turtles (Caretta caretta) from southeastern United States. *Environmental Toxicology and Chemistry*, **30**, 1677-1688.

Alava, J.J., Lambourn, D., Olesiuk, P., Lance, M., Jeffries, S.J., Gobas, F.A.P.C. & Ross, P.S. (2012) PBDE flame retardants and PCBs in migrating Steller sea lions (*Eumetopias jubatus*) in the Strait of Georgia, British Columbia, Canada. *Chemosphere*, **88**, 855-864.

Alava, J.J., Salazar, S., Cruz, M., Jimenez-Uzcategui, G., Villegas-Amtmann, S., Paez-Rosas, D., Costa, D.P., Ross, P.S., Ikonomou, M.G. & Gobas, F.A.P.C. (2011b) DDT Strikes Back: Galapagos Sea Lions Face Increasing Health Risks. *Ambio*, **40**, 425-430.

Ali, N., Harrad, S., Goosey, E., Neels, H. & Covaci, A. (2011) "Novel" brominated flame retardants in Belgian and UK indoor dust: implications for human exposure. *Chemosphere*, **83**, 1360-5.

Ali, U., Syed, J.H., Malik, R.N., Katsoyiannis, A., Li, J., Zhang, G. & Jones, K.C. (2014) Organochlorine pesticides (OCPs) in South Asian region: A review. *Science of The Total Environment*, **476-477**, 705-717.

Allchin, C.R., Law, R.J. & Morris, S. (1999) Polybrominated diphenylethers in sediments and biota downstream of potential sources in the UK. *Environmental Pollution*, **105**, 197-207.

Alvarez, D.A., Shappell, N.W., Billey, L.O., Bermudez, D.S., Wilson, V.S., Kolpin, D.W., Perkins, S.D., Evans, N., Foreman, W.T., Gray, J.L., Shipitalo, M.J. & Meyer, M.T. (2013) Bioassay of estrogenicity and chemical analyses of estrogens in streams across the United States associated with livestock operations. *Water Research*, **47**, 3347-63.

Alzieu, C. (2000) Environmental impact of TBT: the French experience. *Science of The Total Environment*, **258**, 99-102.

Amend, L.J. & Lederman, P.B. (1992) Critical evaluation of PCB remediation technologies. *Environmental Progress*, **11**, 173-177.

Andersen, M.S., Fuglie, E., Konig, M., Lipasti, I., Pedersen, A.O., Polder, A., Yoccoz, N.G. & Routti, H. (2015) Levels and temporal trends of persistent organic pollutants (POPs) in arctic foxes (*Vulpes lagopus*) from Svalbard in relation to dietary habits and food availability. *Science of the Total Environment*, **511**, 112-122.

Anderson, D., Jehl, J., Risebrough, R., Woods, L., Deweese, L. & Edgecomb, W. (1975) Brown pelicans: improved reproduction off the southern California coast. *Science*, **190**, 806-808.

Anderson, P.D., D'Aco, V.J., Shanahan, P., Chapra, S.C., Buzby, M.E., Cunningham, V.L., Duplessie, B.M., Hayes, E.P., Mastrocco, F.J., Parke, N.J., Rader, J.C., Samuelian, J.H. & Schwab, B.W. (2004) Screening analysis of human pharmaceutical compounds in US surface waters. *Environmental Science & Technology*, **38**, 838-849.

Andrady, A.L. & Neal, M.A. (2009) Applications and societal benefits of plastics. *Philosophical Transactions of the Royal Society of London B — Biological Science*, **364**, 1977-84.

Ankley, G.T. & Gray, L.E. (2013) Cross-species conservation of endocrine pathways: a critical analysis of tier 1 fish and rat screening assays with 12 model chemicals. *Environmental Toxicology and Chemistry*, **32**, 1084-7.

Ankley, G.T., Bennett, R.S., Erickson, R.J., Hoff, D.J., Hornung, M.W., Johnson, R.D., Mount, D.R., Nichols, J.W., Russom, C.L., Schmieder, P.K., Serrrano, J.A., Tietge, J.E. & Villeneuve, D.L. (2010) Adverse outcome pathways: a conceptual framework to support ecotoxicology research and risk assessment. *Environmental Toxicology and Chemistry*, **29**, 730-41.

Antizar-Ladislao, B. (2008) Environmental levels, toxicity and human exposure to tributyltin (TBT)-contaminated marine environment. A review. *Environment International*, **34**, 292-308.

Aoki, K.A.A., Harris, C.A., Katsiadaki, I. & Sumpter, J.P. (2011) Evidence suggesting that di-n-butyl phthalate has antiandrogenic effects in fish. *Environmental Toxicology and Chemistry*, **30**, 1338-1345.

Arnot, J.A. & Gobas, F.A.P.C. (2006) A review of bioconcentration factor (BCF) and bioaccumulation factor (BAF) assessments for organic chemicals in aquatic organisms. *Environmental Reviews*, **14**, 257-297.

Arukwe, A., Eggen, T. & Moeder, M. (2012) Solid waste deposits as a significant source of contaminants of emerging concern to the aquatic and terrestrial environments - A developing country case study from Owerri, Nigeria. *Science of the Total Environment*, **438**, 94-102.

Attina, T.M., Hauser, R., Sathyanarayana, S., Hunt, P.A., Bourguignon, J.-P., Myers, J.P., DiGangi, J., Zoeller, R.T. & Trasande, L. (2016) Exposure to endocrine-disrupting chemicals in the USA: a population-based disease burden and cost analysis. *The Lancet Diabetes & Endocrinology*, **4**, 996-1003.

Aulerich, R.J. & Ringer, R.K. (1977) Current Status of PCB Toxicity to Mink, and Effect on Their Reproduction. *Archives of Environmental Contamination and Toxicology*, **6**, 279-292.

Aulerich, R.J., Ringer, R.K., Seagran, H.L. & Youatt, W.G. (1971) Effects of feeding coho salmon and other Great Lakes fish on mink reproduction. *Canadian Journal of Zoology*, **49**, 611-616.

Aviv, R. (2014) A Valuable Reputation. *The New Yorker*, **10 February 2014**, <http://www.newyorker.com/magazine/2014/02/10/a-valuable-reputation>.

Awad, E., Zhang, X., Bhavsar, S.P., Petro, S., Crozier, P.W., Reiner, E.J., Fletcher, R., Tittemier, S.A. & Braekevelt, E. (2011) Long-Term Environmental Fate of Perfluorinated Compounds after Accidental Release at Toronto Airport. *Environmental Science & Technology*, **45**, 8081-8089.

Azuma, N., Miranda, R.M., Goshima, S. & Abe, S. (2014) Phylogeography of Neptune whelk (*Neptunea arthritica*) suggests sex-biased impact of tributyltin pollution and overfishing around northern Japan. *Journal of Molluscan Studies*, **81**, 131-138.

Bachman, M.J., Keller, J.M., West, K.L. & Jensen, B.A. (2014) Persistent organic pollutant concentrations in blubber of 16 species of cetaceans stranded in the Pacific Islands from 1997 through 2011. *Science of the Total Environment*, **488**, 115-123.

Bahamonde, P.A., Munkittrick, K.R. & Martyniuk, C.J. (2013) Intersex in teleost fish: Are we distinguishing endocrine disruption from natural phenomena? *General and Comparative Endocrinology*, **192**, 25-35.

Balch, G.C., Velez-Espino, L.A., Sweet, C., Alaee, M. & Metcalfe, C.D. (2006) Inhibition of metamorphosis in tadpoles of *Xenopus laevis* exposed to polybrominated diphenyl ethers (PBDEs). *Chemosphere*, **64**, 328-338.

Bartrons, M., Grimalt, J.O., de Mendoza, G. & Catalan, J. (2012) Pollutant Dehalogenation Capability May Depend on the Trophic Evolutionary History of the Organism: PBDEs in Freshwater Food Webs. *Plos One*, **7**

Basel Convention (2011) *Home*. <http://www.basel.int/>.

Basile, E.R., Avery, H.W., Bien, W.F. & Keller, J.M. (2011) Diamondback terrapins as indicator species of persistent organic pollutants: Using Barnegat Bay, New Jersey as a case study. *Chemosphere*, **82**, 137-144.

Basu, N. & Head, J. (2010) Mammalian wildlife as complementary models in environmental neurotoxicology. *Neurotoxicology and Teratology*, **32**, 114-119.

Basu, N., Scheuhammer, A.M., Bursian, S.J., Elliott, J., Rouvinen-Watt, K. & Chan, H.M. (2007) Mink as a sentinel species in environmental health. *Environmental Research*, **103**, 130-44.

Baumann, L., Knoerr, S., Keiter, S., Nagel, T., Rehberger, K., Volz, S., Oberrauch, S., Schiller, V., Fenske, M., Holbech, H., Segner, H. & Braunbeck, T. (2014) Persistance of endocrine disruption in zebrafish (*Danio rerio*) after discontinued exposire to the androgen 17 beta-trenbolone. *Environmental Toxicology and Chemistry*, **33**, 2488-2496.

Baynes, A., Green, C., Nicol, E., Beresford, N., Kanda, R., Henshaw, A., Churchley, J. & Jobling, S. (2012) Additional Treatment of Wastewater Reduces Endocrine Disruption in Wild Fish - A Comparative Study of Tertiary and Advanced Treatments. *Environmental Science & Technology*, **46**, 5565-5573.

Bean, T.G., Boxall, A.B.A., Lane, J., Herborn, K.A., Pietravalle, S. & Arnold, K.E. (2014) Behavioural and physiological responses of birds to environmentally relevant concentrations of an antidepressant. *Philosophical Transactions of the Royal Society of London B: Biological Sciences*, **369**

Beausoleil, C., Ormsby, J.-N., Gies, A., Hass, U., Heindel, J.J., Holmer, M.L., Nielsen, P.J., Munn, S. & Schoenfelder, G. (2013) Low dose effects and non-monotonic dose responses for endocrine active chemicals: Science to practice workshop: Workshop summary. *Chemosphere*, **93**, 847-856.

Bech, M., Christiansen, T., Khoman, E., Lauridsen, J. & Weale, M. (2011) Ageing and health care expenditure in EU-15. *The European Journal of Health Economics*, **12**, 469-78.

Beckett, K.J., Millsap, S.D., Blankenship, A.L., Zwiernik, M.J., Giesy, J.P. & Bursian, S.J. (2005) Squamous Epithelial Lesion of the Mandibles and Maxillae of Wild Mink (*Mustela vison*) Naturally Exposed to Polychlorinated Biphenyls. *Environmental Toxicology and Chemistry*, **24**, 674.

Beckmen, K.B., Blake, J.E., Ylitalo, G.M., Stott, J.L. & O’Hara, T.M. (2003) Organochlorine contaminant exposure and associations with hematological and humoral immune functional assays with dam age as a factor in free-ranging northern fur seal pups (*Callorhinus ursinus*). *Marine Pollution Bulletin*, **46**, 594-606.

Beineke, A., Siebert, U., McLachlan, M., Bruhn, R., Thron, K., Failing, K., Muller, G. & Baumgartner, W. (2005) Investigations of the potential influence of environmental contaminants on the thymus and spleen of harbor porpoises (*Phocoena phocoena*). *Environmental Science & Technology*, **39**, 3933-3938.

Béland, P., DeGuise, S., Girard, C., Lagacé, A., Martineau, D., Michaud, R., Muir, D.C.G., Norstrom, R.J., Pelletier, É., Ray, S. & Shugart, L.R. (1993) Toxic Compounds and Health and Reproductive Effects in St. Lawrence Beluga Whales. *Journal of Great Lakes Research*, **19**, 766-775.

Berg, C., Blomqvist, A., Holm, L., Brandt, I., Brunstrom, B. & Ridderstrale, Y. (2004) Embryonic exposure to oestrogen causes eggshell thinning and altered shell gland carbonic anhydrase expression in the domestic hen. *Reproduction*, **128**, 455-61.

Berg, T., Furhaupter, K., Teixeira, H., Uusitalo, L. & Zampoukas, N. (2015) The Marine Strategy Framework Directive and the ecosystem-based approach - pitfalls and solutions. *Marine Pollution Bulletin*, **96**, 18-28.

Berge, A., Cladiere, M., Gasperi, J., Coursimault, A., Tassin, B. & Moilleron, R. (2012) Meta-analysis of environmental contamination by alkylphenols. *Environmental Science and Pollution Research*, **19**, 3798-819.

Berge, A., Cladiere, M., Gasperi, J., Coursimault, A., Tassin, B. & Moilleron, R. (2013) Meta-analysis of environmental contamination by phthalates. *Environmental Science and Pollution Research*, **20**, 8057-76.

Bergman, A., Heindel, J.J., Jobling, S., Kidd, K.A. & Zoeller, R.T. (2012) *State of the Science of Endocrine Disrupting Chemicals - 2012*. World Health Organization, <http://www.who.int/ceh/publications/endocrine/en/>.

Bergman, Å., Becher, G., Blumberg, B., Bjerregaard, P., Bornman, R., Brandt, I., Casey, S.C., Frouin, H., Giudice, L.C., Heindel, J.J., Iguchi, T., Jobling, S., Kidd, K.A., Kortenkamp, A., Lind, P.M., Muir, D., Ochieng, R., Ropstad, E., Ross, P.S., Skakkebaek, N.E., Toppari, J., Vandenberg, L.N., Woodruff, T.J. & Zoeller, R.T. (2015) Manufacturing doubt about endocrine disrupter science – A rebuttal of industry-sponsored critical comments on the UNEP/WHO report “State of the Science of Endocrine Disrupting Chemicals 2012”. *Regulatory Toxicology and Pharmacology*, **73**, 1007-1017.

Bertram, M.G., Saaristo, M., Baumgartner, J.B., Johnstone, C.P., Allinson, M., Allinson, G. & Wong, B.B.M. (2015) Sex in troubled waters: Widespread agricultural contaminant disrupts reproductive behaviour in fish. *Hormones and Behavior*, **70**, 85-91.

Beyer, A. & Biziuk, M. (2009) Environmental Fate and Global Distribution of Polychlorinated Biphenyls. *Reviews of Environmental Contamination and Toxicology Vol 201* (ed. by D.M. Whitacre), pp. 137-158. Springer US.

Bhandari, R.K., vom Saal, F.S. & Tillitt, D.E. (2015a) Transgenerational effects from early developmental exposures to bisphenol A or 17α-ethinylestradiol in medaka, *Oryzias latipes*. *Scientific Reports*, **5**, 9303.

Bhandari, R.K., Deem, S.L., Holliday, D.K., Jandegian, C.M., Kassotis, C.D., Nagel, S.C., Tillitt, D.E., Saal, F.S.V. & Rosenfeld, C.S. (2015b) Effects of the environmental estrogenic contaminants bisphenol A and 17 alpha-ethinyl estradiol on sexual development and adult behaviors in aquatic wildlife species. *General and Comparative Endocrinology*, **214**, 195-219.

Bhatia, H., Kumar, A., Chapman, J.C. & McLaughlin, M.J. (2015) Long-term exposures to di-n-butyl phthalate inhibit body growth and impair gonad development in juvenile Murray rainbowfish (*Melanotaenia fluviatilis*). *Journal of Applied Toxicology*, **35**, 806-816.

Birch, G.F., Apostolatos, C. & Taylor, S.E. (2013) A Remarkable Recovery in the Sydney Rock Oyster (*Saccostrea glomerata*) Population in a Highly Urbanised Estuary (Sydney Estuary, Australia). *Journal of Coastal Research*, **29**, 1009-1015.

Biswas, S., Shapiro, C.A., Kranz, W.L., Mader, T.L., Shelton, D.P., Snow, D.D., Bartelt-Hunt, S.L., Tarkalson, D.D., van Donk, S.J., Zhang, T.C. & Ensley, S. (2013) Current knowledge on the environmental fate, potential impact, and management of growth-promoting steroids used in the US beef cattle industry. *Journal of Soil and Water Conservation*, **68**, 325-336.

Blair, B.D. (2016) Potential Upstream Strategies for the Mitigation of Pharmaceuticals in the Aquatic Environment: a Brief Review. *Current Environmental Health Reports*, **3**, 153-60.

Blair, B.D., Crago, J.P., Hedman, C.J. & Klaper, R.D. (2013) Pharmaceuticals and personal care products found in the Great Lakes above concentrations of environmental concern. *Chemosphere*, **93**, 2116-23.

Blanchfield, P.J., Kidd, K.A., Docker, M.F., Palace, V.P., Park, B.J. & Postma, L.D. (2015) Recovery of a Wild Fish Population from Whole-Lake Additions of a Synthetic Estrogen. *Environmental Science & Technology*, **49**, 3136-3144.

Blount, B.C., Pirkle, J.L., Osterloh, J.D., Valentin-Blasini, L. & Caldwell, K.L. (2006) Urinary perchlorate and thyroid hormone levels in adolescent and adult men and women living in the United States. *Environmental Health Perspectives*, **114**, 1865-71.

Boas, M., Feldt-Rasmussen, U., Skakkebaek, N.E. & Main, K.M. (2006) Environmental chemicals and thyroid function. *European Journal of Endocrinology*, **154**, 599-611.

Boberg, J., Taxvig, C., Christiansen, S. & Hass, U. (2010) Possible endocrine disrupting effects of parabens and their metabolites. *Reproductive Toxicology*, **30**, 301-12.

Bogdal, C., Nikolic, D., Luthi, M.P., Schenker, U., Scheringer, M. & Hungerbuhler, K. (2010) Release of legacy pollutants from melting glaciers: model evidence and conceptual understanding. *Environ Sci Technol*, **44**, 4063-9.

Bornman, M.S., Aneck-Hahn, N.H., de Jager, C., Wagenaar, G.M., Bouwman, H., Barnhoorn, I.E.J., Patrick, S.M., Vandenberg, L.N., Kortenkamp, A., Blumberg, B., Kimmins, S., Jegou, B., Auger, J., DiGangi, J. & Heindel, J.J. (2017) Endocrine Disruptors and Health Effects in Africa: A Call for Action. *Environmental Health Perspectives*, **125**, 085005.

Bosker, T., Munkittrick, K.R. & Maclatchy, D.L. (2010) Challenges and opportunities with the use of biomarkers to predict reproductive impairment in fishes exposed to endocrine disrupting substances. *Aquatic Toxicology*, **100**, 9-16.

Bourguignon, J.P., Slama, R., Bergman, A., Demeneix, B., Ivell, R., Kortenkamp, A., Panzica, G., Trasande, L. & Zoeller, R.T. (2016) Science-based regulation of endocrine disrupting chemicals in Europe: which approach? *Lancet Diabetes & Endocrinology*, **4**, 643-646.

Bouwman, H., van den Berg, H. & Kylin, H. (2011) DDT and malaria prevention: addressing the paradox. *Environmental Health Perspectives*, **119**, 744-7.

Bouwman, H., Bornman, R., Berg, H.v.d. & Kylin, H. (2013) DDT: fifty years since Silent Spring. *Late lessons from early warnings: science, precaution, innovation* (ed. by D. Gee). EEA Report No 1/2013, European Environment Agency, Copenhagen.

Bovee, T.F., Helsdingen, R.J., Hamers, A.R., van Duursen, M.B., Nielen, M.W. & Hoogenboom, R.L. (2007) A new highly specific and robust yeast androgen bioassay for the detection of agonists and antagonists. *Analytical and Bioanalytical Chemistry*, **389**, 1549-58.

Bowerman, W.W., Best, D.A., Grubb, T.G., Sikarskie, J.G. & Giesy, J.P. (2000) Assessment of environmental endocrine disruptors in bald eagles of the Great Lakes. *Chemosphere*, **41**, 1569-1574.

Bowerman, W.W., Best, D.A., Giesy, J.P., Shieldcastle, M.C., Meyer, M.W., Postupalsky, S. & Sikarskie, J.G. (2003) Associations between regional differences in polychlorinated biphenyls and dichlorodiphenyldichloroethylene in blood of nestling bald eagles and reproductive productivity. *Environmental Toxicology and Chemistry*, **22**, 371-376.

Boxall, A.B.A., Sinclair, C.J., Fenner, K., Kolpin, D. & Maund, S.J. (2004) When Synthetic Chemicals Degrade in the Environment. *Environmental Science & Technology*, **38**, 368A-375A.

Brandhuber, P., Clark, S. & Morley, K. (2009) A review of perchlorate occurence in public drinking water systems. *Journal AWWA*, **101**, 63-73. <https://www.awwa.org/publications/journal-awwa/abstract/articleid/22200.aspx>.

Breivik, K., Gioia, R., Chakraborty, P., Zhang, G. & Jones, K.C. (2011) Are reductions in industrial organic contaminants emissions in rich countries achieved partly by export of toxic wastes? *Environmental Science & Technology*, **45**, 9154-60.

Brian, J.V., Harris, C.A., Scholze, M., Backhaus, T., Booy, P., Lamoree, M., Pojana, G., Jonkers, N., Runnalls, T., Bonfà, A., Marcomini, A. & Sumpter, J.P. (2005) Accurate Prediction of the Response of Freshwater Fish to a Mixture of Estrogenic Chemicals. *Environmental Health Perspectives*, **113**, 721-728.

British National Formulary (2016) *Metformin hydrochloride*. <https://www.evidence.nhs.uk/formulary/bnf/current/6-endocrine-system/61-drugs-used-in-diabetes/612-antidiabetic-drugs/6122-biguanides/metformin-hydrochloride>.

Brown, A.R., Gunnarsson, L., Kristiansson, E. & Tyler, C.R. (2014) Assessing variation in the potential susceptibility of fish to pharmaceuticals, considering evolutionary differences in their physiology and ecology. *Philosophical Transactions of the Royal Society of London B — Biological Sciences*, **369**

Brown, A.R., Owen, S.F., Peters, J., Zhang, Y., Soffker, M., Paull, G.C., Hosken, D.J., Wahab, M.A. & Tyler, C.R. (2015) Climate change and pollution speed declines in zebrafish populations. *Proceedings of the National Academy of Sciences of the United States of America*, **112**, E1237-E1246.

Bruinen de Bruin, Y., Hakkinen, P.B., Lahaniatis, M., Papameletiou, D., Del Pozo, C., Reina, V., Van Engelen, J., Heinemeyer, G., Viso, A.C., Rodriguez, C. & Jantunen, M. (2007) Risk management measures for chemicals in consumer products: documentation, assessment, and communication across the supply chain. *Journal of Exposure Science and Environmental Epidemiology*, **17 Suppl 1**, S55-66.

Brunström, B., Lund, B.-O., Bergman, A., Asplund, L., Athanassiadis, I., Athanasiadou, M., Jensen, S. & Örberg, J. (2001) Reproductive toxicity in mink (*Mustela vison*) chronically exposed to environmentally relevant polychlorinated biphenyl concentrations. *Environmental Toxicology and Chemistry*, **20**, 2318-2327.

Bu, Q., Shi, X., Yu, G., Huang, J. & Wang, B. (2016) Assessing the persistence of pharmaceuticals in the aquatic environment: Challenges and needs. *Emerging Contaminants*, **2**, 145-147.

Buchberger, W.W. (2011) Current approaches to trace analysis of pharmaceuticals and personal care products in the environment. *Journal of Chromatography A*, **1218**, 603-18.

Buck, R.C., Franklin, J., Berger, U., Conder, J.M., Cousins, I.T., de Voogt, P., Jensen, A.A., Kannan, K., Mabury, S.A. & van Leeuwen, S.P. (2011) Perfluoroalkyl and polyfluoroalkyl substances in the environment: terminology, classification, and origins. *Integrated Environmental Assessment and Management*, **7**, 513-41.

Bulaeva, E., Lanctot, C., Reynolds, L., Trudeau, V.L. & Navarro-Martin, L. (2015) Sodium perchlorate disrupts development and affects metamorphosis- and growth-related gene expression in tadpoles of the wood frog (*Lithobates sylvaticus*). *General and Comparative Endocrinology*, **222**, 33-43.

Bursian, S.J., Kern, J., Remington, R.E., Link, J.E. & Fitzgerald, S.D. (2013) Dietary exposure of mink (*Mustela vison*) to fish from the upper Hudson River, New York, USA: effects on reproduction and offspring growth and mortality. *Environmental Toxicology and Chemistry*, **32**, 780-93.

Bursian, S.J., Sharma, C., Aulerich, R.J., Yamini, B., Mitchell, R.R., Orazio, C.E., Moore, D.R., Svirsky, S. & Tillitt, D.E. (2006) Dietary exposure of mink (*Mustela vison*) to fish from the Housatonic river, Berkshire County, Massachusetts, USA: effects on reproduction, kit growth, and survival. *Environmental Toxicology and Chemistry*, **25**, 1533-40.

Business Wire (2016) *Baby Bottles Market to Gain Momentum Owing to the Increasing Production of Formula Milk Until 2021, Says Technavio*. <http://www.businesswire.com/news/home/20161102006347/en/Baby-Bottles-Market-Gain-Momentum-Owing-Increasing>.

Bustnes, J.O., Erikstad, K.E., Lorentsen, S.-H. & Herzke, D. (2008) Perfluorinated and chlorinated pollutants as predictors of demographic parameters in an endangered seabird. *Environmental Pollution*, **156**, 417-424.

Bustnes, J.O., Tveraa, T., Varpe, O., Henden, J.A. & Skaare, J.U. (2007) Reproductive performance and organochlorine pollutants in an Antarctic marine top predator: The south polar skua. *Environment International*, **33**, 911-918.

Bustnes, J.O., Tveraa, T., Henden, J.A., Varpe, O., Janssen, K. & Skaare, J.U. (2006) Organochlorines in Antarctic and Arctic avian top predators: A comparison between the south polar skua and two species of northern hemisphere gulls. *Environmental Science & Technology*, **40**, 2826-2831.

Bytingsvik, J., Lie, E., Aars, J., Derocher, A.E., Wiig, O. & Jenssen, B.M. (2012a) PCBs and OH-PCBs in polar bear mother-cub pairs: A comparative plasma levels in 1998 and 2008. *Science of the Total Environment*, **417**, 117-128.

Bytingsvik, J., van Leeuwen, S.P.J., Hamers, T., Swart, K., Aars, J., Lie, E., Nilsen, E.M.E., Wiig, O., Derocher, A.E. & Jenssen, B.M. (2012b) Perfluoroalkyl substances in polar bear mother-cub pairs: A comparative study based on plasma levels from 1998 and 2008. *Environment International*, **49**, 92-99.

Cajthaml, T., Kresinova, Z., Svobodova, K., Sigler, K. & Rezanka, T. (2009) Microbial transformation of synthetic estrogen 17alpha-ethinylestradiol. *Environmental Pollution*, **157**, 3325-35.

Campbell, C.G., Borglin, S.E., Green, F.B., Grayson, A., Wozei, E. & Stringfellow, W.T. (2006) Biologically directed environmental monitoring, fate, and transport of estrogenic endocrine disrupting compounds in water: A review. *Chemosphere*, **65**, 1265-1280.

Campos, B., Rivetti, C., Kress, T., Barata, C. & Dircksen, H. (2016) Depressing Antidepressant: Fluoxetine Affects Serotonin Neurons Causing Adverse Reproductive Responses in *Daphnia magna*. *Environmental Science & Technology*, **50**, 6000-6007.

Capiotti, K.M., Antonioli Junior, R., Kist, L.W., Bogo, M.R., Bonan, C.D. & Da Silva, R.S. (2014) Persistent impaired glucose metabolism in a zebrafish hyperglycemia model. *Comparative Biochemistry and Physiology B-Biochemistry & Molecular Biology*, **171**, 58-65.

Cardiff University (2018) *Otter Project*. <http://www.cardiff.ac.uk/otter-project/about-us>.

Castensson, S. & Ekedahl, A. (2010) Pharmaceutical Waste: The Patient Role. *Green and Sustainable Pharmacy* (ed. by K. KüMmerer and M. Hempel). Springer-Verlag, Berlin.

Cauley, J.A. (2015) Estrogen and bone health in men and women. *Steroids*, **99**, 11-5.

CDC (2016) *National Biomonitoring Program. Fatsheet: Phthalates*. <https://www.cdc.gov/biomonitoring/phthalates_factsheet.html>.

CDC Agency for Toxic Substances and Disease Registry (2014) *Polychlorinated Biphenyls (PCBs) Toxicity: How Should Patients Exposed to PCBs Be Treated and Managed?* <https://www.atsdr.cdc.gov/csem/csem.asp?csem=30&po=13>.

Celtic Recycling (2009) *PCB Disposal & Decontamination*. Available at: <http://www.celtic-recycling.co.uk/pcb-disposal-and-decontamination.html> (accessed

Centre for Hydrology & Ecology (2018) *Predatory Bird Monitoring Scheme*. <https://pbms.ceh.ac.uk/>.

Cerveny, D., Turek, J., Grabic, R., Golovko, O., Koba, O., Fedorova, G., Grabicova, K., Zlabek, V. & Randak, T. (2016) Young-of-the-year fish as a prospective bioindicator for aquatic environmental contamination monitoring. *Water research*, **103**, 334-42.

Chandler, D., Bending, G., Clarkson, J., Davidson, G., Hall, S., Mills, P., Pink, D., Skirvin, D., Neve, P., Kennedy, R., Greaves, J.M., Grant, W.P. & Collier, R.H. (2008) The Consequences of the ‘cut off’ Criteria for Pesticides: Alternative Methods of Cultivation *European Parliament: Policy Department B, Structural and Cohension Policies*, IP/B/AGRI/IC/2008_168, <http://www.europarl.europa.eu/RegData/etudes/note/join/2008/408962/IPOL-AGRI_NT(2008)408962_EN.pdf>.

Chang, S.C., Thibodeaux, J.R., Eastvold, M.L., Ehresman, D.J., Bjork, J.A., Froehlich, J.W., Lau, C., Singh, R.J., Wallace, K.B. & Butenhoff, J.L. (2008) Thyroid hormone status and pituitary function in adult rats given oral doses of perfluorooctanesulfonate (PFOS). *Toxicology*, **243**, 330-9.

Chapman, J.L., Porsch, L., Vidaurre, R., Backhaus, T., Sinclair, C., Jones, G. & Boxall, A.B.A. (2017) Three methods for integration of environmental risk into the benefit-risk assessment of veterinary medicinal products. *Science of the Total Environment*, **605-606**, 692-701.

Chen, D., Kannan, K., Tan, H.L., Zheng, Z.G., Feng, Y.L., Wu, Y. & Widelka, M. (2016) Bisphenol Analogues Other Than BPA: Environmental Occurrence, Human Exposure, and Toxicity-A Review. *Environmental Science & Technology*, **50**, 5438-5453.

Chen, D., Zhang, X., Mai, B., Sun, Q., Song, J., Luo, X., Zeng, E.Y. & Hale, R.C. (2009) Polychlorinated biphenyls and organochlorine pesticides in various bird species from northern China. *Environmental Pollution*, **157**, 2023-9.

Chen, T.S., Chen, T.C., Yeh, K.J., Chao, H.R., Liaw, E.T., Hsieh, C.Y., Chen, K.C., Hsieh, L.T. & Yeh, Y.L. (2010) High estrogen concentrations in receiving river discharge from a concentrated livestock feedlot. *Science of the Total Environment*, **408**, 3223-30.

Cheng, H., Lin, T., Zhang, G., Liu, G., Zhang, W., Qi, S., Jones, K.C. & Zhang, X. (2014) DDTs and HCHs in sediment cores from the Tibetan Plateau. *Chemosphere*, **94**, 183-9.

Cheng, Y., Cui, Y., Chen, H.M. & Xie, W.P. (2011) Thyroid disruption effects of environmental level perfluorooctane sulfonates (PFOS) in *Xenopus laevis*. *Ecotoxicology*, **20**, 2069-78.

Christensen, H., Heggberget, T. & Gutleb, A. (2010) Polychlorinated Biphenyls and Reproductive Performance in Otters From the Norwegian Coast. *Archives of Environmental Contamination and Toxicology*, **59**, 652-660.

Christensen, J.R., MacDuffee, M., Macdonald, R.W., Whiticar, M. & Ross, P.S. (2005) Persistent Organic Pollutants in British Columbia Grizzly Bears:  Consequence of Divergent Diets. *Environmental Science & Technology*, **39**, 6952-6960.

Chung, E., Genco, M.C., Megrelis, L. & Ruderman, J.V. (2011) Effects of bisphenol A and triclocarban on brain-specific expression of aromatase in early zebrafish embryos. *Proceedings of the National Academy of Sciences*, **108**, 17732-17737.

Ciocan, C.M., Cubero-Leon, E., Puinean, A.M., Hill, E.M., Minier, C., Osada, M., Fenlon, K. & Rotchell, J.M. (2010) Effects of estrogen exposure in mussels, *Mytilus edulis,* at different stages of gametogenesis. *Environmental Pollution*, **158**, 2977-84.

Ciriminna, R., Bright, F.V. & Pagliaro, M. (2015) Ecofriendly Antifouling Marine Coatings. *ACS Sustainable Chemistry & Engineering*, **3**, 559-565.

Clark, J.H., Breeden, S.W. & Summerton, L. (2010) Green(er) Pharmacy. *Green and Sustainable Pharmacy* (ed. by K. KüMmerer and M. Hempel). Springer-Verlag, Berlin.

Clewell, R.A., Merrill, E.A., Narayanan, L., Gearhart, J.M. & Robinson, P.J. (2004) Evidence for competitive inhibition of iodide uptake by perchlorate and translocation of perchlorate into the thyroid. *International Journal of Toxicology*, **23**, 17-23.

Coady, K.K., Biever, R.C., Denslow, N.D., Gross, M., Guiney, P.D., Holbech, H., Karouna-Renier, N.K., Katsiadaki, I., Krueger, H., Levine, S.L., Maack, G., Williams, M., Wolf, J.C. & Ankley, G.T. (2017) Current limitations and recommendations to improve testing for the environmental assessment of endocrine active substances. *Integrated Environmental Assessment and Management*, **13**, 302-316.

Colabuono, F.I., Taniguchi, S., Petry, M.V. & Montone, R.C. (2015) Organochlorine contaminants and polybrominated diphenyl ethers in eggs and embryos of Antarctic birds. *Antarctic Science*, **27**, 355-361.

Colborn, T., Dumanoski, D. & Myers, J.P. (1996) *Our Stolen Future: are we threatening our fertility, intelligence and survival? A scientific dectective story*. Little, Brown and Company.

Colli-Dula, R.C., Martyniuk, C.J., Streets, S., Denslow, N.D. & Lehr, R. (2016) Molecular impacts of perfluorinated chemicals (PFASs) in the liver and testis of male largemouth bass (*Micropterus salmoides*) in Minnesota Lakes. *Comparative Biochemistry and Physiology D - Genomics & Proteomics*, **19**, 129-139.

Colson, I. & Hughes, R.N. (2004) Rapid recovery of genetic diversity of dogwhelk (*Nucella lapillus* L.) populations after local extinction and recolonization contradicts predictions from life-history characteristics. *Molecular Ecology*, **13**, 2223-33.

Comber, S., Gardner, M., Sorme, P., Leverett, D. & Ellor, B. (2018) Active pharmaceutical ingredients entering the aquatic environment from wastewater treatment works: A cause for concern? *Science of the Total Environment*, **613-614**, 538-547.

Concha-Graña, E., Turnes-Carou, I., Muniategui-Lorenzo, S., López-Mahía, P. & Prada-Rodríguez, D. (2013) Influence of matrix on suitability of four methods for organochlorine pesticide analysis in waters. *International Journal of Environmental Analytical Chemistry*, **93**, 416-433.

Conners, D.E., Rogers, E.D., Armbrust, K.L., Kwon, J.W. & Black, M.C. (2009) Growth and development of tadpoles (*Xenopus laevis*) exposed to selective serotonin reuptake inhibitors, fluoxetine and sertraline, throughout metamorphosis. *Environmental Toxicology and Chemistry*, **28**, 2671-6.

Cooke, A.S. (1973) Shell thinning in avian eggs by environmental pollutants. *Environmental Pollution*, **4**, 85-152.

Corcoran, J., Winter, M.J. & Tyler, C.R. (2010) Pharmaceuticals in the aquatic environment: a critical review of the evidence for health effects in fish. *Critical Reviews in Toxicology*, **40**, 287-304.

Couderc, M., Marchand, J., Zalouk-Vergnoux, A., Kamari, A., Moreau, B., Blanchet-Letrouye, I., Le Bizec, B., Mouneyrac, C. & Poirier, L. (2016) Thyroid endocrine status of wild European eels (*Anguilla anguilla*) in the Loire (France). Relationships with organic contaminant body burdens. *Science of the Total Environment*, **550**, 391-405.

Cozar, A., Echevarria, F., Gonzalez-Gordillo, J.I., Irigoien, X., Ubeda, B., Hernandez-Leon, S., Palma, A.T., Navarro, S., Garcia-de-Lomas, J., Ruiz, A., Fernandez-de-Puelles, M.L. & Duarte, C.M. (2014) Plastic debris in the open ocean. *Proceedings of the National Academy of Sciences*, **111**, 10239-44.

Crago, J., Bui, C., Grewal, S. & Schlenk, D. (2016) Age-dependent effects in fathead minnows from the anti-diabetic drug metformin. *General and Comparative Endocrinology*,

Creaser, C.S., Wood, M.D., Alcock, R., Copplestone, D., Crook, P.J. & Barraclough, D. (2007) Environmental concentrations of polychlorinated biphenyls (PCBs) in UK soil and herbage. In: *UK Soil and Herbage Pollutant Survey. UKSHS Report No. 8*. Environment Agency, Bristol.

Crews, D., Bergeron, J.M. & McLachlan, J.A. (1995) The role of estrogen in turtle sex determination and the effect of PCBs. *Environmental Health Perspectives*, **103**, 73-77.

Crofton, K.M., Craft, E.S., Hedge, J.M., Gennings, C., Simmons, J.E., Carchman, R.A., Carter, W.H., Jr. & DeVito, M.J. (2005) Thyroid-hormone-disrupting chemicals: evidence for dose-dependent additivity or synergism. *Environmental Health Perspectives*, **113**, 1549-54.

Custer, C.M., Custer, T.W., Thyen, S. & Becker, P.H. (2014) Incubation stage and polychlorinated biphenyl (PCB) congener patterns in an altricial and precocial bird species. *Environmental Pollution*, **195**, 109-114.

Custer, C.M., Custer, T.W., Schoenfuss, H.L., Poganski, B.H. & Solem, L. (2012) Exposure and effects of perfluoroalkyl compounds on tree swallows nesting at Lake Johanna in east central Minnesota, USA. *Reproductive Toxicology*, **33**, 556-562.

Custer, C.M., Custer, T.W., Rosiu, C.J., Melancon, M.J., Bickham, J.W. & Matson, C.W. (2005) Exposure and effects of 2,3,7,8-tetrachlorodibenzo-p-dioxin in tree swallows (*Tachycineta bicolor*) nesting along the Woonasquatucket River, Rhode Island, USA. *Environmental Toxicology and Chemistry*, **24**, 93-109.

Cuthbert, R., Green, R.E., Ranade, S., Saravanan, S., Pain, D.J., Prakash, V. & Cunningham, A.A. (2006) Rapid population declines of Egyptian vulture (*Neophron percnopterus*) and red-headed vulture (*Sarcogyps calvus*) in India. *Animal Conservation*, **9**, 349-354.

Cuthbert, R.J., Taggart, M.A., Prakash, V., Chakraborty, S.S., Deori, P., Galligan, T., Kulkarni, M., Ranade, S., Saini, M., Sharma, A.K., Shringarpure, R. & Green, R.E. (2014) Avian scavengers and the threat from veterinary pharmaceuticals. *Philosophical Transactions of the Royal Society of London B - Biological Sciences*, **369**

D'Mello, S.R., Cruz, C.N., Chen, M.L., Kapoor, M., Lee, S.L. & Tyner, K.M. (2017) The evolving landscape of drug products containing nanomaterials in the United States. *Nature Nanotechnology*, **12**, 523-529.

Dadson, S.J., Hall, J.W., Murgatroyd, A., Acreman, M., Bates, P., Beven, K., Heathwaite, L., Holden, J., Holman, I.P., Lane, S.N., O'Connell, E., Penning-Rowsell, E., Reynard, N., Sear, D., Thorne, C. & Wilby, R. (2017) A restatement of the natural science evidence concerning catchment-based ‘natural’ flood management in the UK. *Proceedings of the Royal Society A*, **473**, 20160706.

Dalton, R. (2010) E-mails spark ethics row. *Nature*, **466**, 913.

Damstra, T., Barlow, S., Bergman, A., Kavlock, R. & Kraak, G.V.D. (2002) Global assessment of the state-of-the-science of endocrine disruptors. In. World Health Organization

Dang, Z., Li, K., Yin, H., Hakkert, B. & Vermeire, T. (2011) Endpoint sensitivity in fish endocrine disruption assays: regulatory implications. *Toxicology Letters*, **202**, 36-46.

Darbre, P.D. (2015) *Endocrine disruption and human heath*. Elsevier, London.

Darling, E.S. & Côte, I.M. (2008) Quantifying the evidence for ecological synergies. *Ecology Letters*, **11**, 1278-86.

Das, K., Vossen, A., Tolley, K., Vikingsson, G., Thron, K., Mueller, G., Baumgaertner, W. & Siebert, U. (2006) Interfollicular fibrosis in the thyroid of the harbour porpoise: An endocrine disruption? *Archives of Environmental Contamination and Toxicology*, **51**, 720-729.

Daughton, C.G. (2014) The Matthew Effect and widely prescribed pharmaceuticals lacking environmental monitoring: case study of an exposure-assessment vulnerability. *Science of the Total Environment*, **466-467**, 315-25.

Daughton, C.G. & Ternes, T.A. (1999) Pharmaceuticals and personal care products in the environment: agents of subtle change? *Environmental Health Perspectives*, **107 Suppl 6**, 907-38.

Daughton, C.G. & Ruhoy, I.S. (2011) Green pharmacy and pharmEcovigilance: prescribing and the planet. *Expert Review of Clinical Pharmacology*, **4**, 211-32.

David, A., Fenet, H. & Gomez, E. (2009) Alkylphenols in marine environments: distribution monitoring strategies and detection considerations. *Marine Pollution Bulletin*, **58**, 953-60.

Davis, E.F., Klosterhaus, S.L. & Stapleton, H.M. (2012) Measurement of flame retardants and triclosan in municipal sewage sludge and biosolids. *Environment International*, **40**, 1-7.

de Mora, S.J., Stewart, C. & Phillips, D. (1995) Sources and rate of degradation of tri(n-butyl)tin in marine sediments near Auckland, New Zealand. *Marine Pollution Bulletin*, **30**, 50-57.

Dean, K.E., Palachek, R.M., Noel, J.M., Warbritton, R., Aufderheide, J. & Wireman, J. (2004) Development of Freshwater Water-Quality Criteria for Perchlorate. *Environmental Toxicology and Chemistry*, **23**, 1441.

Defra (2012) *Waste water treatment in the United Kingdom – 2012: Implementation of the European Union Urban Waste Water Treatment Directive – 91/271/EEC*. <https://www.gov.uk/government/uploads/system/uploads/attachment_data/file/69592/pb13811-waste-water-2012.pdf>, London, UK.

der Beek, T.A., Weber, F.-A., Bergmann, A., Hickmann, S., Ebert, I., Hein, A. & Kuester, A. (2016) Pharmaceuticals in the environment - global occurences and perspectives. *Environmental Toxicology and Chemistry*, **35**, 823-835.

Derocher, A.E., Wolkers, H., Colborn, T., Schlabach, M., Larsen, T.S. & Wiig, Ø. (2003) Contaminants in Svalbard polar bear samples archived since 1967 and possible population level effects. *Science of The Total Environment*, **301**, 163-174.

Desforges, J.P., Hall, A., McConnell, B., Rosing-Asvid, A., Barber, J.L., Brownlow, A., De Guise, S., Eulaers, I., Jepson, P.D., Letcher, R.J., Levin, M., Ross, P.S., Samarra, F., Vikingson, G., Sonne, C. & Dietz, R. (2018) Predicting global killer whale population collapse from PCB pollution. *Science*, **361**, 1373-1376.

Die, Q.Q., Nie, Z.Q., Yang, Y.F., Tang, Z.W. & Huang, Q.F. (2015) Persistent organic pollutant waste in China: a review of past experiences and future challenges. *Journal of Material Cycles and Waste Management*, **17**, 434-441.

Dietz, R., Riget, F., Cleemann, M., Aarkrog, A., Johansen, P. & Hansen, J. (2000) Comparison of contaminants from different trophic levels and ecosystems. *The Science of The Total Environment*, **245**, 221-231.

Dietz, R., Riget, F.F., Sonne, C., Born, E.W., Bechshoft, T., McKinney, M.A. & Letcher, R.J. (2013) Three decades (1983-2010) of contaminant trends in East Greenland polar bears (*Ursus maritimus*). Part 1: legacy organochlorine contaminants. *Environment International*, **59**, 485-93.

Dods, P.L., Birmingham, E.M., Williams, T.D., Ikonomou, M.G., Bennie, D.T. & Elliott, J.E. (2005) Reproductive success and contaminants in tree swallows (*Tachycineta bicolor*) breeding at a wastewater treatment plant. *Environmental Toxicology and Chemistry*, **24**, 3106-3112.

Dodson, R.E., Perovich, L.J., Covaci, A., Van den Eede, N., Ionas, A.C., Dirtu, A.C., Brody, J.G. & Rudel, R.A. (2012) After the PBDE phase-out: a broad suite of flame retardants in repeat house dust samples from California. *Environmental Science & Technology*, **46**, 13056-66.

Dowson, P.H., Bubb, J.M. & Lester, J.N. (1996) Persistence and Degradation Pathways of Tributyltin in Freshwater and Estuarine Sediments. *Estuarine, Coastal and Shelf Science*, **42**, 551-562.

Du, Y., Shi, X., Liu, C., Yu, K. & Zhou, B. (2009) Chronic effects of water-borne PFOS exposure on growth, survival and hepatotoxicity in zebrafish: a partial life-cycle test. *Chemosphere*, **74**, 723-9.

Dulio, V., van Bavel, B., Brorstrom-Lunden, E., Harmsen, J., Hollender, J., Schlabach, M., Slobodnik, J., Thomas, K. & Koschorreck, J. (2018) Emerging pollutants in the EU: 10 years of NORMAN in support of environmental policies and regulations. *Environmental Sciences Europe*, **30**, 5.

EFSA (2015) Scientific Opinion on the risks to public health related to the presence of bisphenol A (BPA) in foodstuffs: Part I – Exposure assessment and Part II Toxicological assessment and risk characterisation. *EFSA Journal*, **13**, 3978.

Eisenreich, K.M., Dean, K.M., Ottinger, M.A. & Rowe, C.L. (2012) Comparative effects of *in ovo* exposure to sodium perchlorate on development, growth, metabolism, and thyroid function in the common snapping turtle (*Chelydra serpentina*) and red-eared slider (*Trachemys scripta elegans*). *Comparative Biochemistry and Physiology - Part C: Toxicology & Pharmacology*, **156**, 166-70.

Eladak, S., Grisin, T., Moison, D., Guerquin, M.J., N'Tumba-Byn, T., Pozzi-Gaudin, S., Benachi, A., Livera, G., Rouiller-Fabre, V. & Habert, R. (2015) A new chapter in the bisphenol A story: bisphenol S and bisphenol F are not safe alternatives to this compound. *Fertility and Sterility*, **103**, 11-21.

Elliott, J.E., Brogan, J., Lee, S.L., Drouillard, K.G. & Elliott, K.H. (2015) PBDEs and other POPs in urban birds of prey partly explained by trophic level and carbon source. *Science of the Total Environment*, **524**, 157-165.

Eng, M.L., Elliott, J.E., MacDougall-Shackleton, S.A., Letcher, R.J. & Williams, T.D. (2012) Early exposure to 2,2',4,4',5-pentabromodiphenyl ether (BDE-99) affects mating behavior of zebra finches. *Toxicological Sciences*, **127**, 269-76.

Eriksson, U., Roos, A., Lind, Y., Hope, K., Ekblad, A. & Karrman, A. (2016) Comparison of PFASs contamination in the freshwater and terrestrial environments by analysis of eggs from osprey (*Pandion haliaetus*), tawny owl (*Strix aluco*), and common kestrel (*Falco tinnunculus*). *Environmental Research*, **149**, 40-47.

Erikstad, K.E., Sandvik, H., Reiertsen, T.K., Bustnes, J.O. & Strom, H. (2013) Persistent organic pollution in a high-Arctic top predator: sex-dependent thresholds in adult survival. *Proceedings of the Royal Society B-Biological Sciences*, **280**

Espin, S., Garcia-Fernandez, A.J., Herzke, D., Shore, R.F., van Hattum, B., Martinez-Lopez, E., Coeurdassier, M., Eulaers, I., Fritsch, C., Gomez-Ramirez, P., Jaspers, V.L., Krone, O., Duke, G., Helander, B., Mateo, R., Movalli, P., Sonne, C. & van den Brink, N.W. (2016) Tracking pan-continental trends in environmental contamination using sentinel raptors-what types of samples should we use? *Ecotoxicology*, **25**, 777-801.

Etheridge, M.L., Campbell, S.A., Erdman, A.G., Haynes, C.L., Wolf, S.M. & McCullough, J. (2013) The big picture on nanomedicine: the state of investigational and approved nanomedicine products. *Nanomedicine*, **9**, 1-14.

European Chemicals Agency (2015) *Candidate List of substances of very high concern for Authorisation*. <http://echa.europa.eu/candidate-list-table>.

European Chemicals Agency (2017) *Home*. <https://echa.europa.eu/>.

European Chemicals Agency (2018) *Guidance on Information Requirements and Chemical Safety Assessment*, <https://echa.europa.eu/guidance-documents/guidance-on-information-requirements-and-chemical-safety-assessment>.

European Commission (2003) Review report for the active substance atrazine. Finalised in the Standing Committee on the Food Chain and Animal Health at its meeting on 03 October 2003 in view of the inclusion of atrazine in Annex I of Council Directive 91/414/EEC. SANCO/10496/2003-Final, 10 September 2003, 4 pp.

European Commission (2016a) *The EU Water Framework Directive - integrated river basin management for Europe*. <http://ec.europa.eu/environment/water/water-framework/index_en.html>.

European Commission (2016b) *Press release: Commission presents scientific criteria to identify endocrine disruptors in the pesticides and biocides areas.* <http://europa.eu/rapid/press-release_IP-16-2152_en.htm>.

European Commission (2016c) *Endocrine disruptors*. <http://ec.europa.eu/environment/chemicals/endocrine/index_en.htm>.

European Commission (2017a) *Legislation*. <https://ec.europa.eu/health/endocrine_disruptors/legislation_en>.

European Commission (2017b) *Hormones in Meat*. <https://ec.europa.eu/food/safety/chemical_safety/meat_hormones_en>.

European Cooperation in Science and Technology (2018) *European Raptor Biomonitoring Facility*. <http://www.cost.eu/COST_Actions/ca/CA16224>.

European Council for Plasticisers and Intermediates (2014) *Plasticisers & Flexible PVC Information Centre*. <http://www.plasticisers.org>.

European Food Safety Authority (2016) *Endocrine Active Substances*. Available at: (accessed

Evans, S.M. (1999) Tributyltin Pollution: the Catastrophe that Never Happened. *Marine Pollution Bulletin*, **38**, 629-636.

Fair, P.A., Adams, J., Mitchum, G., Hulsey, T.C., Reif, J.S., Houde, M., Muir, D., Wirth, E., Wetzel, D., Zolman, E., McFee, W. & Bossart, G.D. (2010) Contaminant blubber burdens in Atlantic bottlenose dolphins (*Tursiops truncatus*) from two southeastern US estuarine areas: Concentrations and patterns of PCBs, pesticides, PBDEs, PFCs, and PAHs. *Science of the Total Environment*, **408**, 1577-1597.

Fedak, K.M., Bernal, A., Capshaw, Z.A. & Gross, S. (2015) Applying the Bradford Hill criteria in the 21st century: how data integration has changed causal inference in molecular epidemiology. *Emerging Themes in Epidemiology*, **12**, 14.

Feist, G.W., Webb, M.A.H., Gundersen, D.T., Foster, E.P., Schreck, C.B., Maule, A.G. & Fitzpatrick, M.S. (2005) Evidence of detrimental effects of environmental contaminants on growth and reproductive physiology of white sturgeon in impounded areas of the Columbia River. *Environmental Health Perspectives*, **113**, 1675-1682.

Felton, R.G., Steiner, C.C., Durrant, B.S., Keisler, D.H., Milnes, M.R. & Tubbs, C.W. (2015) Identification of California Condor Estrogen Receptors 1 and 2 and Their Activation by Endocrine Disrupting Chemicals. *Endocrinology*, **156**, 4448-57.

Fenichel, P., Brucker-Davis, F. & Chevalier, N. (2015) The history of Distilbene(R) (Diethylstilbestrol) told to grandchildren -- the transgenerational effect. *Annales d’Endocrinologie*, **76**, 253-9.

Fenner, K., Scheringer, M., MacLeod, M., Matthies, M., McKone, T., Stroebe, M., Beyer, A., Bonnell, M., Le Gall, A.C., Klasmeier, J., Mackay, D., van de Meent, D., Pennington, D., Scharenberg, B., Suzuki, N. & Wania, F. (2005) Comparing Estimates of Persistence and Long-Range Transport Potential among Multimedia Models. *Environmental Science & Technology*, **39**, 1932-1942.

Fent, K. (1996) Ecotoxicology of Organotin Compounds. *Critical Reviews in Toxicology*, **26**, 3-117.

Fent, K. (2015) Progestins as endocrine disrupters in aquatic ecosystems: Concentrations, effects and risk assessment. *Environment International*, **84**, 115-30.

Fernie, K.J., Shutt, J.L., Letcher, R.J., Ritchie, J.I., Sullivan, K. & Bird, D.M. (2008) Changes in reproductive courtship behaviors of adult American kestrels (*Falco sparverius*) exposed to environmentally relevant levels of the polybrominated diphenyl ether mixture, DE-71. *Toxicological Sciences*, **102**, 171-8.

Flint, S., Markle, T., Thompson, S. & Wallace, E. (2012) Bisphenol A exposure, effects, and policy: A wildlife perspective. *Journal of Environmental Management*, **104**, 19-34.

Folland, W.R., Newsted, J.L., Fitzgerald, S.D., Fuchsman, P.C., Bradley, P.W., Kern, J., Kannan, K., Remington, R.E. & Zwiernik, M.J. (2016) Growth and reproductive effects from dietary exposure to Aroclor 1268 in mink (*Neovison vison*), a surrogate model for marine mammals. *Environmental Toxicology and Chemistry*, **35**, 604-18.

Foran, C.M., Peterson, B.N. & Benson, W.H. (2002) Transgenerational and developmental exposure of Japanese medaka (*Oryzias latipes*) to ethinylestradiol results in endocrine and reproductive differences in the response to ethinylestradiol as adults. *Toxicological Sciences*, **68**, 389-402.

Forsgren, K.L., Qu, S., Lavado, R., Cwiertny, D. & Schlenk, D. (2014) Trenbolone acetate metabolites promote ovarian growth and development in adult Japanese medaka (*Oryzias latipes*). *General and Comparative Endocrinology*, **202**, 1-7.

Fox, G.A. (1991) Practical causal inference for ecoepidemiologists. *Journal of Toxicology and Environmental Health*, **33**, 359-73.

Fox, G.A., Lundberg, R., Wejheden, C., Lind, L., Larsson, S., Orberg, J. & Lind, P.M. (2008) Health of herring gulls (*Larus argentatus*) in relation to breeding location in the early 1990s. III. Effects on the bone tissue. *Journal of Toxicology and Environmental Health A*, **71**, 1448-56.

Frank, L.G., Jackson, R.M., Cooper, J.E. & French, M.C. (1977) A survey of chlorinated hydrocarbon residues in Kenyan birds of prey. *African Journal of Ecology*, **15**, 295-304.

Frazzoli, C., Orisakwe, O.E., Dragone, R. & Mantovani, A. (2010) Diagnostic health risk assessment of electronic waste on the general population in developing countries' scenarios. *Environmental Impact Assessment Review*, **30**, 388-399.

Frederiksen, M., Meyer, H.W., Ebbehoj, N.E. & Gunnarsen, L. (2012) Polychlorinated biphenyls (PCBs) in indoor air originating from sealants in contaminated and uncontaminated apartments within the same housing estate. *Chemosphere*, **89**, 473-9.

French Jr, J.B., Nisbet, I.C.T. & Schwabl, H. (2001) Maternal steroids and contaminants in common tern eggs: a mechanism of endocrine disruption? *Comparative Biochemistry and Physiology Part C: Toxicology & Pharmacology*, **128**, 91-98.

Friedman, O., Arad, E. & Ben Amotz, O. (2016) Body Builder's Nightmare: Black Market Steroid Injection Gone Wrong: a Case Report. *Plastic and Reconstructive Surgery-Global Open*, **4**

Fromme, H., Kuchler, T., Otto, T., Pilz, K., Muller, J. & Wenzel, A. (2002) Occurrence of phthalates and bisphenol A and F in the environment. *Water Research*, **36**, 1429-38.

Frouin, H., Lebeuf, M., Hammill, M., Sjare, B. & Fournier, M. (2011) PBDEs in serum and blubber of harbor, grey and harp seal pups from Eastern Canada. *Chemosphere*, **82**, 663-669.

Frouin, H., Lebeuf, M., Saint-Louis, R., Hammill, M., Pelletier, E. & Fournier, M. (2008) Toxic effects of tributyltin and its metabolites on harbour seal (*Phoca vitulina*) immune cells in vitro. *Aquatic Toxicology*, **90**, 243-251.

Fu, J., Wang, Y., Zhang, A., Zhang, Q., Zhao, Z., Wang, T. & Jiang, G. (2011) Spatial distribution of polychlorinated biphenyls (PCBs) and polybrominated biphenyl ethers (PBDEs) in an e-waste dismantling region in Southeast China: Use of apple snail (Ampullariidae) as a bioindicator. *Chemosphere*, **82**, 648-655.

Furin, C.G., von Hippel, F.A., Postlethwait, J., Buck, C.L., Cresko, W.A. & O'Hara, T.M. (2015) Developmental timing of perchlorate exposure alters threespine stickleback dermal bone. *General and Comparative Endocrinology*, **219**, 36-44.

Futran Fuhrman, V., Tal, A. & Arnon, S. (2015) Why endocrine disrupting chemicals (EDCs) challenge traditional risk assessment and how to respond. *Journal of Hazardous Materials*, **286**, 589-611.

Gabrielsen, K.M., Villanger, G.D., Lie, E., Karimi, M., Lydersen, C., Kovacs, K.M. & Jenssen, B.M. (2011) Levels and patterns of hydroxylated polychlorinated biphenyls (OH-PCBs) and their associations with thyroid hormones in hooded seal (*Cystophora cristata*) mother-pup pairs. *Aquatic Toxicology*, **105**, 482-491.

Galante-Oliveira, S., Oliveira, I., Jonkers, N., Langston, W.J., Pacheco, M. & Barroso, C.M. (2009) Imposex levels and tributyltin pollution in Ria de Aveiro (NW Portugal) between 1997 and 2007: evaluation of legislation effectiveness. *Journal of Environmental Monitoring*, **11**, 1405-11.

Galligan, T.H., Amano, T., Prakash, V.M., Kulkarni, M., Shringarpure, R., Prakash, N., Ranade, S., Green, R.E. & Cuthbert, R.J. (2014) Have population declines in Egyptian Vulture and Red-headed Vulture in India slowed since the 2006 ban on veterinary diclofenac? *Bird Conservation International*, **24**, 272-281.

Gao, D.W. & Wen, Z.D. (2016) Phthalate esters in the environment: A critical review of their occurrence, biodegradation, and removal during wastewater treatment processes. *Science of the Total Environment*, **541**, 986-1001.

Gardner, M., Comber, S., Scrimshaw, M.D., Cartmell, E., Lester, J. & Ellor, B. (2012) The significance of hazardous chemicals in wastewater treatment works effluents. *Science of the Total Environment*, **437**, 363-72.

Gardner, M., Jones, V., Comber, S., Scrimshaw, M.D., Coello-Garcia, T., Cartmell, E., Lester, J. & Ellor, B. (2013) Performance of UK wastewater treatment works with respect to trace contaminants. *Science of the Total Environment*, **456-457**, 359-69.

Gaylor, M.O., Harvey, E. & Hale, R.C. (2012) House crickets can accumulate polybrominated diphenyl ethers (PBDEs) directly from polyurethane foam common in consumer products. *Chemosphere*, **86**, 500-505.

Gazo, I., Linhartova, P., Shaliutina, A. & Hulak, M. (2013) Influence of environmentally relevant concentrations of vinclozolin on quality, DNA integrity, and antioxidant responses of sterlet *Acipenser ruthenus* spermatozoa. *Chemico-Biological Interactions*, **203**, 377-385.

Gebbink, W.A., Bossi, R., Riget, F.F., Rosing-Asvid, A., Sonne, C. & Dietz, R. (2016) Observation of emerging per- and polyfluoroalkyl substances (PFASs) in Greenland marine mammals. *Chemosphere*, **144**, 2384-2391.

Gee, D. (2013) More or less precaution? *Late lessons from early warnings: science, precaution, innovation* (ed. by D. Gee). EEA Report No 1/2013, European Environment Agency, Copenhagen.

Geisz, H.N., Dickhut, R.M., Cochran, M.A., Fraser, W.R. & Ducklow, H.W. (2008) Melting Glaciers: A Probable Source of DDT to the Antarctic Marine Ecosystem. *Environmental Science & Technology*, **42**, 3958-3962.

Genuis, S.J., Beesoon, S., Birkholz, D. & Lobo, R.A. (2012) Human excretion of bisphenol A: blood, urine, and sweat (BUS) study. *J Environ Public Health*, **2012**, 185731.

George, J.L. & Frear, D.E.H. (1966) Pesticides in the Antarctic. *Journal of Applied Ecology*, **3**, 155.

Georgescu, B., Georgescu, C., Dărăban, S., Bouaru, A. & Paşcalău, S. (2011) Heavy Metals Acting as Endocrine Disrupters. *Animal Science and Biotechnologies*, **44**, 89-93.

Geyer, R., Jambeck, J.R. & Law, K.L. (2017) Production, use, and fate of all plastics ever made. *Science Advances*, **3**, e1700782.

Ghoshdastidar, A.J., Fox, S. & Tong, A.Z. (2015) The presence of the top prescribed pharmaceuticals in treated sewage effluents and receiving waters in Southwest Nova Scotia, Canada. *Environmental Science and Pollution Research*, **22**, 689-700.

Gibbs, P.E. & Bryan, G.W. (1986) Reproductive Failure in Populations of the Dog-Whelk, *Nucella lapillus*, Caused by Imposex Induced by Tributyltin from Antifouling Paints. *Journal of the Marine Biological Association of the United Kingdom*, **66**, 767-777.

Gies, A. & Soto, A.M. (2013) Bisphenol A: contested science, divergent safety evaluations. In: *Late lessons from early warnings: science, precaution, innovation. EEA Report No 1/2013* eds. D. Gee, P. Grandjean, S.F. Hansen, S. Van den hove, M. Macgarvin, J. Martin, G. Nielsen, D. Quist and D. Stanner). European Environmental Agency, Copenhagen.

Giesy, J.P. & Kannan, K. (2001) Global distribution of perfluorooctane sulfonate in wildlife. *Environmental Science & Technology*, **35**, 1339-1342.

Glennemeier, K.A. & Denver, R.J. (2001) Sublethal effects of chronic exposure to an organochlorine compound on northern leopard frog (*Rana pipiens*) tadpoles. *Environmental Toxicology*, **16**, 287-297.

Global Burden of Disease Study 2013 Collaborators (2015) Global, regional, and national incidence, prevalence, and years lived with disability for 301 acute and chronic diseases and injuries in 188 countries, 1990–2013: a systematic analysis for the Global Burden of Disease Study 2013. *The Lancet*, **386**, 743-800.

Godfray, H.C., Blacquiere, T., Field, L.M., Hails, R.S., Potts, S.G., Raine, N.E., Vanbergen, A.J. & McLean, A.R. (2015) A restatement of recent advances in the natural science evidence base concerning neonicotinoid insecticides and insect pollinators. *Proceedings of the Royal Society B*, **282**, 20151821.

Godfray, H.C., Blacquiere, T., Field, L.M., Hails, R.S., Petrokofsky, G., Potts, S.G., Raine, N.E., Vanbergen, A.J. & McLean, A.R. (2014) A restatement of the natural science evidence base concerning neonicotinoid insecticides and insect pollinators. *Proceedings of the Royal Society B*, **281**

Godfray, H.C., Donnelly, C.A., Kao, R.R., Macdonald, D.W., McDonald, R.A., Petrokofsky, G., Wood, J.L., Woodroffe, R., Young, D.B. & McLean, A.R. (2013) A restatement of the natural science evidence base relevant to the control of bovine tuberculosis in Great Britain. *Proceedings of the Royal Society B*, **280**, 20131634.

Goleman, W.L., Urquidi, L.J., Anderson, T.A., Smith, E.E., Kendall, R.J. & Carr, J.A. (2002) Environmentally relevant concentrations of ammonium perchlorate inhibit development and metamorphosis in *Xenopus laevis*. *Environmental Toxicology and Chemistry*, **21**, 424-430.

Gomes, H.I., Dias-Ferreira, C. & Ribeiro, A.B. (2013) Overview of in situ and ex situ remediation technologies for PCB-contaminated soils and sediments and obstacles for full-scale application. *Science of the Total Environment*, **445-446**, 237-60.

Gore, A.C., Chappell, V.A., Fenton, S.E., Flaws, J.A., Nadal, A., Prins, G.S., Toppari, J. & Zoeller, R.T. (2015) Executive Summary to EDC-2: The Endocrine Society's Second Scientific Statement on Endocrine-Disrupting Chemicals. *Endocrine Reviews*, **36**, 593-602.

Gorka, J., Taylor-Gjevre, R.M. & Arnason, T. (2013) Metabolic and clinical consequences of hyperthyroidism on bone density. *International Journal of Endocrinology*, **2013**, 638727.

Goutte, A., Barbraud, C., Meillere, A., Carravieri, A., Bustamante, P., Labadie, P., Budzinski, H., Delord, K., Cherel, Y., Weimerskirch, H. & Chastel, O. (2014) Demographic consequences of heavy metals and persistent organic pollutants in a vulnerable long-lived bird, the wandering albatross. *Proceedings of the Royal Society B*, **281**, 20133313.

Government Chemist and Environment Agency (2015) *Nonylphenol ethoxylates to be banned in imported clothing and textiles*. <https://www.gov.uk/government/news/nonylphenol-ethoxylates-to-be-banned-in-imported-clothing-and-textiles>.

Grant, K., Goldizen, F.C., Sly, P.D., Brune, M.-N., Neira, M., van den Berg, M. & Norman, R.E. (2013) Health consequences of exposure to e-waste: a systematic review. *The Lancet Global Health*, **1**, e350-e361.

Green, C., Brian, J., Kanda, R., Scholze, M., Williams, R. & Jobling, S. (2015) Environmental concentrations of anti-androgenic pharmaceuticals do not impact sexual disruption in fish alone or in combination with steroid oestrogens. *Aquatic Toxicology*, **160**, 117-127.

Green, J.M., Lange, A., Scott, A., Trznadel, M., Wai, H.A., Takesono, A., Brown, A.R., Owen, S.F., Kudoh, T. & Tyler, C.R. (2018) Early life exposure to ethinylestradiol enhances subsequent responses to environmental estrogens measured in a novel transgenic zebrafish. *Scientific Reports*, **8**, 2699.

Grier, J.W. (1982) Ban of DDT and subsequent recovery of reproduction in bald eagles. *Science*, **218**, 1232-5.

Grossman, C. (1985) Interactions between the gonadal steroids and the immune system. *Science*, **227**, 257-261.

Guerrero, L.A., Maas, G. & Hogland, W. (2013) Solid waste management challenges for cities in developing countries. *Waste Management*, **33**, 220-32.

Guglielmo, F., Lammel, G. & Maier-Reimer, E. (2009) Global environmental cycling of gamma-HCH and DDT in the 1980s--a study using a coupled atmosphere and ocean general circulation model. *Chemosphere*, **76**, 1509-17.

Gui, D., Yu, R., He, X., Tu, Q., Chen, L. & Wu, Y. (2014) Bioaccumulation and biomagnification of persistent organic pollutants in Indo-Pacific humpback dolphins (*Sousa chinensis*) from the Pearl River Estuary, China. *Chemosphere*, **114**, 106-13.

Guillette, L.J., Crain, D.A., Gunderson, M.P., Kools, S.A.E., Milnes, M.R., Orlando, E.F., Rooney, A.A. & Woodward, A.R. (2000) Alligators and endocrine disrupting contaminants: A current perspective. *American Zoologist*, **40**, 438-452.

Gunderson, M.P., Bermudez, D.S., Bryan, T.A., Degala, S., Edwards, T.M., Kools, S.A.E., Milnes, M.R., Woodward, A.R. & Guillette, L.J. (2004) Variation in sex steroids and phallus size in juvenile American alligators (*Alligator mississippiensis*) collected from 3 sites within the Kissimmee-Everglades drainage in Florida (USA). *Chemosphere*, **56**, 335-345.

Gurnell, M., Burrin, J. & Chatterjee, V.K. (2010) Principles of hormone action. In: *Oxford Textbook of Medicine* eds. D.A. Warrell, T.M. Cox and J.D. Firth), pp. 1787-1798. Oxford University Press

Gusev, A., MacLeod, M. & Bartlett, P. (2012) Intercontinental transport of persistent organic pollutants: a review of key findings and recommendations of the task force on hemispheric transport of air pollutants and directions for future research. *Atmospheric Pollution Research*, **3**, 463-465.

Hale, R.C., Kim, S.L., Harvey, E., La Guardia, M.J., Mainor, T.M., Bush, E.O. & Jacobs, E.M. (2008) Antarctic research bases: Local sources of polybrominated diphenlyl ether (PBDE) flame retardants. *Environmental Science & Technology*, **42**, 1452-1457.

Hall, A.J. & Thomas, G.O. (2007) Polychlorinated biphenyls, DDT, polybrominated diphenyl ethers, and organic pesticides in United Kingdom harbor seals (*Phoca vitulina*) - Mixed exposures and thyroid homeostasis. *Environmental Toxicology and Chemistry*, **26**, 851-861.

Hall, A.J., Kalantzi, O.I. & Thomas, G.O. (2003) Polybrominated diphenyl ethers (PBDEs) in grey seals during their first year of life - are they thyroid hormone endocrine disrupters? *Environmental Pollution*, **126**, 29-37.

Hall, A.J., McConnell, B.J., Rowles, T.K., Aguilar, A., Borrell, A., Schwacke, L., Reijnders, P.J.H. & Wells, R.S. (2006) Individual-Based Model Framework to Assess Population Consequences of Polychlorinated Biphenyl Exposure in Bottlenose Dolphins. *Environmental Health Perspectives*, **114**, 60-64.

Hallers-Tjabbes, C.C.T., Kemp, J.F. & Boon, J.P. (1994) Imposex in whelks (*Buccinum undatum*) from the open North Sea: Relation to shipping traffic intensities. *Marine Pollution Bulletin*, **28**, 311-313.

Hamilton, P.B., Lange, A., Nicol, E., Bickley, L.K., De-Bastos, E.S.R., Jobling, S. & Tyler, C.R. (2015) Effects of Exposure to WwTW Effluents over Two Generations on Sexual Development and Breeding in Roach *Rutilus rutilus*. *Environmental Science & Technology*, **49**, 12994-13002.

Hamilton, P.B., Nicol, E., De-Bastos, E.S.R., Williams, R.J., Sumpter, J.P., Jobling, S., Stevens, J.R. & Tyler, C.R. (2014) Populations of a cyprinid fish are self-sustaining despite widespread feminization of males. *BMC Biology*, **12**, 1.

Hammond, J.A., Hall, A.J. & Dyrynda, E.A. (2005) Comparison of polychlorinated biphenyl (PCB) induced effects on innate immune functions in harbour and grey seals. *Aquatic Toxicology*, **74**, 126-138.

Hampel, M., Alonso, E., Aparicio, I., Santos, J.L. & Leaver, M. (2015) Hepatic proteome analysis of Atlantic salmon (*Salmo salar*) after exposure to environmental concentrations of human pharmaceuticals. *Molecular and Cellular Proteomics*, **14**, 371-81.

Hansen, K.J., Clemen, L.A., Ellefson, M.E. & Johnson, H.O. (2001) Compound-specific, quantitative characterization of organic fluorochemicals in biological matrices. *Environ Sci Technol*, **35**, 766-70.

Hansen, K.M., Christensen, J.H., Geels, C., Silver, J.D. & Brandt, J. (2015) Modelling the impact of climate change on the atmospheric transport and the fate of persistent organic pollutants in the Arctic. *Atmospheric Chemistry and Physics*, **15**, 6549-6559.

Hanson, N., Åberg, P. & Sundelöf, A. (2005) Population-level effects of male-biased broods in eelpout (*Zoarces viviparus*). *Environmental Toxicology and Chemistry*, **24**, 1235-1241.

Harding, L.E., Harris, M.L., Stephen, C.R. & Elliott, J.E. (1999) Reproductive and Morphological Condition of Wild Mink (*Mustela vison*) and River Otters (*Lutra canadensis*) in Relation to Chlorinated Hydrocarbon Contamination. *Environmental Health Perspectives*, **107**, 141.

Harrad, S., Ibarra, C., Robson, M., Melymuk, L., Zhang, X., Diamond, M. & Douwes, J. (2009) Polychlorinated biphenyls in domestic dust from Canada, New Zealand, United Kingdom and United States: implications for human exposure. *Chemosphere*, **76**, 232-8.

Harrad, S.J., Sewart, A.P., Alcock, R., Boumphrey, R., Burnett, V., Duarte-Davidson, R., Halsall, C., Sanders, G., Waterhouse, K., Wild, S.R. & Jones, K.C. (1994) Polychlorinated biphenyls (PCBs) in the British environment: Sinks, sources and temporal trends. *Environmental Pollution*, **85**, 131-146.

Harries, J.E., Sheahan, D.A., Jobling, S., Matthiessen, P., Neall, P., Sumpter, J.P., Tylor, T. & Zaman, N. (1997) Estrogenic activity in five United Kingdom rivers detected by measurement of vitellogenesis in caged male trout. *Environmental Toxicology and Chemistry*, **16**, 534-542.

Harris, C.A., Henttu, P., Parker, M.G. & Sumpter, J.P. (1997) The estrogenic activity of phthalate esters in vitro. *Environmental Health Perspectives*, **105**, 802-811.

Harris, C.A., Hamilton, P.B., Runnalls, T.J., Vinciotti, V., Henshaw, A., Hodgson, D., Coe, T.S., Jobling, S., Tyler, C.R. & Sumpter, J.P. (2011) The Consequences of Feminization in Breeding Groups of Wild Fish. *Environmental Health Perspectives*, **119**, 306-311.

Hartung, T. (2009) Toxicology for the twenty-first century. *Nature*, **460**, 208-12.

Haselman, J.T., Kosian, P.A., Korte, J.J., Olmstead, A.W., Iguchi, T., Johnson, R.D. & Degitz, S.J. (2016) Development of the Larval Amphibian Growth and Development Assay: effects of chronic 4-tert-octylphenol or 17 beta-trenbolone exposure in *Xenopus laevis* from embryo to juvenile. *Journal of Applied Toxicology*, **36**, 1639-1650.

Hatzinger, P.B. (2005) Perchlorate Biodegradation for Water Treatment. *Environmental Science & Technology*, **39**, 239A-247A.

Haukas, M., Berger, U., Hop, H., Gulliksen, B. & Gabrielsen, G.W. (2007) Bioaccumulation of per- and polyfluorinated alkyl substances (PFAS) in selected species from the Barents Sea food web. *Environmental Pollution*, **148**, 360-371.

Hauser, R. & Calafat, A.M. (2005) Phthalates and human health. *Occupational and Environmental Medicine*, **62**, 806-18.

Hayes, T. (2005) Comment on "Gonadal development of larval male *Xenopus laevis* exposed to atrazine in outdoor microcosms". *Environmental Science & Technology*, **39**, 7757-7758.

Hayes, T., Haston, K., Tsui, M., Hoang, A., Haeffele, C. & Vonk, A. (2002a) Herbicides: feminization of male frogs in the wild. *Nature*, **419**, 895-6.

Hayes, T., Haston, K., Tsui, M., Hoang, A., Haeffele, C. & Vonk, A. (2003) Atrazine-induced hermaphroditism at 0.1 ppb in American leopard frogs (*Rana pipiens*): Laboratory and field evidence. *Environmental Health Perspectives*, **111**, 568-575.

Hayes, T.B. (2004) There is no denying this: Defusing the confusion about atrazine. *Bioscience*, **54**, 1138-1149.

Hayes, T.B., Collins, A., Lee, M., Mendoza, M., Noriega, N., Stuart, A.A. & Vonk, A. (2002b) Hermaphroditic, demasculinized frogs after exposure to the herbicide atrazine at low ecologically relevant doses. *Proceedings of the National Academy of Sciences*, **99**, 5476-80.

Hayes, T.B., Stuart, A.A., Mendoza, M., Collins, A., Noriega, N., Vonk, A., Johnston, G., Liu, R. & Kpodzo, D. (2006) Characterization of Atrazine-Induced Gonadal Malformations in African Clawed Frogs (*Xenopus laevis*) and Comparisons with Effects of an Androgen Antagonist (Cyproterone Acetate) and Exogenous Estrogen (17β-Estradiol): Support for the Demasculinization/Feminization Hypothesis. *Environmental Health Perspectives*, **114**, 134-141.

Hayes, T.B., Khoury, V., Narayan, A., Nazir, M., Park, A., Brown, T., Adame, L., Chan, E., Buchholz, D., Stueve, T. & Gallipeau, S. (2010) Atrazine induces complete feminization and chemical castration in male African clawed frogs (*Xenopus laevis*). *Proceedings of the National Academy of Sciences of the United States of America*, **107**, 4612-4617.

HBM4EU (2017) *Coordinating and advancing human biomonitoring in Europe to provide evidence for chemical policy making*. <https://www.hbm4eu.eu/>.

Healy, B.F., English, K.R., Jagals, P. & Sly, P.D. (2015) Bisphenol A exposure pathways in early childhood: Reviewing the need for improved risk assessment models. *Journal of Exposure Science and Environmental Epidemiology*, **25**, 544-56.

Heintz, M.M., Brander, S.M. & White, J.W. (2015) Endocrine Disrupting Compounds Alter Risk-Taking Behavior in Guppies (*Poecilia reticulata*). *Ethology*, **121**, 480-491.

Henning, M.H., Robinson, S.K., McKay, K.J., Sullivan, J.P. & Bruckert, H. (2003) Productivity of American robins exposed to polychlorinated biphenyls, Housatonic River, Massachusetts, USA. *Environmental Toxicology and Chemistry*, **22**, 2783-2788.

Henny, C.J., Grove, R.A., Kaiser, J.L. & Johnson, B.L. (2010) North American Osprey Populations and Contaminants: Historic and Contemporary Perspectives. *Journal of Toxicology and Environmental Health-Part B-Critical Reviews*, **13**, 579-603.

Henriksen, E.O., Wiig, Ø., Skaare, J.U., Gabrielsen, G.W. & Derocher, A.E. (2001) Monitoring PCBs in polar bears: lessons learned from Svalbard. *Journal of Environmental Monitoring*, **3**, 493-498.

Herrick, R.F., Stewart, J.H. & Allen, J.G. (2016) Review of PCBs in US schools: a brief history, an estimate of the number of impacted schools, and an approach for evaluating indoor air samples. *Environmental Science and Pollution Research*, **23**, 1975-85.

Hertz, Y., Epstein, N., Abraham, M., Madar, Z., Hepher, B. & Gertler, A. (1989) Effects of metformin on plasma-insulin, glucose-metabolism and protein-synthesis in the common carp (*Cyprinus carpio* L.). *Aquaculture*, **80**, 175-187.

Hicks, K.A., Fuzzen, M.L., McCann, E.K., Arlos, M.J., Bragg, L.M., Kleywegt, S., Tetreault, G.R., McMaster, M.E. & Servos, M.R. (2017) Reduction of Intersex in a Wild Fish Population in Response to Major Municipal Wastewater Treatment Plant Upgrades. *Environmental Science & Technology*, **51**, 1811-1819.

Hirabayashi, Y. & Inoue, T. (2011) The low-dose issue and stochastic responses to endocrine disruptors. *Journal of Applied Toxicology*, **31**, 84-88.

Hoch, M. (2001) Organotin compounds in the environment - an overview. *Applied Geochemistry*, **16**, 719-743.

Holm, L., Blomqvist, A., Brandt, I., Brunstrom, B., Ridderstrale, Y. & Berg, C. (2006) Embryonic exposure to *o,p*'-DDT causes eggshell thinning and altered shell gland carbonic anhydrase expression in the domestic hen. *Environmental Toxicology and Chemistry*, **25**, 2787-93.

Holmstrom, K.E., Johansson, A.-K., Bignert, A., Lindberg, P. & Berger, U. (2010) Temporal Trends of Perfluorinated Surfactants in Swedish Peregrine Falcon Eggs (*Falco peregrinus*), 1974-2007. *Environmental Science & Technology*, **44**, 4083-4088.

Horiguchi, T., Li, Z., Uno, S., Shimizu, M., Shiraishi, H., Morita, M., Thompson, J.A.J. & Levings, C.D. (2004) Contamination of organotin compounds and imposex in molluscs from Vancouver, Canada. *Marine Environmental Research*, **57**, 75-88.

Horiguchi, T., Takiguchi, N., Cho, H.S., Kojima, M., Kaya, M., Shiraishi, H., Morita, M., Hirose, H. & Shimizu, M. (2000) Ovo-testis and disturbed reproductive cycle in the giant abalone, Haliotis madaka: possible linkage with organotin contamination in a site of population decline. *Marine Environmental Research*, **50**, 223-229.

Hornshaw, T.C., Aulerich, R.J. & Johnson, H.E. (1983) Feeding Great Lakes fish to mink: effects on mink and accumulation and elimination of PCBS by mink. *Journal of Toxicology and Environmental Health*, **11**, 933-46.

Houde, M., De Silva, A.O., Muir, D.C.G. & Letcher, R.J. (2011) Monitoring of Perfluorinated Compounds in Aquatic Biota: An Updated Review. *Environmental Science & Technology*, **45**, 7962-7973.

Houde, M., Martin, J.W., Letcher, R.J., Solomon, K.R. & Muir, D.C.G. (2006) Biological Monitoring of Polyfluoroalkyl Substances:  A Review. *Environmental Science & Technology*, **40**, 3463-3473.

Howard, C.V. (2013) Pest Control: Syngenta’s Secret Campaign to Discredit Atrazine’s Critics. In: *100Reporters and Environmental Health News*

Hu, J., Zhang, Z., Wei, Q., Zhen, H., Zhao, Y., Peng, H., Wan, Y., Giesy, J.P., Li, L. & Zhang, B. (2009) Malformations of the endangered Chinese sturgeon, *Acipenser sinensis*, and its causal agent. *Proceedings of the National Academy of Sciences*, **106**, 9339-9344.

Huang, R., Xia, M., Sakamuru, S., Zhao, J., Shahane, S.A., Attene-Ramos, M., Zhao, T., Austin, C.P. & Simeonov, A. (2016) Modelling the Tox21 10 K chemical profiles fo*r in vivo* toxicity prediction and mechanism characterization. *Nature Communications*, **7**, 10425.

Huber, S., Warner, N.A., Nygard, T., Remberger, M., Harju, M., Uggerud, H.T., Kaj, L. & Hanssen, L. (2015) A broad cocktail of environmental pollutants found in eggs of three seabird species from remote colonies in Norway. *Environmental Toxicology and Chemistry*, **34**, 1296-1308.

Hung, H., Katsoyiannis, A.A. & Guardans, R. (2016) Ten years of global monitoring under the Stockholm Convention on Persistent Organic Pollutants (POPs): Trends, sources and transport modelling. *Environmental Pollution*, **217**, 1-3.

Hutchinson, T.H., Ankley, G.T., Segner, H. & Tyler, C.R. (2005) Screening and Testing for Endocrine Disruption in Fish—Biomarkers As “Signposts,” Not “Traffic Lights,” in Risk Assessment. *Environmental Health Perspectives*, **114**, 106-114.

Im, J. & Loffler, F.E. (2016) Fate of Bisphenol A in Terrestrial and Aquatic Environments. *Environmental Science & Technology*, **50**, 8403-8416.

International Maritime Organisation (2002) *Anti-fouling systems*. <http://www.imo.org/en/OurWork/Environment/Anti-foulingSystems/Pages/Default.aspx>.

International Programme on Chemical Safety (2009) Chapter 5: Dose-response assessment and derivation of health-based guidance values. *Environmental Health Criteria 240: Principles and Methods for the Risk Assessment of Chemicals in Food* (ed. by Food and agriculture organization of the united nations and the world health organization).

Jaacks, L.M. & Prasad, S. (2017) The ecological cost of continued use of endocrine-disrupting chemicals. *The Lancet Diabetes & Endocrinology*, **5**, 14-15.

Jacobs, G.R., Gundersen, D.T., Webb, M.A.H., Gorsky, D., Kohl, K. & Lockwood, K. (2014) Evaluation of Organochlorine Pesticides and Sex Steroids in Lower Niagara River Lake Sturgeon. *Journal of Fish and Wildlife Management*, **5**, 109-117.

Jamieson, A.J., Malkocs, T., Piertney, S.B., Fujii, T. & Zhang, Z. (2017) Bioaccumulation of persistent organic pollutants in the deepest ocean fauna. *Nature Ecology and Evolution*, **1**, 51.

Jan, M.R., Shah, J., Khawaja, M.A. & Gul, K. (2009) DDT residue in soil and water in and around abandoned DDT manufacturing factory. *Environmental Monitoring and Assessment*, **155**, 31-8.

Jandegian, C.M., Deem, S.L., Bhandari, R.K., Holliday, C.M., Nicks, D., Rosenfeld, C.S., Selcer, K.W., Tillitt, D.E., vom Saal, F.S., Velez-Rivera, V., Yang, Y. & Holliday, D.K. (2015) Developmental exposure to bisphenol A (BPA) alters sexual differentiation in painted turtles (*Chrysemys picta*). *General and Comparative Endocrinology*, **216**, 77-85.

Jara-Carrasco, S., Gonzalez, M., Gonzalez-Acuna, D., Chiang, G., Celis, J., Espejo, W., Mattatall, P. & Barra, R. (2015) Potential immunohaematological effects of persistent organic pollutants on chinstrap penguin. *Antarctic Science*, **27**, 373-381.

Jasarevic, E., Sieli, P.T., Twellman, E.E., Welsh, T.H., Jr., Schachtman, T.R., Roberts, R.M., Geary, D.C. & Rosenfeld, C.S. (2011) Disruption of adult expression of sexually selected traits by developmental exposure to bisphenol A. *Proceedings of the National Academy of Sciences of the United States of America*, **108**, 11715-11720.

Jaspers, V.L.B. (2015) Selecting the right bird model in experimental studies on endocrine disrupting chemicals. *Frontiers in Environmental Science*, **3**

Jaspers, V.L.B., Herzke, D., Eulaers, I., Gillespie, B.W. & Eens, M. (2013) Perfluoroalkyl substances in soft tissues and tail feathers of Belgian barn owls (*Tyto alba*) using statistical methods for left-censored data to handle non-detects. *Environment International*, **52**, 9-16.

Jenkins, J.A., Olivier, H.M., Draugelis-Dale, R.O., Eilts, B.E., Torres, L., Patino, R., Nilsen, E. & Goodbred, S.L. (2014) Assessing reproductive and endocrine parameters in male largescale suckers (*Catostomus macrocheilus*) along a contaminant gradient in the lower Columbia River, USA. *Science of the Total Environment*, **484**, 365-378.

Jensen, A.A. & Leffers, H. (2008) Emerging endocrine disrupters: perfluoroalkylated substances. *International Journal of Andrology*, **31**, 161-9.

Jensen, S. (1972) The PCB Story. *Ambio*, **1**, 123-131.

Jenssen, B.M. (2006) Endocrine-disrupting chemicals and climate change: A worst-case combination for arctic marine mammals and seabirds? *Environmental Health Perspectives*, **114**, 76-80.

Jenssen, B.M., Villanger, G.D., Gabrielsen, K.M., Bytingsvik, J., Bechshoft, T., Ciesielski, T.M., Sonne, C. & Dietz, R. (2015) Anthropogenic flank attack on polar bears: interacting consequences of climate warming and pollutant exposure. *Frontiers in Ecology and Evolution*, **3**

Jepson, P.D. & Law, R.J. (2016) Persistent pollutants, persistent threats. *Science*, **352**, 1388-9.

Jepson, P.D., Deaville, R., Barber, J.L., Aguilar, À., Borrell, A., Murphy, S., Barry, J., Brownlow, A., Barnett, J., Berrow, S., Cunningham, A.A., Davison, N.J., ten Doeschate, M., Esteban, R., Ferreira, M., Foote, A.D., Genov, T., Giménez, J., Loveridge, J., Llavona, Á., Martin, V., Maxwell, D.L., Papachlimitzou, A., Penrose, R., Perkins, M.W., Smith, B., de Stephanis, R., Tregenza, N., Verborgh, P., Fernandez, A. & Law, R.J. (2016) PCB pollution continues to impact populations of orcas and other dolphins in European waters. *Scientific Reports*, **6**, 18573.

Ji, K., Hong, S., Kho, Y. & Choi, K. (2013) Effects of Bisphenol S Exposure on Endocrine Functions and Reproduction of Zebrafish. *Environmental Science & Technology*, **47**, 8793-8800.

Jinhui, L., Yuan, C. & Wenjing, X. (2017) Polybrominated diphenyl ethers in articles: a review of its applications and legislation. *Environmental Science and Pollution Research*, **24**, 4312-4321.

Jobling, S. (2014) Endocrine disrupting chemical impacts on aquatic systems. *International Seminar on Nuclear War and Planetary Emergencies : 46th session : "E. Majorana" Centre for Scientific Culture, 19-24 August 2013, Erice, Italy* (ed. by A. Zichichi and R.C. Ragaini). World Scientific, New Jersery, USA.

Jobling, S. & Tyler, C.R. (2003) Endocrine disruption in wild freshwater fish. *Pure and Applied Chemistry*, **75**, 2219-2234.

Jobling, S. & Owen, R. (2013) Ethinyl oestradiol in the aquatic environment. In: *Late lessons from early warnings: science, precaution, innovation. EEA Report No 1/2013* eds. D. Gee, P. Grandjean, S.F. Hansen, S. Van den hove, M. Macgarvin, G.N. Jock Martin, D. Quist and D. Stanner). European Environmental Agency, Copenhagen.

Jobling, S., Nolan, M., Tyler, C.R., Brighty, G. & Sumpter, J.P. (1998) Widespread Sexual Disruption in Wild Fish. *Environmental Science & Technology*, **32**, 2498-2506.

Jobling, S., Beresford, N., Nolan, M., Rodgers-Gray, T., Brighty, G.C., Sumpter, J.P. & Tyler, C.R. (2002a) Altered Sexual Maturation and Gamete Production in Wild Roach (*Rutilus rutilus*) Living in Rivers That Receive Treated Sewage Effluents. *Biology of Reproduction*, **66**, 272-281.

Jobling, S., Williams, R., Johnson, A., Taylor, A., Gross-Sorokin, M., Nolan, M., Tyler, C.R., van Aerle, R., Santos, E. & Brighty, G. (2006) Predicted Exposures to Steroid Estrogens in U.K. Rivers Correlate with Widespread Sexual Disruption in Wild Fish Populations. *Environmental Health Perspectives*, **114**, 32-39.

Jobling, S., Coey, S., Whitmore, J.G., Kime, D.E., Van Look, K.J.W., McAllister, B.G., Beresford, N., Henshaw, A.C., Brighty, G., Tyler, C.R. & Sumpter, J.P. (2002b) Wild Intersex Roach (*Rutilus rutilus*) Have Reduced Fertility. *Biology of Reproduction*, **67**, 515-524.

Johnson, A.C. (2010) Natural variations in flow are critical in determining concentrations of point source contaminants in rivers: an estrogen example. *Environmental Science & Technology*, **44**, 7865-70.

Johnson, A.C. & Chen, Y. (2017) Does exposure to domestic wastewater effluent (including steroid estrogens) harm fish populations in the UK? *Science of the Total Environment*, **589**, 89-96.

Johnson, A.C., Williams, R.J. & Matthiessen, P. (2006) The potential steroid hormone contribution of farm animals to freshwaters, the United Kingdom as a case study. *Science of the Total Environment*, **362**, 166-78.

Johnson, A.C., Williams, R.J., Simpson, P. & Kanda, R. (2007) What difference might sewage treatment performance make to endocrine disruption in rivers? *Environmental Pollution*, **147**, 194-202.

Johnson, A.C., Dumont, E., Williams, R.J., Oldenkamp, R., Cisowska, I. & Sumpter, J.P. (2013) Do concentrations of ethinylestradiol, estradiol, and diclofenac in European rivers exceed proposed EU environmental quality standards? *Environmental Science & Technology*, **47**, 12297-304.

Johnson, A.C., Acreman, M.C., Dunbar, M.J., Feist, S.W., Giacomello, A.M., Gozlan, R.E., Hinsley, S.A., Ibbotson, A.T., Jarvie, H.P., Jones, J.I., Longshaw, M., Maberly, S.C., Marsh, T.J., Neal, C., Newman, J.R., Nunn, M.A., Pickup, R.W., Reynard, N.S., Sullivan, C.A., Sumpter, J.P. & Williams, R.J. (2009a) The British river of the future: How climate change and human activity might affect two contrasting river ecosystems in England. *Science of the Total Environment*, **407**, 4787-4798.

Johnson, K.E., Knopper, L.D., Schneider, D.C., Ollson, C.A. & Reimer, K.J. (2009b) Effects of local point source polychlorinated biphenyl (PCB) contamination on bone mineral density in deer mice (*Peromyscus maniculatus*). *Science of the Total Environment*, **407**, 5050-5055.

Jones, J. & Reynolds, J. (1997) Effects of pollution on reproductive behaviour of fishes. *Reviews in Fish Biology and Fisheries*, **7**, 463-491.

Jooste, A.M., Du Preez, L.H., Carr, J.A., Giesy, J.P., Gross, T.S., Kendall, R.J., Smith, E.E., Van Der Kraak, G.J. & Solomon, K.R. (2005a) Gonadal development of larval male *Xenopus laevis* exposed to atrazine in outdoor microcosms - Response. *Environmental Science & Technology*, **39**, 7759-7760.

Jooste, A.M., Du Preez, L.H., Carr, J.A., Giesy, J.P., Gross, T.S., Kendall, R.J., Smith, E.E., Van Der Kraak, G.L. & Solomon, K.R. (2005b) Gonadal Development of Larval Male *Xenopus laevis* Exposed to Atrazine in Outdoor Microcosms. *Environmental Science & Technology*, **39**, 5255-5261.

Judson, R., Richard, A., Dix, D.J., Houck, K., Martin, M., Kavlock, R., Dellarco, V., Henry, T., Holderman, T., Sayre, P., Tan, S., Carpenter, T. & Smith, E. (2009) The toxicity data landscape for environmental chemicals. *Environmental Health Perspectives*, **117**, 685-95.

Jürgens, M.D., Holthaus, K.I.E., Johnson, A.C., Smith, J.J.L., Hetheridge, M. & Williams, R.J. (2002) The potential for estradiol and ethinylestradiol degradation in english rivers. *Environmental Toxicology and Chemistry*, **21**, 480-488.

Kaiser, J. (2000) Endocrine disrupters. Panel cautiously confirms low-dose effects. *Science*, **290**, 695-7.

Kampa, E., Dworak, T., Laaser, C. & Vidaurre, R. (2010) European Regulations. *Green and Sustainable Pharmacy* (ed. by K. KüMmerer and M. Hempel). Springer-Verlag, Berlin.

Kantor, E.D., Rehm, C.D., Haas, J.S., Chan, A.T. & Giovannucci, E.L. (2015) Trends in Prescription Drug Use Among Adults in the United States From 1999-2012. *Jama*, **314**, 1818.

Kapraun, D.F., Wambaugh, J.F., Ring, C.L., Tornero-Velez, R. & Setzer, R.W. (2017) A Method for Identifying Prevalent Chemical Combinations in the U.S. Population. *Environmental Health Perspectives*, **125**, 087017.

Kasprzyk-Hordern, B., Dinsdale, R.M. & Guwy, A.J. (2008) The occurrence of pharmaceuticals, personal care products, endocrine disruptors and illicit drugs in surface water in South Wales, UK. *Water Research*, **42**, 3498-518.

Kavlock, R.J., Daston, G.P., DeRosa, C., Fenner-Crisp, P., Gray, L.E., Kaattari, S., Lucier, G., Luster, M., Mac, M.J., Maczka, C., Miller, R., Moore, J., Rolland, R., Scott, G., Sheehan, D.M., Sinks, T. & Tilson, H.A. (1996) Research needs for the risk assessment of health and environmental effects of endocrine disruptors: a report of the U.S. EPA-sponsored workshop. *Environ Health Perspect*, **104 Suppl 4**, 715-40.

Kean, E.F., Lyons, G. & Chadwick, E.A. (2013) *Persistent organic pollutants and indicators of otter health: other factors at play?* ChemTrust and the Cardiff University Otter Project.

Keil, F. (2010) Pharmaceuticals for Human Use: An Integrated Strategy for Reducing the Contamination of Water Bodies. *Green and Sustainable Pharmacy* (ed. by K. KüMmerer and M. Hempel). Springer-Verlag, Berlin.

Kelce, W.R., Stone, C.R., Laws, S.C., Gray, L.E., Kemppainen, J.A. & Wilson, E.M. (1995) Persistent DDT metabolite p,p'-DDE is a potent androgen receptor antagonist. *Nature*, **375**, 581-5.

Keller, V.D., Williams, R.J., Lofthouse, C. & Johnson, A.C. (2014) Worldwide estimation of river concentrations of any chemical originating from sewage-treatment plants using dilution factors. *Environmental Toxicology and Chemistry*, **33**, 447-52.

Keller, V.D., Lloyd, P., Terry, J.A. & Williams, R.J. (2015) Impact of climate change and population growth on a risk assessment for endocrine disruption in fish due to steroid estrogens in England and Wales. *Environmental Pollution*, **197**, 262-8.

Kelly, B.C., Ikonomou, M.G., Blair, J.D., Surridge, B., Hoover, D., Grace, R. & Gobas, F.A.P.C. (2009) Perfluoroalkyl Contaminants in an Arctic Marine Food Web: Trophic Magnification and Wildlife Exposure. *Environmental Science & Technology*, **43**, 4037-4043.

Khan, U. & Nicell, J.A. (2014) Contraceptive Options and Their Associated Estrogenic Environmental Loads: Relationships and Trade-Offs. *PLoS ONE*, **9**, e92630.

Kidd, K.A., Blanchfield, P.J., Mills, K.H., Palace, V.P., Evans, R.E., Lazorchak, J.M. & Flick, R.W. (2007) Collapse of a fish population after exposure to a synthetic estrogen. *Proceedings of the National Academy of Sciences*, **104**, 8897-8901.

Kidd, K.A., Paterson, M.J., Rennie, M.D., Podemski, C.L., Findlay, D.L., Blanchfield, P.J. & Liber, K. (2014) Direct and indirect responses of a freshwater food web to a potent synthetic oestrogen. *Philosophical Transactions of the Royal Society of London B: Biological Sciences*, **369**

Kinch, C.D., Ibhazehiebo, K., Jeong, J.-H., Habibi, H.R. & Kurrasch, D.M. (2015) Low-dose exposure to bisphenol A and replacement bisphenol S induces precocious hypothalamic neurogenesis in embryonic zebrafish. *Proceedings of the National Academy of Sciences of the United States of America*, **112**, 1475-1480.

Klaper, R.D. & Niemuth, N.J. (2016) On the unexpected reproductive impacts of metformin: A need for support and new directions for the evaluation of the impacts of pharmaceuticals in the environment. *Chemosphere*, **165**, 570-574.

Kloas, W., Lutz, I., Springer, T., Krueger, H., Wolf, J., Holden, L. & Hosmer, A. (2009) Does atrazine influence larval development and sexual differentiation in *Xenopus laevis*? *Toxicological Sciences*, **107**, 376-84.

Koppe, J.G. & Keys, J. (2002) PCBs and the precautionary principle. In: *Late lessons from early warnings: the precautionary principle 1896-2000* (ed. D. Gee). European Environment Agency

Kortenkamp, A. (2007) Ten years of mixing cocktails: a review of combination effects of endocrine-disrupting chemicals. *Environmental Health Perspectives*, **115 Suppl 1**, 98-105.

Kortenkamp, A. (2008) Low dose mixture effects of endocrine disrupters: implications for risk assessment and epidemiology. *International Journal of Andrology*, **31**, 233-40.

Kortenkamp, A., Bourguignon, J.P., Slama, R., Bergman, A., Demeneix, B., Ivell, R., Panzica, G., Trasande, L. & Zoeller, R.T. (2016) EU regulation of endocrine disruptors: a missed opportunity. *Lancet Diabetes & Endocrinology*, **4**, 649-650.

Koschorreck, J., Heiss, C., Wellmitz, J., Fliedner, A. & Rudel, H. (2015) The use of monitoring data in EU chemicals management--experiences and considerations from the German environmental specimen bank. *Environmental Science and Pollution Research*, **22**, 1597-611.

Kosma, C.I., Lambropoulou, D.A. & Albanis, T.A. (2015) Comprehensive study of the antidiabetic drug metformin and its transformation product guanylurea in Greek wastewaters. *Water research*, **70**, 436-448.

Kovacs, T.G., Gibbons, J.S., Tremblay, L.A., Oconnor, B.I., Martel, P.H. & Voss, R.H. (1995) The Effects of a Secondary-Treated Bleached Kraft Mill Effluent on Aquatic Organisms as Assessed by Short-Term and Long-Term Laboratory Tests. *Ecotoxicology and Environmental Safety*, **31**, 7-22.

Kramer, V.J., Etterson, M.A., Hecker, M., Murphy, C.A., Roesijadi, G., Spade, D.J., Spromberg, J.A., Wang, M. & Ankley, G.T. (2011) Adverse outcome pathways and ecological risk assessment: bridging to population-level effects. *Environmental Toxicology and Chemistry*, **30**, 64-76.

Kumar, V., Johnson, A.C., Trubiroha, A., Tumova, J., Ihara, M., Grabic, R., Kloas, W., Tanaka, H. & Kroupova, H.K. (2015) The challenge presented by progestins in ecotoxicological research: a critical review. *Environmental Science & Technology*, **49**, 2625-38.

Kümmerer, K. (2010) Rational Design of Molecules by Life Cycle Engineering. *Green and Sustainable Pharmacy* (ed. by K. KüMmerer and M. Hempel). Springer-Verlag, Berlin.

Kwok, K.Y., Yamazaki, E., Yamashita, N., Taniyasu, S., Murphy, M.B., Horii, Y., Petrick, G., Kallerborn, R., Kannan, K., Murano, K. & Lam, P.K.S. (2013) Transport of Perfluoroalkyl substances (PFAS) from an arctic glacier to downstream locations: Implications for sources. *Science of the Total Environment*, **447**, 46-55.

Lagadic, L., Katsiadaki, I., Biever, R., Guiney, P.D., Karouna-Renier, N., Schwarz, T. & Meador, J.P. (2017) Tributyltin: Advancing the Science on Assessing Endocrine Disruption with an Unconventional Endocrine-Disrupting Compound. *Reviews of Environmental Contamination and Toxicology*,

Lagarde, F., Beausoleil, C., Belcher, S.M., Belzunces, L.P., Emond, C., Guerbet, M. & Rousselle, C. (2015) Non-monotonic dose-response relationships and endocrine disruptors: a qualitative method of assessment. *Environmental Health*, **14**, 13.

Lahvis, G.P., Wells, R.S., Kuehl, D.W., Stewart, J.L., Rhinehart, H.L. & Via, C.S. (1995) Decreased lymphocyte responses in free-ranging bottlenose dolphins (*Tursiops truncatus*) are associated with increased concentrations of PCBs and DDT in peripheral blood. *Environmental Health Perspectives*, **103 Suppl 4**, 67-72.

Lamichhane, K. & Babcock, R. (2012) An economic appraisal of using source separation of human urine to contain and treat endocrine disrupters in the USA. *Journal of Environmental Monitoring*, **14**, 2557-2565.

Lamon, L., Dalla Valle, M., Critto, A. & Marcomini, A. (2009) Introducing an integrated climate change perspective in POPs modelling, monitoring and regulation. *Environmental Pollution*, **157**, 1971-80.

Lanctôt, C., Robertson, C., Navarro-Martin, L., Edge, C., Melvin, S.D., Houlahan, J. & Trudeau, V.L. (2013) Effects of the glyphosate-based herbicide Roundup WeatherMax (R) on metamorphosis of wood frogs (*Lithobates sylvaticus*) in natural wetlands. *Aquatic Toxicology*, **140**, 48-57.

Lanctôt, C., Navarro-Martín, L., Robertson, C., Park, B., Jackman, P., Pauli, B.D. & Trudeau, V.L. (2014) Effects of glyphosate-based herbicides on survival, development, growth and sex ratios of wood frog (*Lithobates sylvaticus*) tadpoles. II: Agriculturally relevant exposures to Roundup WeatherMax® and Vision® under laboratory conditions. *Aquatic Toxicology*, **154**, 291-303.

Land, M., de Wit, C.A., Cousins, I.T., Herzke, D., Johansson, J. & Martin, J.W. (2015) What is the effect of phasing out long-chain per- and polyfluoroalkyl substances on the concentrations of perfluoroalkyl acids and their precursors in the environment? A systematic review protocol. *Environmental Evidence*, **4**, 3.

Landry, K.A. & Boyer, T.H. (2016) Life cycle assessment and costing of urine source separation: Focus on nonsteroidal anti-inflammatory drug removal. *Water Research*, **105**, 487-495.

Langford, K.H. & Thomas, K.V. (2009) Determination of pharmaceutical compounds in hospital effluents and their contribution to wastewater treatment works. *Environment International*, **35**, 766-70.

Langlois, V.S., Carew, A.C., Pauli, B.D., Wade, M.G., Cooke, G.M. & Trudeau, V.L. (2010) Low levels of the herbicide atrazine alter sex ratios and reduce metamorphic success in *Rana pipiens* tadpoles raised in outdoor mesocosms. *Environmental Health Perspectives*, **118**, 552-7.

Langston, W.J., Pope, N.D., Davey, M., Langston, K.M., SC, O.H., Gibbs, P.E. & Pascoe, P.L. (2015) Recovery from TBT pollution in English Channel environments: A problem solved? *Marine Pollution Bulletin*, **95**, 551-64.

Larsson, D.G. & Forlin, L. (2002) Male-biased sex ratios of fish embryos near a pulp mill: temporary recovery after a short-term shutdown. *Environmental Health Perspectives*, **110**, 739-42.

Lau, C., Anitole, K., Hodes, C., Lai, D., Pfahles-Hutchens, A. & Seed, J. (2007) Perfluoroalkyl acids: a review of monitoring and toxicological findings. *Toxicological Sciences*, **99**, 366-94.

Law, R.J. & Jepson, P.D. (2017) Europe's insufficient pollutant remediation. *Science*, **356**, 148.

Law, R.J., Allchin, C.R. & Mead, L.K. (2005) Brominated diphenyl ethers in the blubber of twelve species of marine mammals stranded in the UK. *Marine Pollution Bulletin*, **50**, 356-359.

Law, R.J., Covaci, A., Harrad, S., Herzke, D., Abdallah, M.A.E., Femie, K., Toms, L.-M.L. & Takigami, H. (2014) Levels and trends of PBDEs and HBCDs in the global environment: Status at the end of 2012. *Environment International*, **65**, 147-158.

Le Boeuf, B.J., Giesy, J.P., Kannan, K., Kajiwara, N., Tanabe, S. & Debier, C. (2002) Organochlorine pollutants corrected in California sea lions revisited. *BMC ecology*, **2**, 11-11.

Le Fol, V., Ait-Aissa, S., Sonavane, M., Porcher, J.M., Balaguer, P., Cravedi, J.P., Zalko, D. & Brion, F. (2017) In vitro and in vivo estrogenic activity of BPA, BPF and BPS in zebrafish-specific assays. *Ecotoxicology and Environmental Safety*, **142**, 150-156.

Leat, E.H.K., Bourgeon, S., Eze, J.I., Muir, D.C.G., Williamson, M., Bustnes, J.O., Furness, R.W. & Borga, K. (2013) Perfluoroalkyl substances in eggs and plasma of an avian top predator, great skua (*Stercorarius skua*), in the north Atlantic. *Environmental Toxicology and Chemistry*, **32**, 569-576.

Leatherland, J.F. (1997) Endocrine and reproductive function in Great Lakes salmon. *Journal of Clean Technology* 381-385.

Lee, H.J. & Kim, G.B. (2015) An overview of polybrominated diphenyl ethers (PBDEs) in the marine environment. *Ocean Science Journal*, **50**, 119-142.

Leet, J.K., Sassman, S., Amberg, J.J., Olmstead, A.W., Lee, L.S., Ankley, G.T. & Sepulveda, M.S. (2015) Environmental hormones and their impacts on sex differentiation in fathead minnows. *Aquatic Toxicology*, **158**, 98-107.

Lemghich, I. & Benajiba, M.H. (2007) Survey of imposex in prosobranchs mollusks along the northern Mediterranean coast of Morocco. *Ecological Indicators*, **7**, 209-214.

Lenoir, A., Touchard, A., Devers, S., Christides, J.-P., Boulay, R. & Cuvillier-Hot, V. (2014) Ant cuticular response to phthalate pollution. *Environmental Science and Pollution Research*, **21**, 13446-13451.

Letcher, R.J., Su, G., Moore, J.N., Williams, L.L., Martin, P.A., de Solla, S.R. & Bowerman, W.W. (2015) Perfluorinated sulfonate and carboxylate compounds and precursors in herring gull eggs from across the Laurentian Great Lakes of North America: Temporal and recent spatial comparisons and exposure implications. *Science of the Total Environment*, **538**, 468-477.

Lewis, K.A., Green, A., Tzilivakis, J. & Warner, D. (2015) *The Pesticide Properties DataBase (PPDB) developed by the Agriculture & Environment Research Unit (AERU), University of Hertfordshire, 2006-2015*. <http://sitem.herts.ac.uk/aeru/ppdb/en/index.htm>.

Lewis, R.W., Billington, R., Debryune, E., Gamer, A., Lang, B. & Carpanini, F. (2002) Recognition of adverse and nonadverse effects in toxicity studies. *Toxicol Pathol*, **30**, 66-74.

Li, Y., Li, J. & Wang, L. (2013) Recycling of PBDEs Containing Plastics from Waste Electrical and Electronic Equipment (WEEE): A Review. *IEEE 10th International Conference on e-Business Engineering*, 407-412.

Li, Y., Zhu, G., Ng, W.J. & Tan, S.K. (2014) A review on removing pharmaceutical contaminants from wastewater by constructed wetlands: design, performance and mechanism. *Science of the Total Environment*, **468-469**, 908-32.

Li, Y., Lin, T., Hu, L., Feng, J. & Guo, Z. (2016) Time trends of polybrominated diphenyl ethers in East China Seas: Response to the booming of PBDE pollution industry in China. *Environment International*, **92-93**, 507-14.

Li, Y.-Y., Xu, W., Chen, X.-R., Lou, Q.-Q., Wei, W.-J. & Qin, Z.-F. (2015) Low concentrations of 17 beta-trenbolone induce female-to-male reversal and mortality in the frog Pelophylax nigromaculatus. *Aquatic Toxicology*, **158**, 230-237.

Lin, A.Y. & Reinhard, M. (2005) Photodegradation of common environmental pharmaceuticals and estrogens in river water. *Environmental Toxicology and Chemistry*, **24**, 1303-9.

Lind, P.M., Bergman, A., Olsson, M. & Örberg, J. (2003) Bone Mineral Density in Male Baltic Grey Seal (*Halichoerus grypus*). *Ambio*, **32**, 385-388.

Lindstrom, A.B., Strynar, M.J. & Libelo, E.L. (2011) Polyfluorinated compounds: past, present, and future. *Environmental Science & Technology*, **45**, 7954-61.

Liney, K.E., Jobling, S., Shears, J.A., Simpson, P. & Tyler, C.R. (2005) Assessing the sensitivity of different life stages for sexual disruption in roach (Rutilus rutilus) exposed to effluents from wastewater treatment works. *Environmental Health Perspectives*, **113**, 1299-307.

Little, A.G. & Seebacher, F. (2015) Temperature determines toxicity: bisphenol A reduces thermal tolerance in fish. *Environmental Pollution*, **197**, 84-9.

Lopezmartin, J.M., Ruizolmo, J. & Minano, S.P. (1994) Organochlorine Residue Levels in the European Mink (*Mustela lutreola*) in Northern Spain. *Ambio*, **23**, 294-295.

Louis, C., Dirtu, A.C., Stas, M., Guiot, Y., Malarvannan, G., Das, K., Costa, D.P., Crocker, D.E., Covaci, A. & Debier, C. (2014) Mobilisation of lipophilic pollutants from blubber in northern elephant seal pups (*Mirounga angustirostris*) during the post-weaning fast. *Environmental Research*, **132**, 438-448.

Lu, Q., Johnson, A.C., Juergens, M.D., Sweetman, A., Jin, L. & Whitehead, P. (2015) The distribution of Polychlorinated Biphenyls (PCBs) in the River Thames Catchment under the scenarios of climate change. *Science of the Total Environment*, **533**, 187-195.

Lucia, M., Verboven, N., Strom, H., Miljeteig, C., Gavrilo, M.V., Braune, B.M., Boertmann, D. & Gabrielsen, G.W. (2015) Circumpolar contamination in eggs of the high-arctic ivory gull *Pagophila eburnea*. *Environmental Toxicology and Chemistry*, **34**, 1552-1561.

Lundholm, C.E. (1997) DDE-induced eggshell thinning in birds: Effects of p,p’-DDE on the calcium and prostaglandin metabolism of the eggshell gland. *Comparative Biochemistry and Physiology Part C: Pharmacology, Toxicology and Endocrinology*, **118**, 113-128.

Lundholm, E. (1987) Thinning of eggshells in birds by DDE: Mode of action on the eggshell gland. *Comparative Biochemistry and Physiology Part C: Comparative Pharmacology*, **88**, 1-22.

Lyons, B.P., Bignell, J.P., Stentiford, G.D., Bolam, T.P., Rumney, H.S., Bersuder, P., Barber, J.L., Askem, C.E., Nicolaus, M.E. & Maes, T. (2017) Determining Good Environmental Status under the Marine Strategy Framework Directive: Case study for descriptor 8 (chemical contaminants). *Marine Environmental Research*, **124**, 118-129.

Lyons, G. (2003) Endocrine active substances and the need to improve environmental protection: An environmentalist's perspective. *Pure and Applied Chemistry*, **75**, 2593-2604.

Lyons, G. (2006) Viewpoint: Policy requirements for protecting wildlife from endocrine disruptors. *Environmental Health Perspectives*, **114**, 142-146.

Ma, J., Hung, H., Tian, C. & Kallenborn, R. (2011) Revolatilization of persistent organic pollutants in the Arctic induced by climate change. *Nature Climate Change*, **1**, 255-260.

Mace, G.M., Collar, N.J., Gaston, K.J., Hilton-Taylor, C., Akcakaya, H.R., Leader-Williams, N., Milner-Gulland, E.J. & Stuart, S.N. (2008) Quantification of extinction risk: IUCN's system for classifying threatened species. *Conservation Biology*, **22**, 1424-42.

Mackintosh, C.E., Maldonado, J., Jing, H.W., Hoover, N., Chong, A., Ikonomou, M.G. & Gobas, F. (2004) Distribution of phthalate esters in a marine aquatic food web: Comparison to polychlorinated biphenyls. *Environmental Science & Technology*, **38**, 2011-2020.

Mackintosh, S.A., Wallace, J.S., Gross, M.S., Navarro, D.D., Perez-Fuentetaja, A., Alaee, M., Montecastro, D. & Aga, D.S. (2015) Review on the occurrence and profiles of polybrominated diphenyl ethers in the Philippines. *Environ Int*, **85**, 314-26.

Magr, V.S. (2003) PCB treatment alternatives and research directions. *Journal of Environmental Engineering-Asce*, **129**, 961-965.

Magulova, K. & Priceputu, A. (2016) Global monitoring plan for persistent organic pollutants (POPs) under the Stockholm Convention: Triggering, streamlining and catalyzing global POPs monitoring. *Environmental Pollution*, **217**, 82-4.

Mandavilli, A. (2006) Health agency backs use of DDT against malaria. *Nature*, **443**, 250-1.

Manibusan, M.K. & Touart, L.W. (2017) A comprehensive review of regulatory test methods for endocrine adverse health effects. *Critical Reviews in Toxicology*, **47**, 433-481.

Månsson, N., Sörme, L., Wahlberg, C. & Bergbäck, B. (2008) Sources of Alkylphenols and Alkylphenol Ethoxylates in Wastewater—A Substance Flow Analysis in Stockholm, Sweden. *Water, Air, & Soil Pollution: Focus*, **8**, 445-456.

Margiotta-Casaluci, L., Owen, S.F., Cumming, R.I., de Polo, A., Winter, M.J., Panter, G.H., Rand-Weaver, M. & Sumpter, J.P. (2014) Quantitative cross-species extrapolation between humans and fish: the case of the anti-depressant fluoxetine. *PLoS One*, **9**, e110467.

Margot, J., Kienle, C., Magnet, A., Weil, M., Rossi, L., de Alencastro, L.F., Abegglen, C., Thonney, D., Chevre, N., Scharer, M. & Barry, D.A. (2013) Treatment of micropollutants in municipal wastewater: ozone or powdered activated carbon? *Science of the Total Environment*, **461-462**, 480-98.

Markandya, A., Taylor, T., Longo, A., Murty, M.N., Murty, S. & Dhavala, K. (2008) Counting the cost of vulture decline—An appraisal of the human health and other benefits of vultures in India. *Ecological Economics*, **67**, 194-204.

Markman, S., Mueller, C.T., Pascoe, D., Dawson, A. & Buchanan, K.L. (2011) Pollutants affect development in nestling starlings *Sturnus vulgaris*. *Journal of Applied Ecology*, **48**, 391-397.

Markman, S., Leitner, S., Catchpole, C., Barnsley, S., Müller, C.T., Pascoe, D. & Buchanan, K.L. (2008) Pollutants Increase Song Complexity and the Volume of the Brain Area HVC in a Songbird. *PLoS ONE*, **3**, e1674.

Mars, B., Heron, J., Kessler, D., Davies, N.M., Martin, R.M., Thomas, K.H. & Gunnell, D. (2017) Influences on antidepressant prescribing trends in the UK: 1995–2011. *Social Psychiatry and Psychiatric Epidemiology*, **52**, 193-200.

Marshall, D.J. & Rajkumar, A. (2003) Imposex in the indigenous *Nassarius kraussianus* (Mollusca: Neogastropoda) from South African harbours. *Marine Pollution Bulletin*, **46**, 1150-1155.

Martineau, D., Beland, P., Desjardins, C. & Lagace, A. (1987) Levels of Organochlorine Chemicals in Tissues of Beluga Whales (Delphinapterus-Leucas) from the St-Lawrence Estuary, Quebec, Canada. *Archives of Environmental Contamination and Toxicology*, **16**, 137-147.

Marty, M.S., Blankinship, A., Chambers, J., Constantine, L., Kloas, W., Kumar, A., Lagadic, L., Meador, J., Pickford, D., Schwarz, T. & Verslycke, T. (2017) Population-Relevant Endpoints in the Evaluation of Endocrine-Active Substances (EAS) for Ecotoxicological Hazard and Risk Assessment. *Integrated Environmental Assessment and Management*, **13**, 317-330.

Marx-Stoelting, P., Niemann, L., Ritz, V., Ulbrich, B., Gall, A., Hirsch-Ernst, K.I., Pfeil, R. & Solecki, R. (2014) Assessment of three approaches for regulatory decision making on pesticides with endocrine disrupting properties. *Regulatory Toxicology and Pharmacology*, **70**, 590-604.

Massart, S., Redivo, B., Flarnion, E., Mandiki, S.N.M., Falisse, E., Milla, S. & Kestemont, P. (2015) The trenbolone acetate affects the immune system in rainbow trout, *Oncorhynchus mykiss*. *Aquatic Toxicology*, **163**, 109-120.

Mathieu-Denoncourt, J., de Solla, S.R. & Langlois, V.S. (2015) Chronic exposures to monomethyl phthalate in Western clawed frogs. *General and Comparative Endocrinology*, **219**, 53-63.

Matta, M.B., Linse, J., Cairncross, C., Francendese, L. & Kocan, R.M. (2001) Reproductive and transgenerational effects of methylmercury or Aroclor 1268 on *Fundulus heteroclitus*. *Environmental Toxicology and Chemistry*, **20**, 327-335.

Matthiessen, P. (2013) Detection, monitoring, and control of tributyltin--an almost complete success story. *Environmental Toxicology and Chemistry*, **32**, 487-9.

Matthiessen, P. & Gibbs, P.E. (1998) Critical appraisal of the evidence for tributyltin-mediated endocrine disruption in mollusks. *Environmental Toxicology and Chemistry*, **17**, 37-43.

Matthiessen, P. & Weltje, L. (2015) A review of the effects of azole compounds in fish and their possible involvement in masculinization of wild fish populations. *Critical Reviews in Toxicology*, **45**, 453-67.

Matthiessen, P., Wheeler, J.R. & Weltje, L. (2018) A review of the evidence for endocrine disrupting effects of current-use chemicals on wildlife populations. *Critical Reviews in Toxicology*, **48**, 195-216.

Matthiessen, P., Arnold, D., Johnson, A.C., Pepper, T.J., Pottinger, T.G. & Pulman, K.G. (2006) Contamination of headwater streams in the United Kingdom by oestrogenic hormones from livestock farms. *Sci Total Environ*, **367**, 616-30.

Matthiessen, P., Ankley, G.T., Biever, R.C., Bjerregaard, P., Borgert, C., Brugger, K., Blankinship, A., Chambers, J., Coady, K.K., Constantine, L., Dang, Z., Denslow, N.D., Dreier, D.A., Dungey, S., Gray, L.E., Gross, M., Guiney, P.D., Hecker, M., Holbech, H., Iguchi, T., Kadlec, S., Karouna-Renier, N.K., Katsiadaki, I., Kawashima, Y., Kloas, W., Krueger, H., Kumar, A., Lagadic, L., Leopold, A., Levine, S.L., Maack, G., Marty, S., Meador, J., Mihaich, E., Odum, J., Ortego, L., Parrott, J., Pickford, D., Roberts, M., Schaefers, C., Schwarz, T., Solomon, K., Verslycke, T., Weltje, L., Wheeler, J.R., Williams, M., Wolf, J.C. & Yamazaki, K. (2017) Recommended approaches to the scientific evaluation of ecotoxicological hazards and risks of endocrine-active substances. *Integrated Environmental Assessment and Management*, **13**, 267-279.

McGettigan, P. & Henry, D. (2013) Use of non-steroidal anti-inflammatory drugs that elevate cardiovascular risk: an examination of sales and essential medicines lists in low-, middle-, and high-income countries. *PLoS Medicine*, **10**, e1001388.

McKinney, M.A., Peacock, E. & Letcher, R.J. (2009) Sea Ice-associated Diet Change Increases the Levels of Chlorinated and Brominated Contaminants in Polar Bears. *Environmental Science & Technology*, **43**, 4334-4339.

McKinney, M.A., De Guise, S., Martineau, D., Beland, P., Lebeuf, M. & Letcher, R.J. (2006) Organohalogen contaminants and metabolites in beluga whale (*Delphinapterus leucas*) liver from two Canadian populations. *Environmental Toxicology and Chemistry*, **25**, 1246-1257.

McKinney, M.A., Dietz, R., Sonne, C., De Guise, S., Skirnisson, K., Karlsson, K., Steingrimsson, E. & Letcher, R.J. (2011a) Comparative hepatic microsomal biotransformation of selected PBDEs, including decabromodiphenyl ether, and decabromodiphenyl ethane flame retardants in Arctic marine-feeding mammals. *Environ Toxicol Chem*, **30**, 1506-14.

McKinney, M.A., Letcher, R.J., Aars, J., Born, E.W., Branigan, M., Dietz, R., Evans, T.J., Gabrielsen, G.W., Peacock, E. & Sonne, C. (2011b) Flame retardants and legacy contaminants in polar bears from Alaska, Canada, East Greenland and Svalbard, 2005-2008. *Environment International*, **37**, 365-374.

McKinney, M.A., Iverson, S.J., Fisk, A.T., Sonne, C., Riget, F.F., Letcher, R.J., Arts, M.T., Born, E.W., Rosing-Asvid, A. & Dietz, R. (2013) Global change effects on the long-term feeding ecology and contaminant exposures of East Greenland polar bears. *Global Change Biology*, **19**, 2360-72.

McLean, A.R., Adlen, E.K., Cardis, E., Elliott, A., Goodhead, D.T., Harms-Ringdahl, M., Hendry, J.H., Hoskin, P., Jeggo, P.A., Mackay, D.J.C., Muirhead, C.R., Shepherd, J., Shore, R.E., Thomas, G.A., Wakeford, R. & Godfray, H.C.J. (2017) A restatement of the natural science evidence base concerning the health effects of low-level ionizing radiation. *Proceedings of the Royal Society B — Biological Sciences*,

McMaster, M.E., Van Der Kraak, G.J. & Munkittrick, K.R. (1996) An Epidemiological Evaluation of the Biochemical Basis for Steroid Hormonal Depressions in Fish Exposed to Industrial Wastes. *Journal of Great Lakes Research*, **22**, 153-171.

McNabb, A.F.M., Larsen, C.T. & Pooler, P.S. (2004) Ammonium Perchlorate Effects on Thyroid Function and Growth in Bobwhite Quail Chicks. *Environmental Toxicology and Chemistry*, **23**, 997.

Mennigen, J.A., Stroud, P., Zamora, J.M., Moon, T.W. & Trudeau, V.L. (2011) Pharmaceuticals as Neuroendocrine Disruptors: Lessons Learned from Fish on Prozac. *Journal of Toxicology and Environmental Health — Part B*, **14**, 387-412.

Messerlian, C., Martinez, R.M., Hauser, R. & Baccarelli, A.A. (2017) 'Omics' and endocrine-disrupting chemicals - new paths forward. *Nature Reviews Endocrinology*,

Miege, C., Choubert, J.M., Ribeiro, L., Eusebe, M. & Coquery, M. (2009) Fate of pharmaceuticals and personal care products in wastewater treatment plants -- conception of a database and first results. *Environmental Pollution*, **157**, 1721-6.

Milla, S., Depiereux, S. & Kestemont, P. (2011) The effects of estrogenic and androgenic endocrine disruptors on the immune system of fish: a review. *Ecotoxicology*, **20**, 305-319.

Miller, V.M., Sanchez-Morrissey, S., Brosch, K.O. & Seegal, R.F. (2012) Developmental coexposure to polychlorinated biphenyls and polybrominated diphenyl ethers has additive effects on circulating thyroxine levels in rats. *Toxicological Sciences*, **127**, 76-83.

Mills, L.J. & Chichester, C. (2005) Review of evidence: Are endocrine-disrupting chemicals in the aquatic environment impacting fish populations? *Science of the Total Environment*, **343**, 1-34.

Milnes, M.R. & Guillette, L.J., Jr. (2008) Alligator Tales: New Lessons about Environmental Contaminants from a Sentinel Species. *Bioscience*, **58**, 1027-1036.

Milnes, M.R., Bermudez, D.S., Bryan, T.A., Gunderson, M.P. & Guillette, L.J. (2005) Altered neonatal development and endocrine function in *Alligator mississippiensis* associated with a contaminated environment. *Biology of Reproduction*, **73**, 1004-1010.

Milnes, M.R., Bryan, T.A., Katsu, Y., Kohno, S., Moore, B.C., Iguchi, T. & Guillette, L.J. (2008) Increased Posthatching Mortality and Loss of Sexually Dimorphic Gene Expression in Alligators (*Alligator mississippiensis*) from a Contaminated Environment. *Biology of Reproduction*, **78**, 932-938.

Mo, L., Wu, J.-P., Luo, X.-J., Zou, F.-S. & Mai, B.-X. (2012) Bioaccumulation of polybrominated diphenyl ethers, decabromodiphenyl ethane, and 1,2-bis(2,4,6-tribromophenoxy) ethane flame retardants in kingfishers (*Alcedo atthis*) from an electronic waste-recycling site in South China. *Environmental Toxicology and Chemistry*, **31**, 2153-2158.

Molnar, P.K., Lewis, M.A. & Derocher, A.E. (2014) Estimating Allee dynamics before they can be observed: polar bears as a case study. *PLoS One*, **9**, e85410.

Mommaerts, V., Hagenaars, A., Meyer, J., De Coen, W., Swevers, L., Mosallanejad, H. & Smagghe, G. (2011) Impact of a perfluorinated organic compound PFOS on the terrestrial pollinator *Bombus terrestris* (Insecta, Hymenoptera). *Ecotoxicology*, **20**, 447-456.

Moreau, M.F., Surico-Bennett, J., Vicario-Fisher, M., Gerads, R., Gersberg, R.M. & Hurlbert, S.H. (2007) Selenium, arsenic, DDT and other contaminants in four fish species in the Salton Sea, California, their temporal trends, and their potential impact on human consumers and wildlife. *Lake and Reservoir Management*, **23**, 536-569.

Moreman, J., Lee, O., Trznadel, M., David, A., Kudoh, T. & Tyler, C.R. (2017) Acute Toxicity, Teratogenic, and Estrogenic Effects of Bisphenol A and Its Alternative Replacements Bisphenol S, Bisphenol F, and Bisphenol AF in Zebrafish Embryo-Larvae. *Environmental Science & Technology*, **51**, 12796-12805.

Moreman, J., Takesono, A., Trznadel, M., Winter, M.J., Perry, A., Wood, M.E., Rogers, N.J., Kudoh, T. & Tyler, C.R. (2018) Estrogenic Mechanisms and Cardiac Responses Following Early Life Exposure to Bisphenol A (BPA) and Its Metabolite 4-Methyl-2,4-bis( p-hydroxyphenyl)pent-1-ene (MBP) in Zebrafish. *Environmental Science & Technology*, **52**, 6656-6665.

Morillo, E. & Villaverde, J. (2017) Advanced technologies for the remediation of pesticide-contaminated soils. *Science of the Total Environment*, **586**, 576-597.

Morrissey, C.A., Stanton, D.W.G., Tyler, C.R., Pereira, M.G., Newton, J., Durance, I. & Ormerod, S.J. (2014) Developmental impairment in eurasian dipper nestlings exposed to urban stream pollutants. *Environmental Toxicology and Chemistry*, **33**, 1315-1323.

Morton, B. (2009) Recovery from imposex by a population of the dogwhelk, *Nucella lapillus* (Gastropoda: Caenogastropoda), on the southeastern coast of England since May 2004: a 52-month study. *Marine Pollution Bulletin*, **58**, 1530-8.

Morvannou, A., Forquet, N., Michel, S., Troesch, S. & Molle, P. (2015) Treatment performances of French constructed wetlands: results from a database collected over the last 30 years. *Water Science and Technology*, **71**, 1333-9.

Mukhi, S. & Patino, R. (2007) Effects of prolonged exposure to perchlorate on thyroid and reproductive function in zebrafish. *Toxicological Sciences*, **96**, 246-54.

Murata, S., Takahashi, S., Agusa, T., Thomas, N.J., Kannan, K. & Tanabe, S. (2008) Contamination status and accumulation profiles of organotins in sea otters (*Enhydra lutris*) found dead along the coasts of California, Washington, Alaska (USA), and Kamchatka (Russia). *Marine Pollution Bulletin*, **56**, 641-649.

Mutha, N.H., Patel, M. & Premnath, V. (2006) Plastics materials flow analysis for India. *Resources, Conservation and Recycling*, **47**, 222-244.

Nadal, M., Marques, M., Mari, M. & Domingo, J.L. (2015) Climate change and environmental concentrations of POPs: A review. *Environmental Research*, **143**, 177-85.

Naderi, M., Wong, M.Y.L. & Gholami, F. (2014) Developmental exposure of zebrafish (Danio rerio) to bisphenol-S impairs subsequent reproduction potential and hormonal balance in adults. *Aquatic Toxicology*, **148**, 195-203.

Naidoo, V. & Swan, G.E. (2009) Diclofenac toxicity in Gyps vulture is associated with decreased uric acid excretion and not renal portal vasoconstriction. *Comparative Biochemistry and Physiology, Part C*, **149**, 269-74.

Naidoo, V., Wolter, K., Cromarty, D., Diekmann, M., Duncan, N., Meharg, A.A., Taggart, M.A., Venter, L. & Cuthbert, R. (2010) Toxicity of non-steroidal anti-inflammatory drugs to *Gyps* vultures: a new threat from ketoprofen. *Biology Letters*, **6**, 339-41.

Nash, J.P., Kime, D.E., Van der Ven, L.T.M., Wester, P.W., Brion, F., Maack, G., Stahlschmidt-Allner, P. & Tyler, C.R. (2004) Long-term exposure to environmental concentrations of the pharmaceutical ethynylestradiol causes reproductive failure in fish. *Environmental Health Perspectives*, **112**, 1725-1733.

Needhidasan, S., Samuel, M. & Chidambaram, R. (2014) Electronic waste - an emerging threat to the environment of urban India. *Journal of Environmental Health Science and Engineering*, **12**, 36.

Negrão de Carvalho, R., Ceriani, L., Ippolito, A. & Lettieri, T. (2015) Development of the First Watch List under the Environmental Quality Standards Directive. *EUR - Scientific and Technical Research Reports*,

Neigh, A.M., Zwiernik, M.J., Bradley, P.W., Kay, D.P., Park, C.S., Jones, P.D., Newsted, J.L., Blankenship, A.L. & Giesy, J.P. (2006) Tree swallow (*Tachycineta bicolor*) exposure to polychlorinated biphenyls at the Kalamazoo River Superfund site, Michigan, USA. *Environmental Toxicology and Chemistry*, **25**, 428-437.

Nelson, W.G. & Bergen, B.J. (2012) The New Bedford Harbor Superfund site long-term monitoring program (1993-2009). *Environmental Monitoring and Assessment*, **184**, 7531-50.

Neuman-Lee, L.A., Carr, J., Vaughn, K. & French, S.S. (2015) Physiological effects of polybrominated diphenyl ether (PBDE-47) on pregnant gartersnakes and resulting offspring. *General and Comparative Endocrinology*, **219**, 143-151.

Newton, I. (1973) Egg Breakage and Breeding Failure in British Merlins. *Bird Study*, **20**, 241-244.

Newton, I. (2013) Organochloride pesticides and birds. *British Birds*, **106**, 189-205.

Newton, I. & Haas, M.B. (1988) Pollutants in merlin eggs and their effects on breeding. *British Birds*, **81**, 258-269.

NHS choices (2017) *Type 2 diabetes - Treatment*. <http://www.nhs.uk/Conditions/Diabetes-type2/Pages/Treatment.aspx>.

NHS England (2017) *Personalised Medicine*. [https://www.england.nhs.uk/healthcare-science/personalisedmedicine/#](https://www.england.nhs.uk/healthcare-science/personalisedmedicine/).

Nicolopoulou-Stamati, P., Hens, L. & Sasco, A.J. (2015) Cosmetics as endocrine disruptors: are they a health risk? *Reviews in Endocrine and Metabolic Disorders*, **16**, 373-83.

Niemuth, N.J. & Klaper, R.D. (2015) Emerging wastewater contaminant metformin causes intersex and reduced fecundity in fish. *Chemosphere*, **135**, 38-45.

Nizzetto, L., Macleod, M., Borga, K., Cabrerizo, A., Dachs, J., Di Guardo, A., Ghirardello, D., Hansen, K.M., Jarvis, A., Lindroth, A., Ludwig, B., Monteith, D., Perlinger, J.A., Scheringer, M., Schwendenmann, L., Semple, K.T., Wick, L.Y., Zhang, G. & Jones, K.C. (2010) Past, present, and future controls on levels of persistent organic pollutants in the global environment. *Environmental Science & Technology*, **44**, 6526-31.

Noel, M., Barrett-Lennard, L., Guinet, C., Dangerfield, N. & Ross, P.S. (2009) Persistent organic pollutants (POPs) in killer whales (*Orcinus orca*) from the Crozet Archipelago, southern Indian Ocean. *Marine Environmental Research*, **68**, 196-202.

Nordstad, T., Moe, B., Bustnes, J.O., Bech, C., Chastel, O., Goutte, A., Sagerup, K., Trouve, C., Herzke, D. & Gabrielsen, G.W. (2012) Relationships between POPs and baseline corticosterone levels in black-legged kittiwakes (*Rissa tridactyla*) across their breeding cycle. *Environmental Pollution*, **164**, 219-226.

Norman Network (2012-2018) *NORMAN: Network of reference laboratories, research centres and related organisations for monitoring of emerging environmental substances*. <http://www.norman-network.net/>.

Noyes, P.D., McElwee, M.K., Miller, H.D., Clark, B.W., Van Tiem, L.A., Walcott, K.C., Erwin, K.N. & Levin, E.D. (2009) The toxicology of climate change: environmental contaminants in a warming world. *Environment International*, **35**, 971-86.

Nunn, A.D., Harvey, J.P. & Cowx, I.G. (2007) Variations in the spawning periodicity of eight fish species in three English lowland rivers over a 6 year period, inferred from 0+ year fish length distributions. *Journal of Fish Biology*, **70**, 1254-1267.

Oberemok, V.V., Laikova, K.V., Gninenko, Y.I., Zaitsev, A.S., Nyadar, P.M. & Adeyemi, T.A. (2015) A short history of insecticides. *Journal of Plant Protection Research*, **55**

OECD/OCDE (2009) *The 2007 OECD list of high production volume chemicals*. <http://www.oecd.org/officialdocuments/publicdisplaydocumentpdf/?cote=ENV/JM/MONO(2009)40&doclanguage=en>.

OECD/OCDE (2011) Test No. 234: Fish Sexual Development Test.

OECD/OCDE (2017) *OECD Work Related to Endocrine Disrupters*. <http://www.oecd.org/env/ehs/testing/oecdworkrelatedtoendocrinedisrupters.htm>.

OECD/OCDE (2018a) *Avian Toxicity Testing*. <http://www.oecd.org/chemicalsafety/testing/avian-toxicity-testing.htm>.

OECD/OCDE (2018b) *Adverse Outcome Pathways, Molecular Screening and Toxicogenomics*. <http://www.oecd.org/chemicalsafety/testing/adverse-outcome-pathways-molecular-screening-and-toxicogenomics.htm>.

Oehlmann, J., Fioroni, P., Stroben, E. & Markert, B. (1996) Tributyltin (TBT) effects on *Ocinebrina aciculata* (Gastropoda: Muricidae): Imposex development, sterilization, sex change and population decline. *Science of the Total Environment*, **188**, 205-223.

Oehlmann, J., Schulte-Oehlmann, U., Kloas, W., Jagnytsch, O., Lutz, I., Kusk, K.O., Wollenberger, L., Santos, E.M., Paull, G.C., Van Look, K.J.W. & Tyler, C.R. (2009) A critical analysis of the biological impacts of plasticizers on wildlife. *Philosophical Transactions of the Royal Society B-Biological Sciences*, **364**, 2047-2062.

Oka, T., Tooi, O., Mitsui, N., Miyahara, M., Ohnishi, Y., Takase, M., Kashiwagi, A., Shinkai, T., Santo, N. & Iguchi, T. (2008) Effect of atrazine on metamorphosis and sexual differentiation in *Xenopus laevis*. *Aquatic Toxicology*, **87**, 215-226.

Oller, I., Malato, S. & Sanchez-Perez, J.A. (2011) Combination of Advanced Oxidation Processes and biological treatments for wastewater decontamination -- a review. *Science of the Total Environment*, **409**, 4141-66.

Olsen, P., Fuller, P. & Marples, T.G. (1993) Pesticide-related Eggshell Thinning in Australian Raptors. *Emu*, **93**, 1-11.

Olsen, P., Emison, B., Mooney, N. & Brothers, N. (1992) DDT and dieldrin: effects on resident Peregrine Falcon populations in south-eastern Australia. *Ecotoxicology*, **1**, 89-100.

Oosterhuis, M., Sacher, F. & ter Laak, T.L. (2013) Prediction of concentration levels of metformin and other high consumption pharmaceuticals in wastewater and regional surface water based on sales data. *Science of the Total Environment*, **442**, 380-388.

Orlando, E.F. & Ellestad, L.E. (2014) Sources, concentrations, and exposure effects of environmental gestagens on fish and other aquatic wildlife, with an emphasis on reproduction. *General and Comparative Endocrinology*, **203**, 241-9.

Ormerod, S.J., Tyler, S.J. & Jüttner, I. (2000) Effects of point-source PCB contamination on breeding performance and post-fledging survival in the dipper *Cinclus cinclus*. *Environmental Pollution*, **110**, 505-513.

Overturf, M.D., Anderson, J.C., Pandelides, Z., Beyger, L. & Holdway, D.A. (2015) Pharmaceuticals and personal care products: A critical review of the impacts on fish reproduction. *Critical Reviews in Toxicology*, **45**, 469-491.

Owen, R. & Jobling, S. (2012) Environmental science: The hidden costs of flexible fertility. *Nature*, **485**, 441.

Page, S.W. & Gautier, P. (2012) Use of antimicrobial agents in livestock. *Revue scientifique et technique (International Office of Epizootics)* **31**, 145-188.

Pain, D.J., Burneleau, G., Bavoux, C. & Wyatt, C. (1999) Levels of polychlorinated biphenlyls, organochlorine pesticides, mercury and lead in relation to shell thickness in marsh harrier (*Circus aeruginosus*) eggs from Charente-Maritime, France. *Environmental Pollution*, **104**, 61-68.

Pain, D.J., Bowden, C.G.R., Cunningham, A.A., Cuthbert, R., Das, D., Gilbert, M., Jakati, R.D., Jhala, Y., Khan, A.A., Naidoo, V., Lindsay Oaks, J., Parry-Jones, J., Prakash, V., Rahmani, A., Ranade, S.P., Sagar Baral, H., Ram Senacha, K., Saravanan, S., Shah, N., Swan, G., Swarup, D., Taggart, M.A., Watson, R.T., Virani, M.Z., Wolter, K. & Green, R.E. (2008) The race to prevent the extinction of South Asian vultures. *Bird Conservation International*, **18**

Palace, V.P., Wautier, K.G., Evans, R.E., Blanchfield, P.J., Mills, K.H., Chalanchuk, S.M., Godard, D., McMaster, M.E., Tetreault, G.R., Peters, L.E., Vandenbyllaardt, L. & Kidd, K.A. (2006) Biochemical and histopathological effects in pearl dace (*Margariscus margarita*) chronically exposed to a synthetic estrogen in a whole lake experiment. *Environmental Toxicology and Chemistry*, **25**, 1114-1125.

Papoulias, D.M., Tillitt, D.E., Talykina, M.G., Whyte, J.J. & Richter, C.A. (2014) Atrazine reduces reproduction in Japanese medaka (*Oryzias latipes*). *Aquatic Toxicology*, **154**, 230-9.

Park, J.W., Rinchard, J., Liu, F., Anderson, T.A., Kendall, R.J. & Theodorakis, C.W. (2006) The thyroid endocrine disruptor perchlorate affects reproduction, growth, and survival of mosquitofish. *Ecotoxicology and Environmental Safety*, **63**, 343-52.

Parrott, J.L., Bjerregaard, P., Brugger, K.E., Gray, L.E., Iguchi, T., Kadlec, S.M., Weltje, L. & Wheeler, J.R. (2017) Uncertainties in Biological Responses that Influence Hazard and Risk Approaches to the Regulation of Endocrine Active Substances. *Integrated Environmental Assessment and Management*, **13**, 293-301.

Pascoal, S., Carvalho, G., Vasieva, O., Hughes, R., Cossins, A., Fang, Y., Ashelford, K., Olohan, L., Barroso, C., Mendo, S. & Creer, S. (2013) Transcriptomics and in vivo tests reveal novel mechanisms underlying endocrine disruption in an ecological sentinel, *Nucella lapillus*. *Molecular Ecology*, **22**, 1589-608.

Passatore, L., Rossetti, S., Juwarkar, A.A. & Massacci, A. (2014) Phytoremediation and bioremediation of polychlorinated biphenyls (PCBs): state of knowledge and research perspectives. *Journal of Hazardous Materials*, **278**, 189-202.

Patisaul, H.B., Roberts, S.C., Mabrey, N., McCaffrey, K.A., Gear, R.B., Braun, J., Belcher, S.M. & Stapleton, H.M. (2013) Accumulation and endocrine disrupting effects of the flame retardant mixture Firemaster(R) 550 in rats: an exploratory assessment. *Journal of Biochemical and Molecular Toxicology*, **27**, 124-36.

Patra, R.W., Chapman, J.C., Lim, R.P. & Gehrke, P.C. (2007) The effects of three organic chemicals on the upper thermal tolerances of four freshwater fishes. *Environmental Toxicology and Chemistry*, **26**, 1454-9.

Paul, A.G., Hammen, V.C., Hickler, T., Karlson, U.G., Jones, K.C. & Sweetman, A.J. (2012) Potential implications of future climate and land-cover changes for the fate and distribution of persistent organic pollutants in Europe. *Global Ecology and Biogeography*, **21**, 64-74.

Paulos, P., Runnalls, T.J., Nallani, G., La Point, T., Scott, A.P., Sumpter, J.P. & Huggett, D.B. (2010) Reproductive responses in fathead minnow and Japanese medaka following exposure to a synthetic progestin, Norethindrone. *Aquatic Toxicology*, **99**, 256-62.

Perkins, D.N., Brune Drisse, M.N., Nxele, T. & Sly, P.D. (2014) E-waste: a global hazard. *Annals of Global Health*, **80**, 286-95.

Persson, S., Rotander, A., Karrman, A., van Bavel, B. & Magnusson, U. (2013) Perfluoroalkyl acids in subarctic wild male mink (*Neovison vison*) in relation to age, season and geographical area. *Environment International*, **59**, 425-30.

Pickford, K.A., Thomas-Jones, R.E., Wheals, B., Tyler, C.R. & Sumpter, J.P. (2003) Route of exposure affects the oestrogenic response of fish to 4-tert-nonylphenol. *Aquatic Toxicology*, **65**, 267-279.

Pittman, H.T., Bowerman, W.W., Grim, L.H., Grubb, T.G., Bridges, W.C. & Wierda, M.R. (2015) Using nestling plasma to assess long-term spatial and temporal concentrations of organochlorine compounds in bald eagles within Voyageurs National Park, Minnesota, USA. *Chemosphere*, **123**, 79-86.

Plastics Europe (2016) *Bisphenol A - European Information Centre on Bisphenol A*. <http://www.bisphenol-a-europe.org>.

Polakof, S., Moon, T.W., Aguirre, P., Skiba-Cassy, S. & Panserat, S. (2011) Glucose homeostasis in rainbow trout fed a high-carbohydrate diet: metformin and insulin interact in a tissue-dependent manner. *American Journal of Physiology-Regulatory Integrative and Comparative Physiology*, **300**, R166-R174.

Post, G.B., Cohn, P.D. & Cooper, K.R. (2012) Perfluorooctanoic acid (PFOA), an emerging drinking water contaminant: a critical review of recent literature. *Environmental Research*, **116**, 93-117.

Pothitou, P. & Voutsa, D. (2008) Endocrine disrupting compounds in municipal and industrial wastewater treatment plants in Northern Greece. *Chemosphere*, **73**, 1716-23.

Powley, C.R., George, S.W., Russell, M.H., Hoke, R.A. & Buck, R.C. (2008) Polyfluorinated chemicals in a spatially and temporally integrated food web in the Western Arctic. *Chemosphere*, **70**, 664-672.

Prevedouros, K., MacLeod, M., Jones, K.C. & Sweetman, A.J. (2004) Modelling the fate of persistent organic pollutants in Europe: parameterisation of a gridded distribution model. *Environmental Pollution*, **128**, 251-261.

Prevedouros, K., Cousins, I.T., Buck, R.C. & Korzeniowski, S.H. (2006) Sources, fate and transport of perfluorocarboxylates. *Environmental Science & Technology*, **40**, 32-44.

Price, S. & Tait, A. (2012) Disposal of medicinal products. *Veterinary Record*, **170**, 28.

Purdom, C.E., Hardiman, P.A., Bye, V.V.J., Eno, N.C., Tyler, C.R. & Sumpter, J.P. (1994) Estrogenic Effects of Effluents from Sewage Treatment Works. *Chemistry and Ecology*, **8**, 275-285.

Qi, Z., Chen, T., Bai, S., Yan, M., Lu, S., Buekens, A., Yan, J., Bulmau, C. & Li, X. (2014) Effect of temperature and particle size on the thermal desorption of PCBs from contaminated soil. *Environmental Science and Pollution Research*, **21**, 4697-704.

Qiu, W., Zhao, Y., Yang, M., Farajzadeh, M., Pan, C. & Wayne, N.L. (2016) Actions of Bisphenol A and Bisphenol S on the Reproductive Neuroendocrine System During Early Development in Zebrafish. *Endocrinology*, **157**, 636-47.

Quinete, N., Lavandier, R., Dias, P., Taniguchi, S., Montone, R. & Moreira, I. (2011) Specific profiles of polybrominated diphenylethers (PBDEs) and polychlorinated biphenyls (PCBs) in fish and tucuxi dolphins from the estuary of Paraiba do Sul River, Southeastern Brazil. *Marine Pollution Bulletin*, **62**, 440-446.

Raach, M., Lebeuf, M. & Pelletier, E. (2011) PBDEs and PCBs in the liver of the St Lawrence Estuary beluga (*Delphinapterus leucas*): a comparison of levels and temporal trends with the blubber. *Journal of Environmental Monitoring*, **13**, 649-656.

Rahman, F., Langford, K.H., Scrimshaw, M.D. & Lester, J.N. (2001) Polybrominated diphenyl ether (PBDE) flame retardants. *Science of The Total Environment*, **275**, 1-17.

Rainwater, T.R., Wood, M.B., Millam, J.R. & Hooper, M.J. (2008) Effects of perchlorate on growth and behavior of a granivorous passerine, the zebra finch (*Taeniopygia guttata*). *Archives of Environmental Contamination and Toxicology*, **54**, 516-24.

Rajapakse, N., Silva, E. & Kortenkamp, A. (2002) Combining xenoestrogens at levels below individual No-observed-effect concentrations dramatically enhances steroid hormone action. *Environmental Health Perspectives*, **110**, 917-921.

Ranciere, F., Lyons, J.G., Loh, V.H., Botton, J., Galloway, T., Wang, T., Shaw, J.E. & Magliano, D.J. (2015) Bisphenol A and the risk of cardiometabolic disorders: a systematic review with meta-analysis of the epidemiological evidence. *Environmental Health*, **14**, 46.

Rand-Weaver, M., Margiotta-Casaluci, L., Patel, A., Panter, G.H., Owen, S.F. & Sumpter, J.P. (2013) The read-across hypothesis and environmental risk assessment of pharmaceuticals. *Environmental Science & Technology*, **47**, 11384-95.

Ratcliffe, D.A. (1958) Broken eggs in peregrine eyries. *British Birds*, **51**, 23-26.

Ratcliffe, D.A. (1960) Broken eggs in the nests of sparrowhawk and golden eagle. *British Birds*, 128-130.

Ratcliffe, D.A. (1967) Decrease in Eggshell Weight in Certain Birds of Prey. *Nature*, **215**, 208-210.

Ratcliffe, D.A. (1970) Changes Attributable to Pesticides in Egg Breakage Frequency and Eggshell Thickness in Some British Birds. *Journal of Applied Ecology*, **7**, 67-115.

Reeder, A.L., Ruiz, M.O., Pessier, A., Brown, L.E., Levengood, J.M., Phillips, C.A., Wheeler, M.B., Warner, R.E. & Beasley, V.R. (2005) Intersexuality and the Cricket Frog Decline: Historic and Geographic Trends. *Environmental Health Perspectives*, **113**, 261-265.

Reeder, A.L., Foley, G.L., Nichols, D.K., Hansen, L.G., Wikoff, B., Faeh, S., Eisold, J., Wheeler, M.B., Warner, R., Murphy, J.E. & Beasley, V.R. (1998) Forms and prevalence of intersexuality and effects of environmental contaminants on sexuality in cricket frogs (*Acris crepitans*). *Environmental Health Perspectives*, **106**, 261-266.

Reeves, C. (2015) Of Frogs & Rhetoric: The Atrazine Wars. *Technical Communication Quarterly*, **24**, 328-348.

Reinhardt, C.D. & Wagner, J.J. (2014) High-dose anabolic implants are not all the same for growth and carcass traits of feedlot steers: A meta-analysis. *Journal of Animal Science*, **92**, 4711-4718.

Renner, R. (2002) Do cattle growth hormones pose an environmental risk? *Environmental Science & Technology*, **36**, 195A-197A.

Rhind, S.M. (2009) Anthropogenic pollutants: a threat to ecosystem sustainability? *Philosophical Transactions of the Royal Society of London B: Biological Sciences*, **364**, 3391-3401.

Rhind, S.M., Kyle, C.E., Telfer, G., Duff, E.I. & Smith, A. (2005) Alkyl phenols and diethylhexyl phthalate in tissues of sheep grazing pastures fertilized with sewage sludge or inorganic fertilizer. *Environmental Health Perspectives*, **113**, 447-453.

Rhomberg, L.R. & Goodman, J.E. (2012) Low-dose effects and nonmonotonic dose-responses of endocrine disrupting chemicals: Has the case been made? *Regulatory Toxicology and Pharmacology*, **64**, 130-133.

Richard, A.M., Judson, R.S., Houck, K.A., Grulke, C.M., Volarath, P., Thillainadarajah, I., Yang, C., Rathman, J., Martin, M.T., Wambaugh, J.F., Knudsen, T.B., Kancherla, J., Mansouri, K., Patlewicz, G., Williams, A.J., Little, S.B., Crofton, K.M. & Thomas, R.S. (2016) ToxCast Chemical Landscape: Paving the Road to 21st Century Toxicology. *Chemical Research in Toxicology*, **29**, 1225-51.

Richter, C.A., Birnbaum, L.S., Farabollini, F., Newbold, R.R., Rubin, B.S., Talsness, C.E., Vandenbergh, J.G., Walser-Kuntz, D.R. & vom Saal, F.S. (2007) *In vivo* effects of bisphenol A in laboratory rodent studies. *Reproductive Toxicology*, **24**, 199-224.

Rietjens, I.M.C.M., Louisse, J. & Beekmann, K. (2017) The potential health effects of dietary phytoestrogens. *British Journal of Pharmacology*, **174**, 1263-1280.

Risebrough, R.W., Rieche, P., Peakall, D.B., Herman, S.G. & Kirven, M.N. (1968) Polychlorinated Biphenyls in the Global Ecosystem. *Nature*, **220**, 1098-1102.

Ritter, R., Scheringer, M., MacLeod, M., Moeckel, C., Jones, K.C. & Hungerbuhler, K. (2011) Intrinsic human elimination half-lives of polychlorinated biphenyls derived from the temporal evolution of cross-sectional biomonitoring data from the United Kingdom. *Environmental Health Perspectives*, **119**, 225-31.

Roach, A.C. & Wilson, S.P. (2009) Ecological impacts of tributyltin on estuarine communities in the Hastings River, NSW Australia. *Marine Pollution Bulletin*, **58**, 1780-1786.

Robinson, R. (2008) For mammals, loss of yolk and gain of milk went hand in hand. *PLoS Biology*, **6**, e77.

Rochester, J.R. & Bolden, A.L. (2015) Bisphenol S and F: A Systematic Review and Comparison of the Hormonal Activity of Bisphenol A Substitutes. *Environmental Health Perspectives*, **123**, 643-650.

Rohr, J.R. & McCoy, K.A. (2010) A qualitative meta-analysis reveals consistent effects of atrazine on freshwater fish and amphibians. *Environ Health Perspect*, **118**, 20-32.

Rohr, J.R., Sesterhenn, T.M. & Stieha, C. (2011) Will climate change reduce the effects of a pesticide on amphibians?: partitioning effects on exposure and susceptibility to contaminants. *Global Change Biology*, **17**, 657-666.

Rohr, J.R., Elskus, A.A., Shepherd, B.S., Crowley, P.H., McCarthy, T.M., Niedzwiecki, J.H., Sager, T., Sih, A. & Palmer, B.D. (2004) Multiple stressors and salamanders: Effects of an herbicide, food limitation, and hydroperiod. *Ecological Applications*, **14**, 1028-1040.

Roos, A.M., Backlin, B.-M.V.M., Helander, B.O., Riget, F.E. & Eriksson, U.C. (2012) Improved reproductive success in otters (*Lutra lutra*), grey seals (*Halichoerus grypus*) and sea eagles (*Haliaeetus albicilla*) from Sweden in relation to concentrations of organochlorine contaminants. *Environmental Pollution*, **170**, 268-275.

Rosner, D. & Markowitz, G. (2013) Persistent pollutants: a brief history of the discovery of the widespread toxicity of chlorinated hydrocarbons. *Environmental Research*, **120**, 126-33.

Ross, P.S. (2006) Fireproof killer whales (*Orcinus orca*): flame-retardant chemicals and the conservation imperative in the charismatic icon of British Columbia, Canada. *Canadian Journal of Fisheries and Aquatic Sciences*, **63**, 224-234.

Ross, P.S., Ellis, G.M., Ikonomou, M.G., Barrett-Lennard, L.G. & Addison, R.F. (2000) High PCB concentrations in free-ranging Pacific killer whales, *Orcinus orca*: Effects of age, sex and dietary preference. *Marine Pollution Bulletin*, **40**, 504-515.

Routledge, E.J. & Sumpter, J.P. (1997) Structural Features of Alkylphenolic Chemicals Associated with Estrogenic Activity. *Journal of Biological Chemistry*, **272**, 3280-3288.

Routledge, E.J., Parker, J., Odum, J., Ashby, J. & Sumpter, J.P. (1998) Some alkyl hydroxy benzoate preservatives (parabens) are estrogenic. *Toxicol Appl Pharmacol*, **153**, 12-9.

Routti, H., Lydersen, C., Hanssen, L. & Kovacs, K.M. (2014) Contaminant levels in the world's northernmost harbor seals (*Phoca vitulina*). *Marine Pollution Bulletin*, **87**, 140-6.

Routti, H., Krafft, B.A., Herzke, D., Eisert, R. & Oftedal, O. (2015) Perfluoroalkyl substances detected in the world's southernmost marine mammal, the Weddell seal (*Leptonychotes weddellii*). *Environmental Pollution*, **197**, 62-67.

Routti, H., Nyman, M., Jenssen, B.M., Backman, C., Koistinen, J. & Gabrielsen, G.W. (2008) Bone-related effects of contaminants in seals may be associated with vitamin D and thyroid hormones. *Environmental Toxicology and Chemistry*, **27**, 873-880.

Rubin, B.S. (2011) Bisphenol A: an endocrine disruptor with widespread exposure and multiple effects. *J Steroid Biochem Mol Biol*, **127**, 27-34.

Ruhl, A.S., Zietzschmann, F., Hilbrandt, I., Meinel, F., Altmann, J., Sperlich, A. & Jekel, M. (2014) Targeted testing of activated carbons for advanced wastewater treatment. *Chemical Engineering Journal*, **257**, 184-190.

Runnalls, T.J., Margiotta-Casaluci, L., Kugathas, S. & Sumpter, J.P. (2010) Pharmaceuticals in the Aquatic Environment: Steroids and Anti-Steroids as High Priorities for Research. *Human and Ecological Risk Assessment: An International Journal*, **16**, 1318-1338.

Runnalls, T.J., Beresford, N., Losty, E., Scott, A.P. & Sumpter, J.P. (2013) Several synthetic progestins with different potencies adversely affect reproduction of fish. *Environmental Science & Technology*, **47**, 2077-84.

Ryan, C., McHugh, B., Boyle, B., McGovern, E., Berube, M., Lopez-Suarez, P., Elfes, C.T., Boyd, D.T., Ylitalo, G.M., Van Blaricom, G.R., Clapham, P.J., Robbins, J., Palsboll, P.J., O'Connor, I. & Berrow, S.D. (2014) Levels of persistent organic pollutants in eastern North Atlantic humpback whales. *Endangered Species Research*, **22**, 213-223.

Rybnikova, V., Usman, M. & Hanna, K. (2016) Removal of PCBs in contaminated soils by means of chemical reduction and advanced oxidation processes. *Environmental Science and Pollution Research*, **23**, 17035-48.

Saaristo, M., Tomkins, P., Allinson, M., Allinson, G. & Wong, B.B.M. (2013) An Androgenic Agricultural Contaminant Impairs Female Reproductive Behaviour in a Freshwater Fish. *Plos One*, **8**

Safford, R.J. & Jones, C.G. (1997) Did organochlorine pesticide use cause declines in Mauritian forest birds? *Biodiversity and Conservation*, **6**, 1445-1451.

Säfholm, M., Ribbenstedt, A., Fick, J. & Berg, C. (2014) Risks of hormonally active pharmaceuticals to amphibians: a growing concern regarding progestagens. *Philosophical Transactions of the Royal Society of London B: Biological Sciences*, **369**

Sagerup, K., Savinov, V., Savinova, T., Kuklin, V., Muir, D.C.G. & Gabrielsen, G.W. (2009) Persistent organic pollutants, heavy metals and parasites in the glaucous gull (*Larus hyperboreus*) on Spitsbergen. *Environmental Pollution*, **157**, 2282-2290.

SAICM (2017) *Strategic approach to international chemicals management*. <http://www.saicm.org/>.

Santillo, D., Johnston, P. & Langston, W.J. (2002) Tributyltin (TBT) antifoulants: a tale of ships, snails and imposex. In: *Late lessons from early warnings: the precautionary principle 1896-2000* (ed. D. Gee). European Environment Agency

Sarradin, P.-M., Lapaquellerie, Y., Astruc, A., Latouche, C. & Astruc, M. (1995) Long term behaviour and degradation kinetics of tributyltin in a marina sediment. *Science of The Total Environment*, **170**, 59-70.

Sass, J.B. & Colangelo, A. (2006) European Union bans atrazine, while the United States negotiates continued use. *International Journal of Occupational and Environmental Health*, **12**, 260-7.

Scheringer, M., Strempel, S., Hukari, S., Ng, C.A., Blepp, M. & Hungerbuhler, K. (2012) How many persistent organic pollutants should we expect? *Atmospheric Pollution Research*, **3**, 383-391.

Scheurer, M., Michel, A., Brauch, H.-J., Ruck, W. & Sacher, F. (2012) Occurrence and fate of the antidiabetic drug metformin and its metabolite guanylurea in the environment and during drinking water treatment. *Water Research*, **46**, 4790-4802.

Schiedek, D., Sundelin, B., Readman, J.W. & Macdonald, R.W. (2007) Interactions between climate change and contaminants. *Marine Pollution Bulletin*, **54**, 1845-56.

Schoenborn, A., Kunz, P. & Koster, M. (2015) Estrogenic activity in drainage water: a field study on a Swiss cattle pasture. *Environmental Sciences Europe*, **27**

Scholz, S. & Mayer, I. (2008) Molecular biomarkers of endocrine disruption in small model fish. *Molecular and Cellular Endocrinology*, **293**, 57-70.

Schork, N.J. (2015) Personalized medicine: Time for one-person trials. *Nature*, **520**, 609-611.

Schwacke, L.H., Zolman, E.S., Balmer, B.C., De Guise, S., George, R.C., Hoguet, J., Hohn, A.A., Kucklick, J.R., Lamb, S., Levin, M., Litz, J.A., McFee, W.E., Place, N.J., Townsend, F.I., Wells, R.S. & Rowles, T.K. (2012) Anaemia, hypothyroidism and immune suppression associated with polychlorinated biphenyl exposure in bottlenose dolphins (*Tursiops truncatus*). *Proceedings of the Royal Society B — Biological Sciences*, **279**, 48-57.

Schwindt, A.R. (2015) Parental effects of endocrine disrupting compounds in aquatic wildlife: Is there evidence of transgenerational inheritance? *General and Comparative Endocrinology*, **219**, 152-164.

Scognamiglio, V., Antonacci, A., Patrolecco, L., Lambreva, M.D., Litescu, S.C., Ghuge, S.A. & Rea, G. (2016) Analytical tools monitoring endocrine disrupting chemicals. *TrAC Trends in Analytical Chemistry*, **80**, 555-567.

Scott, P., Bartkow, M., Blockwell, S., Coleman, H., Khan, S., Lim, R., McDonald, J., Nice, H., Nugegoda, D., Pettigrove, V., Tremblay, L., Warne, M.J. & Leusch, F.L. (2014) An assessment of endocrine activity in Australian rivers using chemical and in vitro analyses. *Environmental Science and Pollution Research*, **21**, 12951-12967.

Segner, H., Navas, J.M., Schäfers, C. & Wenzel, A. (2003) Potencies of estrogenic compounds in *in vitro* screening assays and in life cycle tests with zebrafish *in vivo*. *Ecotoxicology and Environmental Safety*, **54**, 315-322.

Seltenrich, N. (2015) A Hard Nut to Crack: Reducing Chemical Migration in Food-Contact Materials. *Environ Health Perspect*, **123**, A174-9.

Senthilkumar, K., Kannan, K., Subramanian, A. & Tanabe, S. (2001) Accumulation of organochlorine pesticides and polychlorinated biphenyls in sediments, aquatic organisms, birds, bird eggs and bat collected from south India. *Environmental Science and Pollution Research*, **8**, 35-47.

Shannon, M.J. & Unterman, R. (1993) Evaluating bioremediation: distinguishing fact from fiction. *Annual Review of Microbiology*, **47**, 715-38.

Sharma, A.K., Saini, M., Singh, S.D., Prakash, V., Das, A., Bharathi Dasan, R., Pandey, S., Bohara, D., Galligan, T.H., Green, R.E., Knopp, D. & Cuthbert, R.J. (2014) Diclofenac is toxic to the Steppe Eagle *Aquila nipalensis*: widening the diversity of raptors threatened by NSAID misuse in South Asia. *Bird Conservation International*, **24**, 282-286.

Sheahan, D.A., Brighty, G.C., Daniel, M., Kirby, S.J., Hurst, M.R., Kennedy, J., Morris, S., Routledge, E.J., Sumpter, J.P. & Waldock, M.J. (2002a) Estrogenic activity measured in a sewage treatment works treating industrial inputs containing high concentrations of alkylphenolic compounds-A case study. *Environmental Toxicology and Chemistry*, **21**, 507-514.

Sheahan, D.A., Brighty, G.C., Daniel, M., Jobling, S., Harries, J.E., Hurst, M.R., Kennedy, J., Kirby, S.J., Morris, S., Routledge, E.J., Sumpter, J.P. & Waldock, M.J. (2002b) Reduction in the estrogenic activity of a treated sewage effluent discharge to an english river as a result of a decrease in the concentration of industrially derived surfactants. *Environmental Toxicology and Chemistry*, **21**, 515-519.

Shen, O., Wu, W., Du, G., Liu, R., Yu, L., Sun, H., Han, X., Jiang, Y., Shi, W., Hu, W., Song, L., Xia, Y., Wang, S. & Wang, X. (2011) Thyroid Disruption by Di-n-Butyl Phthalate (DBP) and Mono-n-Butyl Phthalate (MBP) in *Xenopus laevis*. *Plos One*, **6**

Shi, Z., Zhang, H., Ding, L., Feng, Y., Xu, M. & Dai, J. (2009) The effect of perfluorododecanonic acid on endocrine status, sex hormones and expression of steroidogenic genes in pubertal female rats. *Reprod Toxicol*, **27**, 352-9.

Short, S., Yang, G., Kille, P. & Ford, A.T. (2014) Vitellogenin is not an appropriate biomarker of feminisation in a crustacean. *Aquatic Toxicology*, **153**, 89-97.

Silbergeld, E.K., Mandrioli, D. & Cranor, C.F. (2015) Regulating chemicals: law, science, and the unbearable burdens of regulation. *Annual Review of Public Health*, **36**, 175-91.

Sindiku, O., Babayemi, J., Osibanjo, O., Schlummer, M., Schluep, M., Watson, A. & Weber, R. (2015) Polybrominated diphenyl ethers listed as Stockholm Convention POPs, other brominated flame retardants and heavy metals in e-waste polymers in Nigeria. *Environmental Science and Pollution Research*, **22**, 14489-501.

Sinkkonen, S. & Paasivirta, J. (2000) Degradation half-life times of PCDDs, PCDFs and PCBs for environmental fate modeling. *Chemosphere*, **40**, 943-949.

Skarphedinsdottir, H., Gunnarsson, K., Gudmundsson, G.A. & Nfon, E. (2010) Bioaccumulation and Biomagnification of Organochlorines in a Marine Food Web at a Pristine Site in Iceland. *Archives of Environmental Contamination and Toxicology*, **58**, 800-809.

Slama, R., Bourguignon, J.P., Demeneix, B., Ivell, R., Panzica, G., Kortenkamp, A. & Zoeller, T. (2016) Scientific Issues Relevant to Setting Regulatory Criteria to Identify Endocrine Disrupting Substances in the European Union. *Environmental Health Perspectives*, **124**, 1497-1503.

Smith, P.J. (1996) Selective decline in imposex levels in the dogwhelk *Lepsiella scobin*a following a ban on the use of TBT antifoulants in New Zealand. *Marine Pollution Bulletin*, **32**, 362-365.

Smith, P.J. & Mcveagh, M. (1991) Widespread Organotin Pollution in New Zealand Coastal Waters as Indicated by Imposex in Dogwhelks. *Marine Pollution Bulletin*, **22**, 409-413.

Smith, S.R. (2009) Organic contaminants in sewage sludge (biosolids) and their significance for agricultural recycling. *Philosophical Transactions of the Royal Society A — Mathematical, Physical and Engineering Sciences*, **367**, 4005-4041.

Smithwick, M., Norstrom, R.J., Mabury, S.A., Solomon, K., Evans, T.J., Stirling, I., Taylor, M.K. & Muir, D.C.G. (2006) Temporal trends of perfluoroalkyl contaminants in polar bears (*Ursus maritimus*) from two locations in the North American Arctic, 1972-2002. *Environmental Science & Technology*, **40**, 1139-1143.

Smithwick, M., Muir, D.C.G., Mabury, S.A., Solomon, K.R., Martin, J.W., Sonne, C., Born, E.W., Letcher, R.J. & Dietz, R. (2005) Perflouroalkyl Contaminants in Liver Tissue from East Greenland Polar Bears (*Ursus maritimus*). *Environmental Toxicology and Chemistry*, **24**, 981.

Soares, A., Guieysse, B., Jefferson, B., Cartmell, E. & Lester, J.N. (2008) Nonylphenol in the environment: a critical review on occurrence, fate, toxicity and treatment in wastewaters. *Environment International*, **34**, 1033-49.

Sohoni, P. & Sumpter, J.P. (1998) Several environmental oestrogens are also anti-androgens. *Journal of Endocrinology*, **158**, 327-339.

Solecki, R., Kortenkamp, A., Bergman, A., Chahoud, I., Degen, G.H., Dietrich, D., Greim, H., Hakansson, H., Hass, U., Husoy, T., Jacobs, M., Jobling, S., Mantovani, A., Marx-Stoelting, P., Piersma, A., Ritz, V., Slama, R., Stahlmann, R., van den Berg, M., Zoeller, R.T. & Boobis, A.R. (2016) Scientific principles for the identification of endocrine-disrupting chemicals: a consensus statement. *Archives of Toxicology*, **91**, 1001-1006.

Solomon, K.R., Baker, D.B., Richards, R.P., Dixon, K.R., Klaine, S.J., La Point, T.W., Kendall, R.J., Weisskopf, C.P., Giddings, J.M., Giesy, J.P., Hall, L.W. & Williams, W.M. (1996) Ecological risk assessment of atrazine in North American surface waters. *Environmental Toxicology and Chemistry*, **15**, 31-76.

Sonne, C. (2010) Health effects from long-range transported contaminants in Arctic top predators: An integrated review based on studies of polar bears and relevant model species. *Environment International*, **36**, 461-491.

Sonne, C., Dietz, R., Born, E.W., Riget, F.F., Kirkegaard, M., Hyldstrup, L., Letcher, R.J. & Derek, C.G.M. (2004) Is Bone Mineral Composition Disrupted by Organochlorines in East Greenland Polar Bears (*Ursus maritimus*)? *Environmental Health Perspectives*, **112**, 1711-1716.

Sonne, C., Leifsson, P.S., Dietz, R., Born, E.W., Letcher, R.J., Kirkegaard, M., Muir, D.C.G., Andersen, L.W., Riget, F.F. & Hyldstrup, L. (2005) Enlarged clitoris in wild polar bears (*Ursus maritimus*) can be misdiagnosed as pseudohermaphroditism. *Science of the Total Environment*, **337**, 45-58.

Sonne, C., Jepson, P.D., Desforges, J.P., Alstrup, A.K.O., Olsen, M.T., Eulaers, I., Hansen, M., Letcher, R.J., McKinney, M.A. & Dietz, R. (2018) Pollution threatens toothed whales. *Science*, **361**, 1208.

Sorensen, J.P., Lapworth, D.J., Nkhuwa, D.C., Stuart, M.E., Gooddy, D.C., Bell, R.A., Chirwa, M., Kabika, J., Liemisa, M., Chibesa, M. & Pedley, S. (2015) Emerging contaminants in urban groundwater sources in Africa. *Water Research*, **72**, 51-63.

Soto, A.M., Sonnenschein, C., Chung, K.L., Fernandez, M.F., Olea, N. & Serrano, F.O. (1995) The E-Screen Assay as a Tool to Identify Estrogens - an Update on Estrogenic Environmental-Pollutants. *Environmental Health Perspectives*, **103**, 113-122.

Sousa, A.C.A., Barroso, C.M., Tanabe, S. & Horiguchi, T. (2010) Involvement of retinoid X receptor in imposex development in Nucella lapillus and Nassarius reticulatus–preliminary results. In: *Interdisciplinary Studies on Environmental Chemistry — Biological Responses to Contaminants* eds. N. Hamamura, S. Suzuki, S. Mendo, C.M. Barroso, H. Iwata and S. Tanabe), pp. 189-196. Terrapub

Sousa, A.C.A., Pastorinho, M.R., Takahashi, S. & Tanabe, S. (2013) Organotin Compounds from Snails to Humans. In: *Pollutant Diseases, Remediation and Recycling* eds. E. Lichtfouse, J. Schwarzbauer and D. Robert). Springer International, Switzerland.

Sousa, A.C.A., Pastorinho, M.R., Takahashi, S. & Tanabe, S. (2014) History on organotin compounds, from snails to humans. *Environmental Chemistry Letters*, **12**, 117-137.

Spearow, J.L. (1999) Genetic Variation in Susceptibility to Endocrine Disruption by Estrogen in Mice. *Science*, **285**, 1259-1261.

Sprague, B.L., Trentham-Dietz, A. & Cronin, K.A. (2012) A sustained decline in postmenopausal hormone use: results from the National Health and Nutrition Examination Survey, 1999-2010. *Obstet Gynecol*, **120**, 595-603.

Stockholm Convention (2008a) *The Persistent Organic Pollutants*. <http://chm.pops.int/TheConvention/ThePOPs/tabid/673/Default.aspx>.

Stockholm Convention (2008b) *Overview*. [http://chm.pops.int/TheConvention/Overview/tabid/3351/Default.aspx#](http://chm.pops.int/TheConvention/Overview/tabid/3351/Default.aspx).

Stockholm Convention (2008c) *History of the negotiations of the Stockholm Convention*, <http://chm.pops.int/TheConvention/Overview/History/Overview/tabid/3549/Default.aspx>.

Stockholm Convention (2008d) *All POPs listed in the Stockholm Convention*. <http://chm.pops.int/TheConvention/ThePOPs/ListingofPOPs/tabid/2509/Default.aspx>.

Stockholm Convention (2008e) *Global Monitoring Plan*. <http://chm.pops.int/Implementation/GlobalMonitoringPlan/Overview/tabid/83/Default.aspx>.

Stockholm County Council (2014-2015) Environmentally classified pharmaceuticals. In, Stockholm, Sweden.

Stockin, K.A., Law, R.J., Roe, W.D., Meynier, L., Martinez, E., Duignan, P.J., Bridgen, P. & Jones, B. (2010) PCBs and organochlorine pesticides in Hector's (*Cephalorhynchus hectori hectori*) and Maui's (*Cephalorhynchus hectori maui*) dolphins. *Marine Pollution Bulletin*, **60**, 834-42.

Stoker, C., Zayas, M.A., Ferreira, M.A., Durando, M., Galoppo, G.H., Rodriguez, H.A., Repetti, M.R., Beldomenico, H.R., Caldini, E.G., Luque, E.H. & Munoz-de-Toro, M. (2013) The eggshell features and clutch viability of the broad-snouted caiman (*Caiman latirostris*) are associated with the egg burden of organochlorine compounds. *Ecotoxicology and Environmental Safety*, **98**, 191-195.

Strand, J., Glahder, C.M. & Asmund, G. (2006) Imposex occurrence in marine whelks at a military facility in the high Arctic. *Environmental Pollution*, **142**, 98-102.

Straub, J.O. (2016) Reduction in the environmental exposure of pharmaceuticals through diagnostics, Personalised Healthcare and other approaches. A mini review and discussion paper. *Sustainable Chemistry and Pharmacy*, **3**, 1-7.

Stuart, M., Lapworth, D., Crane, E. & Hart, A. (2012) Review of risk from potential emerging contaminants in UK groundwater. *Science of the Total Environment*, **416**, 1-21.

Stuart-Smith, S.J. & Jepson, P.D. (2017) Persistent threats need persistent counteraction: Responding to PCB pollution in marine mammals. *Marine Policy*, **84**, 69-75.

Sudharshan, S., Naidu, R., Mallavarapu, M. & Bolan, N. (2012) DDT remediation in contaminated soils: a review of recent studies. *Biodegradation*, **23**, 851-63.

Sumpter, J.P. (2009) Protecting aquatic organisms from chemicals: the harsh realities. *Philosophical Transactions of the Royal Society A — Mathematical, Physical and Engineering Sciences*, **367**, 3877-3894.

Sumpter, J.P. & Jobling, S. (1995) Vitellogenesis as a Biomarker for Estrogenic Contamination of the Aquatic Environment. *Environmental Health Perspectives*, **103**, 173-178.

Sumpter, J.P. & Johnson, A. (2005) Lessons from Endocrine Disruption and Their Application to Other Issues Concerning Trace Organics in the Aquatic Environment. *Environmental Science & Technology*, **39**, 4321-4332.

Sumpter, J.P. & Johnson, A.C. (2008) 10th Anniversary Perspective: Reflections on endocrine disruption in the aquatic environment: from known knowns to unknown unknowns (and many things in between). *Journal of Environmental Monitoring*, **10**, 1476-85.

Sumpter, J.P., Donnachie, R.L. & Johnson, A.C. (2014) The apparently very variable potency of the anti-depressant fluoxetine. *Aquatic Toxicology*, **151**, 57-60.

Sumpter, J.P., Scott, A.P. & Katsiadaki, I. (2016) Comments on Niemuth, N.J. and Klaper, R.D. 2015. Emerging wastewater contaminant metformin causes intersex and reduced fecundity in fish. Chemosphere 135, 38-45. *Chemosphere*, **165**, 566-569.

Swan, G., Naidoo, V., Cuthbert, R., Green, R.E., Pain, D.J., Swarup, D., Prakash, V., Taggart, M., Bekker, L., Das, D., Diekmann, J., Diekmann, M., Killian, E., Meharg, A., Patra, R.C., Saini, M. & Wolter, K. (2006a) Removing the threat of diclofenac to critically endangered Asian vultures. *Plos Biology*, **4**, e66.

Swan, G.E., Cuthbert, R., Quevedo, M., Green, R.E., Pain, D.J., Bartels, P., Cunningham, A.A., Duncan, N., Meharg, A.A., Oaks, J.L., Parry-Jones, J., Shultz, S., Taggart, M.A., Verdoorn, G. & Wolter, K. (2006b) Toxicity of diclofenac to Gyps vultures. *Biology Letters*, **2**, 279-82.

Takahashi, S., Tanabe, S. & Kawaguchi, K. (2000) Organochlorine and butyltin residues in mesopelagic myctophid fishes from the western North Pacific. *Environmental Science & Technology*, **34**, 5129-5136.

Tartu, S., Gabrielsen, G.W., Blevin, P., Ellis, H., Bustnes, J.O., Herzke, D. & Chastel, O. (2014a) Endocrine and Fitness Correlates of Long-Chain Perfluorinated Carboxylates Exposure in Arctic Breeding Black-Legged Kittiwakes. *Environmental Science & Technology*, **48**, 13504-13510.

Tartu, S., Angelier, F., Herzke, D., Moe, B., Bech, C., Gabrielsen, G.W., Bustnes, J.O. & Chastel, O. (2014b) The stress of being contaminated? Adrenocortical function and reproduction in relation to persistent organic pollutants in female black legged kittiwakes. *Science of the Total Environment*, **476**, 553-560.

Tartu, S., Lendvai, A.Z., Blevin, P., Herzke, D., Bustamante, P., Moe, B., Gabrielsen, G.W., Bustnes, J.O. & Chastel, O. (2015a) Increased adrenal responsiveness and delayed hatching date in relation to polychlorinated biphenyl exposure in Arctic-breeding black-legged kittiwakes (*Rissa tridactyla*). *General and Comparative Endocrinology*, **219**, 165-172.

Tartu, S., Aars, J., Andersen, M., Polder, A., Bourgeon, S., Merkel, B., Lowther, A.D., Bytingsvik, J., Welker, J.M., Derocher, A.E., Jenssen, B.M. & Routti, H. (2018) Choose Your Poison-Space-Use Strategy Influences Pollutant Exposure in Barents Sea Polar Bears. *Environmental Science and Technology*, **52**, 3211-3221.

Tartu, S., Angelier, F., Bustnes, J.O., Moe, B., Hanssen, S.A., Herzke, D., Gabrielsen, G.W., Verboven, N., Verreault, J., Labadie, P., Budzinski, H., Wingfield, J.C. & Chastel, O. (2015b) Polychlorinated biphenyl exposure and corticosterone levels in seven polar seabird species. *Environmental Pollution*, **197**, 173-180.

Tavera-Mendoza, L., Ruby, S., Brousseau, P., Fournier, M., Cyr, D. & Marcogliese, D. (2002) Response of the amphibian tadpole (*Xenopus laevis*) to atrazine during sexual differentiation of the testis. *Environmental Toxicology and Chemistry*, **21**, 527-31.

Taylor, M.D. & Johnson, D.D. (2016) Preliminary investigation of perfluoroalkyl substances in exploited fishes of two contaminated estuaries. *Marine pollution bulletin*, **111**, 509-13.

ter Laak, T.L., Kooij, P.J.F., Tolkamp, H. & Hofman, J. (2014) Different compositions of pharmaceuticals in Dutch and Belgian rivers explained by consumption patterns and treatment efficiency. *Environmental Science and Pollution Research*, **21**, 12843-12855.

Ternes, T.A., Joss, A. & Siegrist, H. (2004) Peer Reviewed: Scrutinizing Pharmaceuticals and Personal Care Products in Wastewater Treatment. *Environmental Science & Technology*, **38**, 392A-399A.

Teuten, E.L., Saquing, J.M., Knappe, D.R.U., Barlaz, M.A., Jonsson, S., Björn, A., Rowland, S.J., Thompson, R.C., Galloway, T.S., Yamashita, R., Ochi, D., Watanuki, Y., Moore, C., Viet, P.H., Tana, T.S., Prudente, M., Boonyatumanond, R., Zakaria, M.P., Akkhavong, K., Ogata, Y., Hirai, H., Iwasa, S., Mizukawa, K., Hagino, Y., Imamura, A., Saha, M. & Takada, H. (2009) Transport and release of chemicals from plastics to the environment and to wildlife. *Philosophical Transactions of the Royal Society B — Biological Sciences*, **364**, 2027-2045.

Theodorakis, C.W., Rinchard, J., Carr, J.A., Park, J.W., McDaniel, L., Liu, F. & Wages, M. (2006) Thyroid endocrine disruption in stonerollers and cricket frogs from perchlorate-contaminated streams in east-central Texas. *Ecotoxicology*, **15**, 31-50.

Theodoris, P. (2008) The Consequences of the ‘cut off’ Criteria for Pesticides: Agronomic and Financial Aspects *European Parliament: Policy Department B, Structural and Cohension Policies*, IP/B/AGRI/IC/2008_166, <http://www.europarl.europa.eu/RegData/etudes/note/join/2008/408963/IPOL-AGRI_NT%282008%29408963_EN.pdf>.

Thompson, R.C., Moore, C.J., vom Saal, F.S. & Swan, S.H. (2009) Plastics, the environment and human health: current consensus and future trends. *Philosophical Transactions of the Royal Society of London — Biological Sciences*, **364**, 2153-66.

Thornton, J.W. (2007) What Can We Do About Endocrine-Disrupting Chemicals? *Endocrine-Disrupting Chemicals: From Basic Research to Clinical Practice* (ed. by A.C. Gore). Humana Press, Totowa, NJ.

Thorpe, K.L., Cummings, R.I., Hutchinson, T.H., Scholze, M., Brighty, G., Sumpter, J.P. & Tyler, C.R. (2003) Relative potencies and combination effects of steroidal estrogens in fish. *Environmental Science & Technology*, **37**, 1142-1149.

Thrupp, T.J., Runnalls, T.J., Scholze, M., Kugathas, S., Kortenkamp, A. & Sumpter, J.P. (2018) The consequences of exposure to mixtures of chemicals: Something from ‘nothing’ and ‘a lot from a little’ when fish are exposed to steroid hormones. *Science of The Total Environment*, **619-620**, 1482-1492.

Tietge, J.E., Holcombe, G.W., Flynn, K.M., Kosian, P.A., Korte, J.J., Anderson, L.E., Wolf, D.C. & Degitz, S.J. (2005) Metamorphic Inhibition of *Xenopus laevis* by Sodium Perchlorate: Effects on Development and Thyroid Histology. *Environmental Toxicology and Chemistry*, **24**, 926.

Toft, G., Edwards, T.M., Baatrup, E. & Guillette, L.J. (2003) Disturbed sexual characteristics in male mosquitofish (*Gambusia holbrooki*) from a lake contaminated with endocrine disruptors. *Environmental Health Perspectives*, **111**, 695-701.

Tomkins, P., Saaristo, M., Allinson, M. & Wong, B.B.M. (2016) Exposure to an agricultural contaminant, 17 beta-trenbolone, impairs female mate choice in a freshwater fish. *Aquatic Toxicology*, **170**, 365-370.

Tomza-Marciniak, A., Marciniak, A., Pilarczyk, B., Prokulewicz, A. & Bakowska, M. (2014) Interspecies comparison of chlorinated contaminant concentrations and profiles in wild terrestrial mammals from northwest Poland. *Arch Environ Contam Toxicol*, **66**, 491-503.

Trasande, L., Zoeller, R.T., Hass, U., Kortenkamp, A., Grandjean, P., Myers, J.P., DiGangi, J., Hunt, P.M., Rudel, R., Sathyanarayana, S., Bellanger, M., Hauser, R., Legler, J., Skakkebaek, N.E. & Heindel, J.J. (2016) Burden of disease and costs of exposure to endocrine disrupting chemicals in the European Union: an updated analysis. *Andrology*, **4**, 565-72.

Trautwein, C., Berset, J.-D., Wolschke, H. & Kümmerer, K. (2014) Occurrence of the antidiabetic drug Metformin and its ultimate transformation product Guanylurea in several compartments of the aquatic cycle. *Environment international*, **70**, 203-212.

Tremblay, L.A., Gadd, J.B. & Northcott, G.L. (2018) Steroid estrogens and estrogenic activity are ubiquitous in dairy farm watersheds regardless of effluent management practices. *Agriculture, Ecosystems & Environment*, **253**, 48-54.

Trumble, S.J., Robinson, E.M., Berman-Kowalewski, M., Potter, C.W. & Usenko, S. (2013) Blue whale earplug reveals lifetime contaminant exposure and hormone profiles. *Proceedings of the National Academy of Sciences of the United States of America*, **110**, 16922-16926.

Trumpolt, C.W., Crain, M., Cullison, G.D., Flanagan, S.J.P., Siegel, L. & Lathrop, S. (2005) Perchlorate: Sources, uses, and occurrences in the environment. *Remediation Journal*, **16**, 65-89.

Tyler, C.R. & Jobling, S. (2008) Roach, Sex, and Gender-Bending Chemicals: The Feminization of Wild Fish in English Rivers. *BioScience*, **58**, 1051-1059.

Tyler, C.R. & Filby, A.L. (2011) Feminized Fish, Environmental Estrogens, and Wastewater Effluents in English Rivers. *Wildlife Ecotoxicology: Forensic Approaches* (ed. by J.E. Elliott, C.A. Bishop and C.A. Morrissey), pp. 383-412.

Tyler, C.R., Jobling, S. & Sumpter, J.P. (1998) Endocrine disruption in wildlife: A critical review of the evidence. *Critical Reviews in Toxicology*, **28**, 319-361.

Tyler, C.R., van der Eerden, B., Jobling, S., Panter, G. & Sumpter, J.P. (1996) Measurement of vitellogenin, a biomarker for exposure to oestrogenic chemicals, in a wide variety of cyprinid fish. *Journal of Comparative Physiology B — Biochemical Systemic and Environmental Physiology*, **166**, 418-426.

UK Government (2015) *Dispose of waste containing persistent organic pollutants (POPs)*. <https://www.gov.uk/guidance/dispose-of-waste-containing-persistent-organic-pollutants-pops>.

UK WIR (2017) *The Chemical Investigations Programme Phase 2, 2015-2020 – Introduction*. Available at: <https://www.ukwir.org/the-chemicals-investigation-programme> (accessed

UNEP Chemicals and Waste Branch (2016) Consolidated assessment of efforts made toward the elimination of polychlorinated biphenyls. In. UNEP, Switzerland.

United Nations Department of Economic and Social Affairs (2015) Trends in Contraceptive Use Worldwide 2015 (ST/ESA/SER.A/349). In, New York, USA.

United Nations Environment Programme & The International Panel on Chemical Pollution (2016) Overview Report I: A Compilation of Lists of Chemicals Recognised as Endocrine Disrupting Chemicals (EDCs) or Suggested as Potential EDCs. In:

United Nations Population Fund (2018) *World population trends*. Available at: <https://www.unfpa.org/world-population-trends> (accessed

Urbansky, E.T., Brown, S.K., Magnuson, M.L. & Kelty, C.A. (2001) Perchlorate levels in samples of sodium nitrate fertilizer derived from Chilean caliche. *Environmental Pollution*, **112**, 299-302.

US EPA (2017a) *Toxicity ForeCaster (ToxCast™) Data*. <https://www.epa.gov/chemical-research/toxicity-forecaster-toxcasttm-data>.

US EPA (2017b) *Superfund*. <https://www.epa.gov/superfund>.

US EPA (2017c) *Endocrine Disruptor Screening Program (EDSP) Overview*. <https://www.epa.gov/endocrine-disruption/endocrine-disruptor-screening-program-edsp-overview>.

Usman, A. & Ahmad, M. (2016) From BPA to its analogues: Is it a safe journey? *Chemosphere*, **158**, 131-142.

van Aerle, R., Nolanusan, M., Jobling, S., Christiansen, L.B., Sumpter, J.P. & Tyler, C.R. (2001) Sexual disruption in a second species of wild cyprinid fish (the gudgeon, *Gobio gobio*) in United Kingdom Freshwaters. *Environmental Toxicology and Chemistry*, **20**, 2841-2847.

van den Berg, H. (2009) Global status of DDT and its alternatives for use in vector control to prevent disease. *Environmental Health Perspectives*, **117**, 1656-63.

Van den Berg, M., Birnbaum, L., Bosveld, A.T., Brunstrom, B., Cook, P., Feeley, M., Giesy, J.P., Hanberg, A., Hasegawa, R., Kennedy, S.W., Kubiak, T., Larsen, J.C., van Leeuwen, F.X., Liem, A.K., Nolt, C., Peterson, R.E., Poellinger, L., Safe, S., Schrenk, D., Tillitt, D., Tysklind, M., Younes, M., Waern, F. & Zacharewski, T. (1998) Toxic equivalency factors (TEFs) for PCBs, PCDDs, PCDFs for humans and wildlife. *Environmental Health Perspectives*, **106**, 775-92.

van den Heuvel, M.R. (2010) Recent Progress in Understanding the Causes of Endocrine Disruption Related to Pulp and Paper Mill Effluents. *Water Quality Research Journal of Canada*, **45**, 137-144.

Van Der Kraak, G.J., Hosmer, A.J., Hanson, M.L., Kloas, W. & Solomon, K.R. (2014) Effects of Atrazine in Fish, Amphibians, and Reptiles: An Analysis Based on Quantitative Weight of Evidence. *Critical Reviews in Toxicology*, **44**, 1-66.

van Nuijs, A.L.N., Covaci, A., Beyers, H., Bervoets, L., Blust, R., Verpooten, G., Neels, H. & Jorens, P.G. (2015) Do concentrations of pharmaceuticals in sewage reflect prescription figures? *Environmental Science and Pollution Research*, **22**, 9110-9118.

Van Schmidt, N.D., Cary, T.L., Ortiz-Santaliestra, M.E. & Karasov, W.H. (2012) Effects of chronic polybrominated diphenyl ether exposure on gonadal development in the northern leopard frog, *Rana pipiens*. *Environmental Toxicology and Chemistry*, **31**, 347-354.

Vandenberg, L.N. (2014) Low-Dose Effects of Hormones and Endocrine Disruptors. *Endocrine Disrupters* (ed. by G. Litwack), pp. 129-165.

Vandenberg, L.N. (2015) Chapter 7: Nonmonotonic responses in endocrine disruption. *Endocrine disruption and human health* (ed. by P.D. Darbre). Elsevier, London.

Vandenberg, L.N., Luthi, D. & Quinerly, D.A. (2015) Plastic bodies in a plastic world: multi-disciplinary approaches to study endocrine disrupting chemicals. *Journal of Cleaner Production*,

Vandenberg, L.N., Maffini, M.V., Sonnenschein, C., Rubin, B.S. & Soto, A.M. (2009) Bisphenol-A and the Great Divide: A Review of Controversies in the Field of Endocrine Disruption. *Endocrine Reviews*, **30**, 75-95.

Vandenberg, L.N., Welshons, W.V., Vom Saal, F.S., Toutain, P.L. & Myers, J.P. (2014) Should oral gavage be abandoned in toxicity testing of endocrine disruptors? *Environmental Health*, **13**, 46.

Vandenberg, L.N., Colborn, T., Hayes, T.B., Heindel, J.J., David R. Jacobs, J., Lee, D.-H., Shioda, T., Soto, A.M., vom Saal, F.S., Welshons, W.V., Zoeller, R.T. & Myers, J.P. (2012) Hormones and Endocrine-Disrupting Chemicals: Low-Dose Effects and Nonmonotonic Dose Responses. *Endocrine Reviews*, **33**, 378-455.

Vandenberg, L.N., Agerstrand, M., Beronius, A., Beausoleil, C., Bergman, A., Bero, L.A., Bornehag, C.G., Boyer, C.S., Cooper, G.S., Cotgreave, I., Gee, D., Grandjean, P., Guyton, K.Z., Hass, U., Heindel, J.J., Jobling, S., Kidd, K.A., Kortenkamp, A., Macleod, M.R., Martin, O.V., Norinder, U., Scheringer, M., Thayer, K.A., Toppari, J., Whaley, P., Woodruff, T.J. & Ruden, C. (2016) A proposed framework for the systematic review and integrated assessment (SYRINA) of endocrine disrupting chemicals. *Environmental Health*, **15**, 74.

Veldhoen, N., Skirrow, R.C., Osachoff, H., Wigmore, H., Clapson, D.J., Gunderson, M.P., Van Aggelen, G. & Helbing, C.C. (2006) The bactericidal agent triclosan modulates thyroid hormone-associated gene expression and disrupts postembryonic anuran development. *Aquatic Toxicology*, **80**, 217-27.

Verbruggen, B., Gunnarsson, L., Kristiansson, E., Osterlund, T., Owen, S.F., Snape, J.R. & Tyler, C.R. (2018) ECOdrug: a database connecting drugs and conservation of their targets across species. *Nucleic Acids Research*, **46**, D930-D936.

Verlicchi, P. & Zambello, E. (2015) Pharmaceuticals and personal care products in untreated and treated sewage sludge: Occurrence and environmental risk in the case of application on soil - A critical review. *Science of the Total Environment*, **538**, 750-67.

Verlicchi, P., Al Aukidy, M., Galletti, A., Petrovic, M. & Barcelo, D. (2012) Hospital effluent: investigation of the concentrations and distribution of pharmaceuticals and environmental risk assessment. *Science of the Total Environment*, **430**, 109-18.

Verreault, J., Letcher, R.J., Ropstad, E., Dahl, E. & Gabrielsen, G.W. (2006) Organohalogen contaminants and reproductive hormones in incubating glaucous gulls (*Larus hyperboreus*) from the Norwegian Arctic. *Environmental Toxicology and Chemistry*, **25**, 2990-2996.

Verreault, J., Helgason, L.B., Gabrielsen, G.W., Dam, M. & Braune, B.M. (2013) Contrasting retinoid and thyroid hormone status in differentially-contaminated northern fulmar colonies from the Canadian Arctic, Svalbard and the Faroe Islands. *Environment International*, **52**, 29-40.

Veterinary Medicines Directorate (2014) Code of Practice on the responsible use of animal medicines on the farm. In:

Villanger, G.D., Lydersen, C., Kovacs, K.M., Lie, E., Skaare, J.U. & Jenssen, B.M. (2011a) Disruptive effects of persistent organohalogen contaminants on thyroid function in white whales (*Delphinapterus leucas*) from Svalbard. *Science of the Total Environment*, **409**, 2511-2524.

Villanger, G.D., Jenssen, B.M., Fjeldberg, R.R., Letcher, R.J., Muir, D.C.G., Kirkegaard, M., Sonne, C. & Dietz, R. (2011b) Exposure to mixtures of organohalogen contaminants and associative interactions with thyroid hormones in East Greenland polar bears (*Ursus maritimus*). *Environment International*, **37**, 694-708.

Vos, J.G., Dybing, E., Greim, H.A., Ladefoged, O., Lambre, C., Tarazona, J.V., Brandt, I. & Vethaak, A.D. (2000) Health effects of endocrine-disrupting chemicals on wildlife, with special reference to the European situation. *Critical Reviews in Toxicology*, **30**, 71-133.

Wade, T.L., Chambers, L., Gardinali, P.R., Sericano, J., Jackson, T.J., Tarpley, R.J. & Suydam, R. (1997) Toxaphene, PCB, DDT, and chlordane analyses of Beluga Whale Blubber. *Chemosphere*, **34**, 1351-1357.

Walker, L.A., Shore, R.F., Turk, A., Pereira, M.G. & Best, J. (2008) The Predatory Bird Monitoring Scheme: Identifying Chemical Risks to Top Predators in Britain. *AMBIO*, **37**, 466-471.

Walsh, C.L., Blenkinsop, S., Fowler, H.J., Burton, A., Dawson, R.J., Glenis, V., Manning, L.J., Jahanshahi, G. & Kilsby, C.G. (2016) Adaptation of water resource systems to an uncertain future. *Hydrology and Earth System Sciences*, **20**, 1869-1884.

Walters, E., McClellan, K. & Halden, R.U. (2010) Occurrence and loss over three years of 72 pharmaceuticals and personal care products from biosolids-soil mixtures in outdoor mesocosms. *Water Research*, **44**, 6011-20.

Wang, H., Hwang, J., Huang, J., Xu, Y., Yu, G., Li, W., Zhang, K., Liu, K., Cao, Z., Ma, X., Wei, Z. & Wang, Q. (2017) Mechanochemical remediation of PCB contaminated soil. *Chemosphere*, **168**, 333-340.

Wang, J., Caccamise, S.A.L., Woodward, L.A. & Li, Q.X. (2015) Polychlorinated Biphenyls in the Plasma and Preen Oil of Black-Footed Albatross (*Diomedea nigripes*) Chicks and Adults on Midway Atoll, North Pacific Ocean. *Plos One*, **10**

Wang, J., Zhang, Y., Zhang, F., Yeung, L.W.Y., Taniyasu, S., Yamazaki, E., Wang, R., Lam, P.K.S., Yamashita, N. & Dai, J. (2013) Age- and gender-related accumulation of perfluoroalkyl substances in captive Chinese alligators (*Alligator sinensis*). *Environmental Pollution*, **179**, 61-67.

Wania, F. & Mackay, D. (1996) Tracking the distribution of persistent organic pollutants. *Environmental Science & Technology*, **30**, 390A-6A.

Watanabe, H., Horie, Y., Takanobu, H., Koshio, M., Iguchi, T. & Tatarazako, N. (2017) Medaka Extended One-Generation Reproduction Test (Meogrt) Evaluating 4-Nonylphenol. *Environmental Toxicology and Chemistry*,

Watanabe, N., Sakai, S.-I. & Takatsuki, H. (1995) Release and degradation half lives of tributyltin in sediment. *Chemosphere*, **31**, 2809-2816.

Watson, R. (2016) European Commission tables scientific criteria for endocrine disruptors. *British Medical Journal*, **353**, i3459.

Weber, R., Watson, A., Forter, M. & Oliaei, F. (2011) Persistent organic pollutants and landfills - a review of past experiences and future challenges. *Waste Management Research*, **29**, 107-21.

Weetman, A.P. (2010) The thyroid gland and disorders of thyroid function. In: *Oxford Textbook of Medicine* eds. D.A. Warrell, T.M. Cox and J.D. Firth), pp. 1826-1844. Oxford University Press, United Kingdom.

Weinhold, B. (2009) Melting Glaciers Release Frozen Toxicants. *Environmental Health Perspectives*, **117**, A538-A538.

Weltje, L. & Sumpter, J.P. (2017) What Makes a Concentration Environmentally Relevant? Critique and a Proposal. *Environmental Science & Technology*, **51**, 11520-11521.

Wennmalm, A. & Gunnarsson, B. (2009) Pharmaceutical management through environmental product labeling in Sweden. *Environment International*, **35**, 775-7.

Weseloh, D.V., Teeple, S.M. & Gilbertson, M. (1983) Double-crested Cormorants of the Great Lakes: egg-laying parameters, reproductive failure, and contaminant residues in eggs, Lake Huron 1972–1973. *Canadian Journal of Zoology*, **61**, 427-436.

Weseloh, D.V., Ewins, P.J., Struger, J., Mineau, P., Bishop, C.A., Postupalsky, S. & Ludwig, J.P. (1995) Double-crested Cormorants of the Great Lakes: Changes in population size, breeding distribution and reproductive output between 1913 and 1991. *Colonial Waterbirds*, **18**, 48-59.

Westra, S., Fowler, H.J., Evans, J.P., Alexander, L.V., Berg, P., Johnson, F., Kendon, E.J., Lenderink, G. & Roberts, N.M. (2014) Future changes to the intensity and frequency of short-duration extreme rainfall. *Reviews of Geophysics*, **52**, 522-555.

Weybridge Report (1996) European Workshop on the Impact of Endocrine Disrupters on Human Health and Wildlife. In: (ed. Environment and climate research programme of dg xii of the european commission), Weybridge UK.

Weybridge+15 (2012) The impacts of endocrine disrupters on wildlife, people and their environments. The Weybridge+15 (1996-2011) report. *EEA Technical Report*, **2**, 1-112.

White, R., Jobling, S., Hoare, S.A., Sumpter, J.P. & Parker, M.G. (1994) Environmentally persistent alkylphenolic compounds are estrogenic. *Endocrinology*, **135**, 175-82.

Wilbur, H.M. & Collins, J.P. (1973) Ecological Aspects of Amphibian Metamorphosis: Nonnormal distributions of competitive ability reflect selection for facultative metamorphosis. *Science*, **182**, 1305-14.

Williams, R.J., Churchley, J.H., Kanda, R. & Johnson, A.C. (2012) Comparing predicted against measured steroid estrogen concentrations and the associated risk in two United Kingdom river catchments. *Environmental Toxicology and Chemistry*, **31**, 892-8.

Williams, R.J., Keller, V.D., Johnson, A.C., Young, A.R., Holmes, M.G., Wells, C., Gross-Sorokin, M. & Benstead, R. (2009) A national risk assessment for intersex in fish arising from steroid estrogens. *Environmental Toxicology and Chemistry*, **28**, 220-30.

Windal, I., Denison, M.S., Birnbaum, L.S., Van Wouwe, N., Baeyens, W. & Goeyens, L. (2005) Chemically activated luciferase gene expression (CALUX) cell bioassay analysis for the estimation of dioxin-like activity: Critical parameters of the CALUX procedure that impact assay results. *Environmental Science & Technology*, **39**, 7357-7364.

WingSpread Consensus Statement (1995) Statement from the Work Session on Environmentally induced alteration in development: a focus on wildlife. *Environmental Health Perspectives*, **103 Suppl. 4**, 3-5.

Wolff, J. (1998) Perchlorate and the thyroid gland. *Pharmacological Review*, **50**, 89-105.

Wolkers, H., Corkeron, P.T., Van Parijs, S.M., Simila, T. & Van Bavel, B. (2007) Accumulation and transfer of contaminants in killer whales (*Orcinus orca*) from Norway: Indications for contaminant metabolism. *Environmental Toxicology and Chemistry*, **26**, 1582-1590.

Wolkers, J., Burkow, I.C., Lydersen, C., Dahle, S., Monshouwer, M. & Witkamp, R.F. (1998) Congener specific PCB and polychlorinated camphene (toxaphene) levels in Svalbard ringed seals (*Phoca hispida*) in relation to sex, age, condition and cytochrome P450 enzyme activity. *Science of The Total Environment*, **216**, 1-11.

Wolschke, H., Meng, X.-Z., Xie, Z., Ebinghaus, R. & Cai, M. (2015) Novel flame retardants (N-FRs), polybrominated diphenyl ethers (PBDEs) and dioxin-like polychlorinated biphenyls (DL-PCBs) in fish, penguin, and skua from King George Island, Antarctica. *Marine Pollution Bulletin*, **96**, 513-518.

Wong, M.H., Wu, S.C., Deng, W.J., Yu, X.Z., Luo, Q., Leung, A.O., Wong, C.S., Luksemburg, W.J. & Wong, A.S. (2007) Export of toxic chemicals - a review of the case of uncontrolled electronic-waste recycling. *Environmental Pollution*, **149**, 131-40.

Woodward, A.R., Percival, H.F., Rauschenberger, R.H., Gross, T.S., Rice, K.G. & Conrow, R. (2011) Abnormal Alligators and Organochlorine Pesticides in Lake Apopka, Florida. *Wildlife Ecotoxicology: Forensic Approaches* (ed. by J.E. Elliott, C.A. Bishop and C.A. Morrissey), pp. 153-187.

World Health Organization (1993) *International Programme on Chemical Safety, Environmental Health Criteria 155, Biomarkers and Risk Assessment: Concepts and Principles*. <http://www.inchem.org/documents/ehc/ehc/ehc155.htm>.

World Health Organization (2011) *The use of DDT in malaria vector control. WHO position statement*.

World Health Organization (2014) *Global status report on noncommunicable diseases*. <http://apps.who.int/iris/bitstream/10665/148114/1/9789241564854_eng.pdf?ua=1>.

World Health Organization (2016) *Global Report on Diabetes*. <http://www.who.int/diabetes/global-report/en/>.

Wren, C.D. (1991) Cause-effect linkages between chemicals and populations of mink (Mustela vison) and otter (Lutra canadensis) in the Great Lakes basin. *J Toxicol Environ Health*, **33**, 549-85.

Wu, J.-P., Luo, X.-J., Zhang, Y., Chen, S.-J., Mai, B.-X., Guan, Y.-T. & Yang, Z.-Y. (2009) Residues of Polybrominated Diphenyl Ethers in Frogs (*Rana limnocharis*) from a Contaminated Site, South China: Tissue Distribution, Biomagnification, and Maternal Transfer. *Environmental Science & Technology*, **43**, 5212-5217.

Xu, N., Chen, P., Liu, L., Zeng, Y., Zhou, H. & Li, S. (2014) Effects of combined exposure to 17 alpha-ethynylestradiol and dibutyl phthalate on the growth and reproduction of adult male zebrafish (*Danio rerio*). *Ecotoxicology and Environmental Safety*, **107**, 61-70.

Yang, C.Z., Yaniger, S.I., Jordan, V.C., Klein, D.J. & Bittner, G.D. (2011) Most Plastic Products Release Estrogenic Chemicals: A Potential Health Problem that Can Be Solved. *Environmental Health Perspectives*, **119**, 989-996.

Yarsan, E. & Yipe, M. (2013) The Important Terms of Marine Pollution "Biomarkers and Biomonitoring,Bioaccumulation, Bioconcentration, Biomagnification". *Journal of Molecular Biomarkers & Diagnosis*, **s1**

Yohannes, Y.B., Ikenaka, Y., Nakayama, S.M. & Ishizuka, M. (2014) Organochlorine pesticides in bird species and their prey (fish) from the Ethiopian Rift Valley region, Ethiopia. *Environmental Pollution*, **192**, 121-8.

Yoo, H., Kannan, K., Kim, S.K., Lee, K.T., Newsted, J.L. & Giesy, J.P. (2008) Perfluoroalkyl acids in the egg yolk of birds from Lake Shihwa, Korea. *Environmental Science & Technology*, **42**, 5821-5827.

Yost, A.T., Thornton, L.M., Venables, B.J. & Sellin Jeffries, M.K. (2016) Dietary exposure to polybrominated diphenyl ether 47 (BDE-47) inhibits development and alters thyroid hormone-related gene expression in the brain of *Xenopus laevis* tadpoles. *Environmental toxicology and pharmacology*, **48**, 237-244.

Yu, C.P., Deeb, R.A. & Chu, K.H. (2013) Microbial degradation of steroidal estrogens. *Chemosphere*, **91**, 1225-35.

Yu, L., Han, Z. & Liu, C. (2015) A review on the effects of PBDEs on thyroid and reproduction systems in fish. *General and Comparative Endocrinology*, **219**, 64-73.

Zaheer, K. & Humayoun Akhtar, M. (2017) An updated review of dietary isoflavones: Nutrition, processing, bioavailability and impacts on human health. *Crit Rev Food Sci Nutr*, **57**, 1280-1293.

Zanella, O., Tessaro, I.C. & Féris, L.A. (2014) Desorption- and Decomposition-Based Techniques for the Regeneration of Activated Carbon. *Chemical Engineering & Technology*, **37**, 1447-1459.

Zeilinger, J., Steger-Hartmann, T., Maser, E., Goller, S., Vonk, R. & Lange, R. (2009) Effects of synthetic gestagens on fish reproduction. *Environmental Toxicology and Chemistry*, **28**, 2663-70.

Zhang, Q., Lu, M., Wang, C., Du, J., Zhou, P. & Zhao, M. (2014) Characterization of estrogen receptor alpha activities in polychlorinated biphenyls by in vitro dual-luciferase reporter gene assay. *Environmental Pollution*, **189**, 169-75.

Zhang, T. & Li, B. (2011) Occurrence, Transformation, and Fate of Antibiotics in Municipal Wastewater Treatment Plants. *Critical Reviews in Environmental Science and Technology*, **41**, 951-998.

Zheng, B., Liu, R., Liu, Y., Jin, F. & An, L. (2015) Phenolic endocrine-disrupting chemicals and intersex in wild crucian carp from Hun River, China. *Chemosphere*, **120**, 743-749.

Zoeller, R.T. & Vandenberg, L.N. (2015) Assessing dose–response relationships for endocrine disrupting chemicals (EDCs): a focus on non-monotonicity. *Environmental Health*, **14**, 42.

Zoeller, R.T., Bergman, A.k., Becher, G., Bjerregaard, P., Bornman, R., Brandt, I., Iguchi, T., Jobling, S., Kidd, K.A., Kortenkamp, A., Skakkebaek, N.E., Toppari, J. & Vandenberg, L.N. (2014) A path forward in the debate over health impacts of endocrine disrupting chemicals. *Environmental Health*, **13**, 118-129.

Zuo, Y., Zhang, K. & Deng, Y. (2006) Occurrence and photochemical degradation of 17alpha-ethinylestradiol in Acushnet River Estuary. *Chemosphere*, **63**, 1583-90.

Zuo, Y., Zhang, K. & Zhou, S. (2013) Determination of estrogenic steroids and microbial and photochemical degradation of 17alpha-ethinylestradiol (EE2) in lake surface water, a case study. *Environmental Science Processes and Impacts*, **15**, 1529-35.
